# Design and Synthesis of a Ferrocene‐Based Diol Library and Application in the Hetero‐Diels‐Alder Reaction

**DOI:** 10.1002/chem.202203006

**Published:** 2022-12-08

**Authors:** Laura Cunningham, Patrick J. Guiry

**Affiliations:** ^1^ Centre for Synthesis and Chemical Biology School of Chemistry University College Dublin Belfield Dublin 4 Ireland

**Keywords:** diols, ferrocenes, hetero Diels-Alder reactions, organocatalysis, X-ray crystallography

## Abstract

Diol scaffolds have been utilized as highly effective catalysts and ligands in a wide range of catalytic asymmetric transformations. For scaffolds to be successful as broadly used motifs, they should be prepared cheaply through facile routes and be easily handled. Herein, the synthesis of a family of planar chiral diols based on a modular and robust three‐step route is described, which yields catalytically active diols in >99 % de and >99 % ee, with up to seven different chiral elements. These diols have been characterized by X‐ray crystallographic analysis, which provides clear evidence for the likely transition state when applied in the asymmetric hetero‐Diels‐Alder reaction. Without altering the stereochemistry of the catalyst backbone, it is possible to access both enantiomers of the product by varying the substitution of the catalyst at the α‐position.

## Graphical abstract

**The development of a library of ferrocenyl diols**, establishing a fundamental structure–activity relationship through a modular three‐to‐four‐step synthesis is described. These diols are prepared with complete enantio‐ and diastereoselectivity across seven chiral elements (2 planes, 2 centres, and 3 axes) and are catalytically active in the hetero‐Diels–Alder reaction. Characterization by X‐ray crystallography shows intricate inter‐ and intramolecular hydrogen bonding, as well as uncommon bifurcated hydrogen bonding.

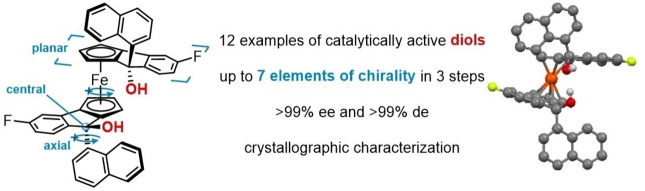

## Introduction

While organocatalysis has emerged as an exceptional synthetic approach in organic chemistry in the last two decades, the major motifs which have dominated the field are amine‐based imine/enamine catalysis,[^1^, ^2^] amino‐thiourea scaffolds[^3^, ^4^] or the more recently developed chiral phosphoric acid systems.^[5]^ However, diol based organocatalysts have contributed significantly to the range of chemical transformations which may be promoted by organocatalysts.^[6]^

**Figure 1:**
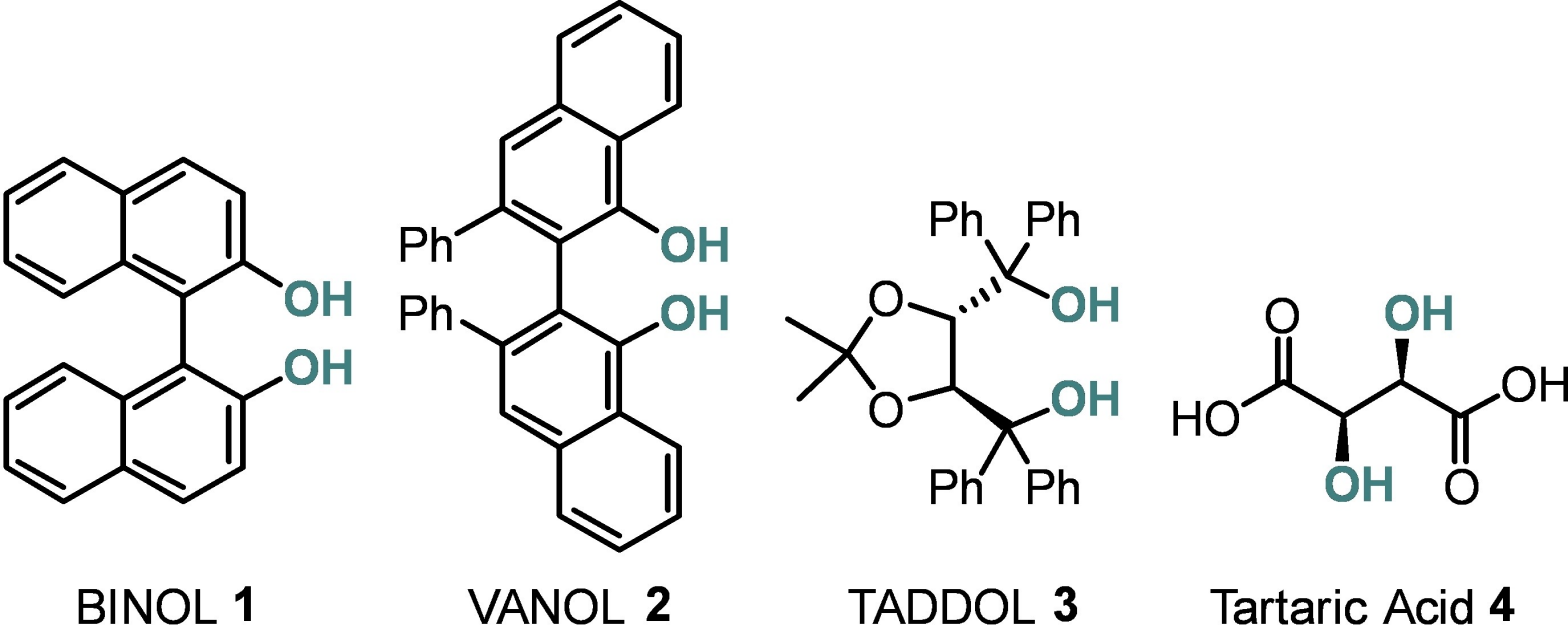
Structures of the leading diol‐based hydrogen‐bond catalysts.

The most familiar of all diol scaffolds is BINOL **1**, but VANOL **2**, TADDOL **3** and tartaric acid derivatives **4** have also been commonly employed as hydrogen‐bond catalysts (Figure 1). While BINOL and TADDOL have served as exceptionally versatile chiral scaffolds in transition‐metal catalysis, the unfettered hydroxy groups can themselves enable chemical transformations.[^6^, ^7^, ^8^]

**Scheme 1:**
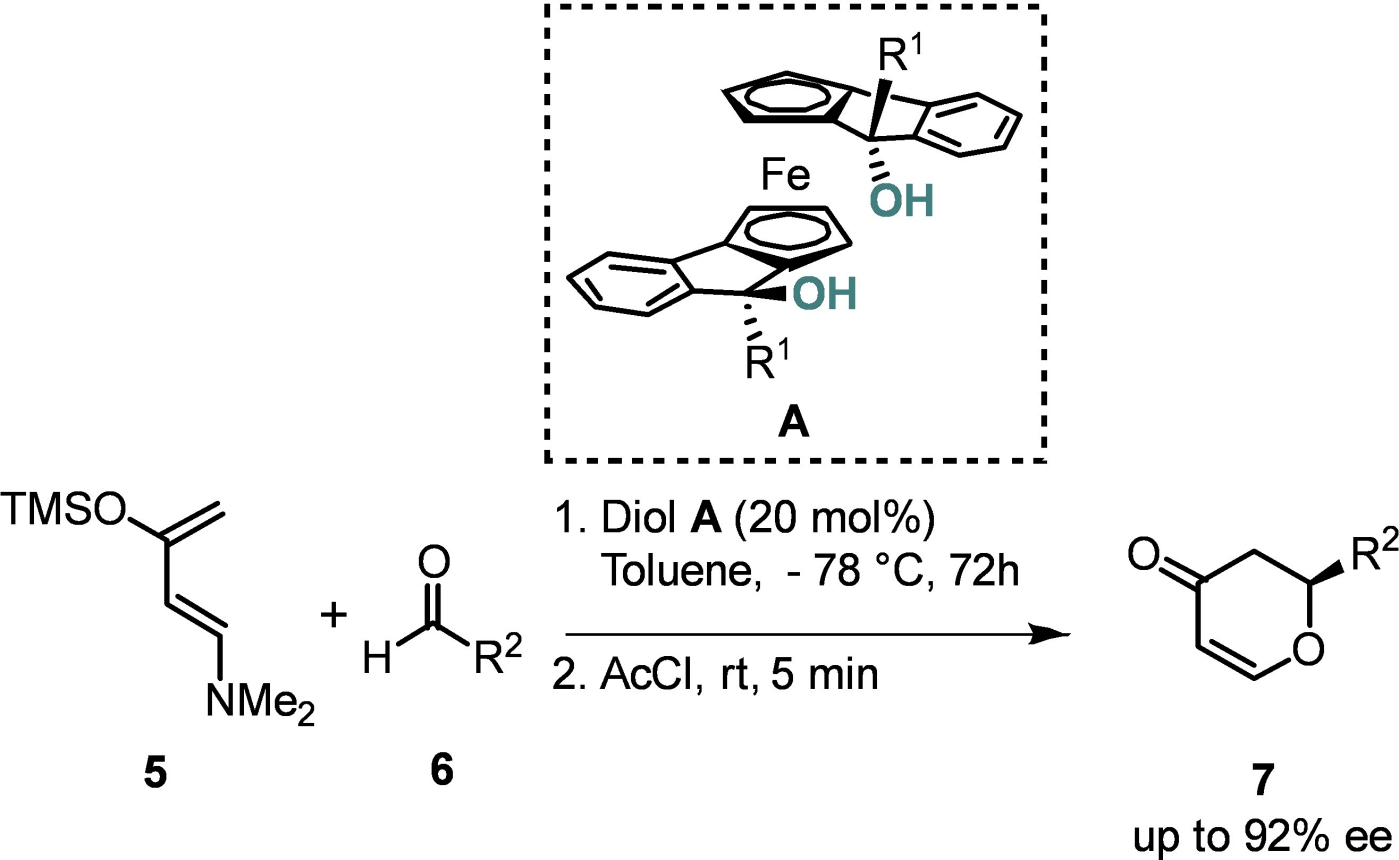
Application of ferrocenyl diols in asymmetric hetero‐Diels‐Alder reaction.

We previously sought to identify a scaffold with a facile synthesis, we considered how to prepare an organocatalyst with the following properties: practically simple and inexpensive synthesis; robust, scalable preparation; bench stable and easily handled. To that end, we prepared a series of ferrocene‐based diols **A** and demonstrated their utility in the hetero‐Diels‐Alder (HDA) reaction, achieving up to 92 % ee when R^1^ and R^2^=1‐naphthyl (Scheme 1)^[9]^ and noted that it fulfilled all of the above criteria associated with privileged ligand design; ferrocene is a cheaply available starting material which is widely exploited in chiral ligand and catalyst design.^[10]^ Furthermore, diols **A** and their precursors are easily prepared, bench stable crystalline powders. However, only changes to the substituent at the α‐position (R^1^) have been investigated to date.

**Scheme 2:**
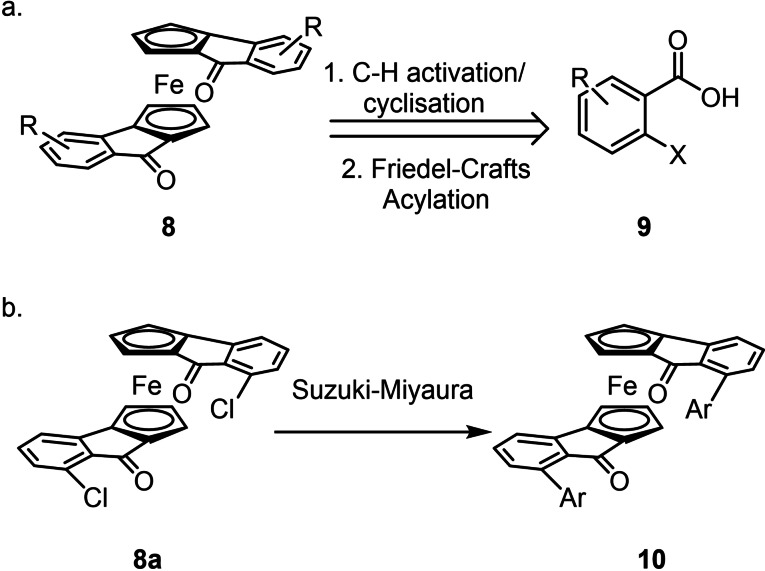
a) Retrosynthetic analysis of diketone intermediates b) Proposed diversification from dichloride **8** **a**.

Here, we seek to expand this library of diols by derivatization of the backbone itself and to design a modular, brief route to access a series of catalysts with a range of steric and electronic properties which will be evaluated to establish a preliminary structure‐activity relationship. This would enable future exploration of this diol family in other asymmetric H‐bond catalysed reactions with ease. To do this, we employ a focused library of *o*‐iodo‐ and *o*‐bromobenzoic acids with various substitution patterns to access a series of diketones **8** as direct precursors to our diol catalysts (Scheme 2a). Further, benzoic acids with an additional halide present may be used for late‐stage Suzuki‐Miyaura couplings to further diversify the target library (e.g. **8** **a**, Scheme 2b).

## Results and Discussion

**Scheme 3:**
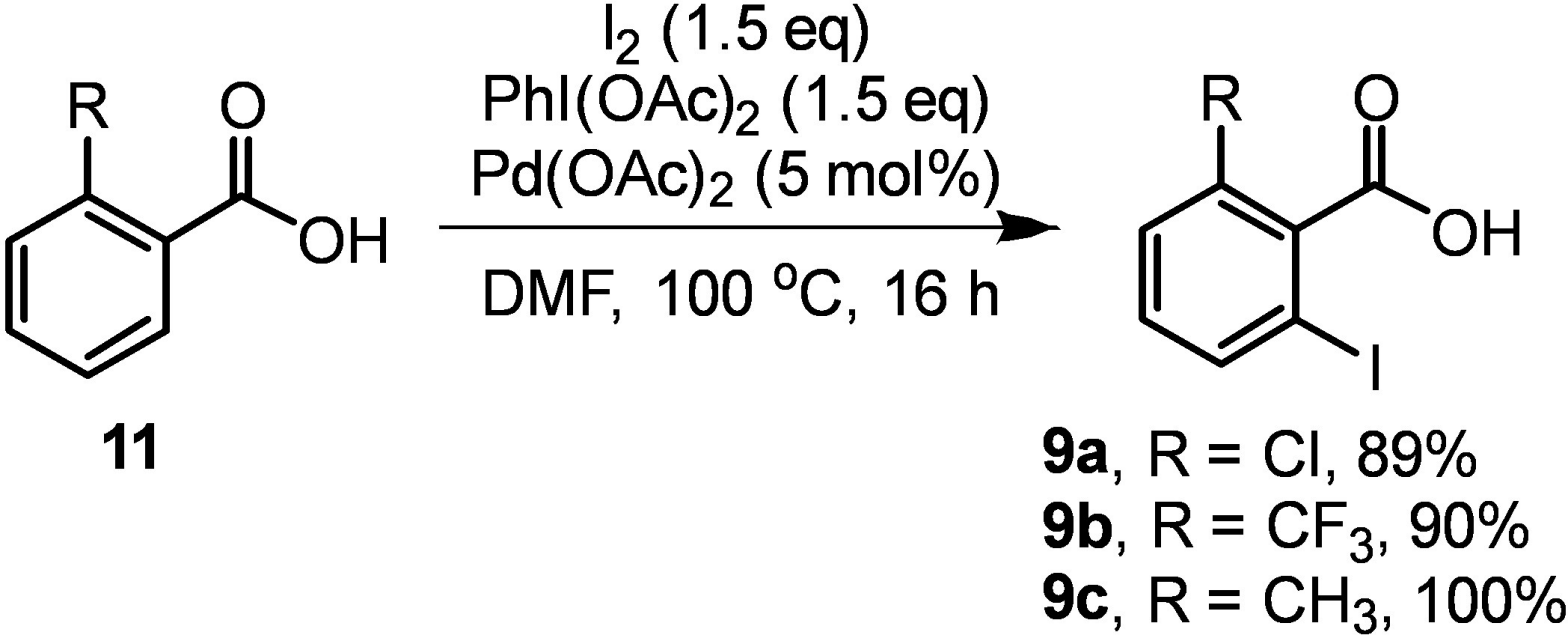
Selective *o*‐iodination of benzoic acids.

While many substituted *o*‐iodobenzoic acids are commercially available, these often do not possess our desired substitution pattern. As such, we sought to selectively *o*‐iodinate a selection of benzoic acids to access the target intermediates. This was generally facile, with products obtained in excellent yields (86–100 %) employing conditions developed by Yu (Scheme 3).^[11]^

**Scheme 4:**
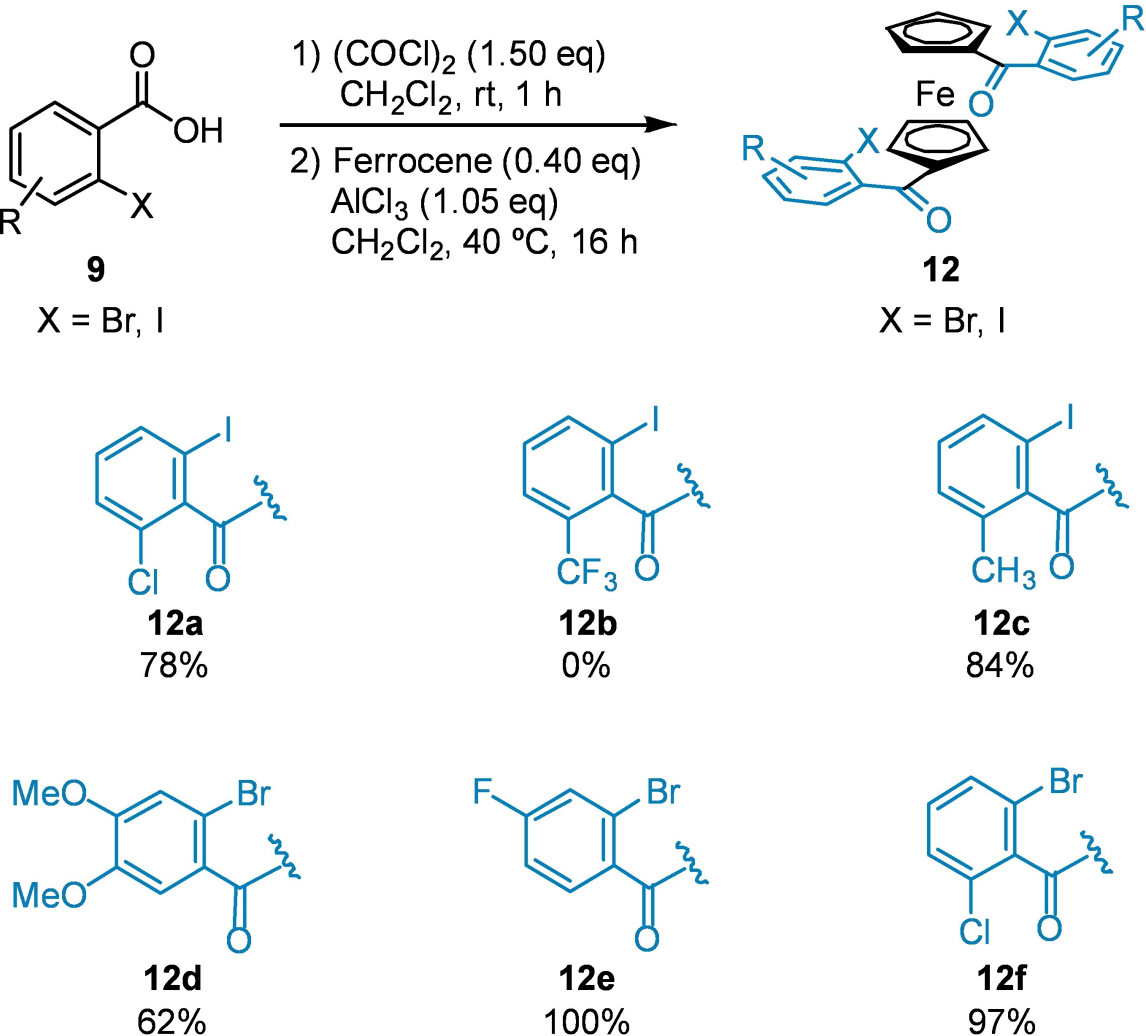
Friedel‐Crafts acylation of ferrocene with in situ generated acyl chlorides.

Next, treatment of each *o*‐halobenzoic acid with oxalyl chloride gives rise to the desired acyl chloride, which is not isolated but immediately treated with aluminium trichloride and ferrocene to undergo a Friedel‐Crafts acylation. It was found that all substrates reacted cleanly over the course of 16 h; while the first acylation occurs completely within 30 minutes, longer reaction times are necessary for conversion to the diacylated products **12** in good yield (Scheme 4). The *o*‐trifluoromethylbenzoic acid failed to furnish the desired product. We propose this is an issue with acyl chloride formation, as opposed to with the Friedel‐Crafts acylation; while all other acyl chlorides were white/cream solids, trifluoromethylated benzoic acid **9** **b** afforded an orange gel after treatment with oxalyl chloride, suggesting decomposition of the intermediate.

**Scheme 5:**
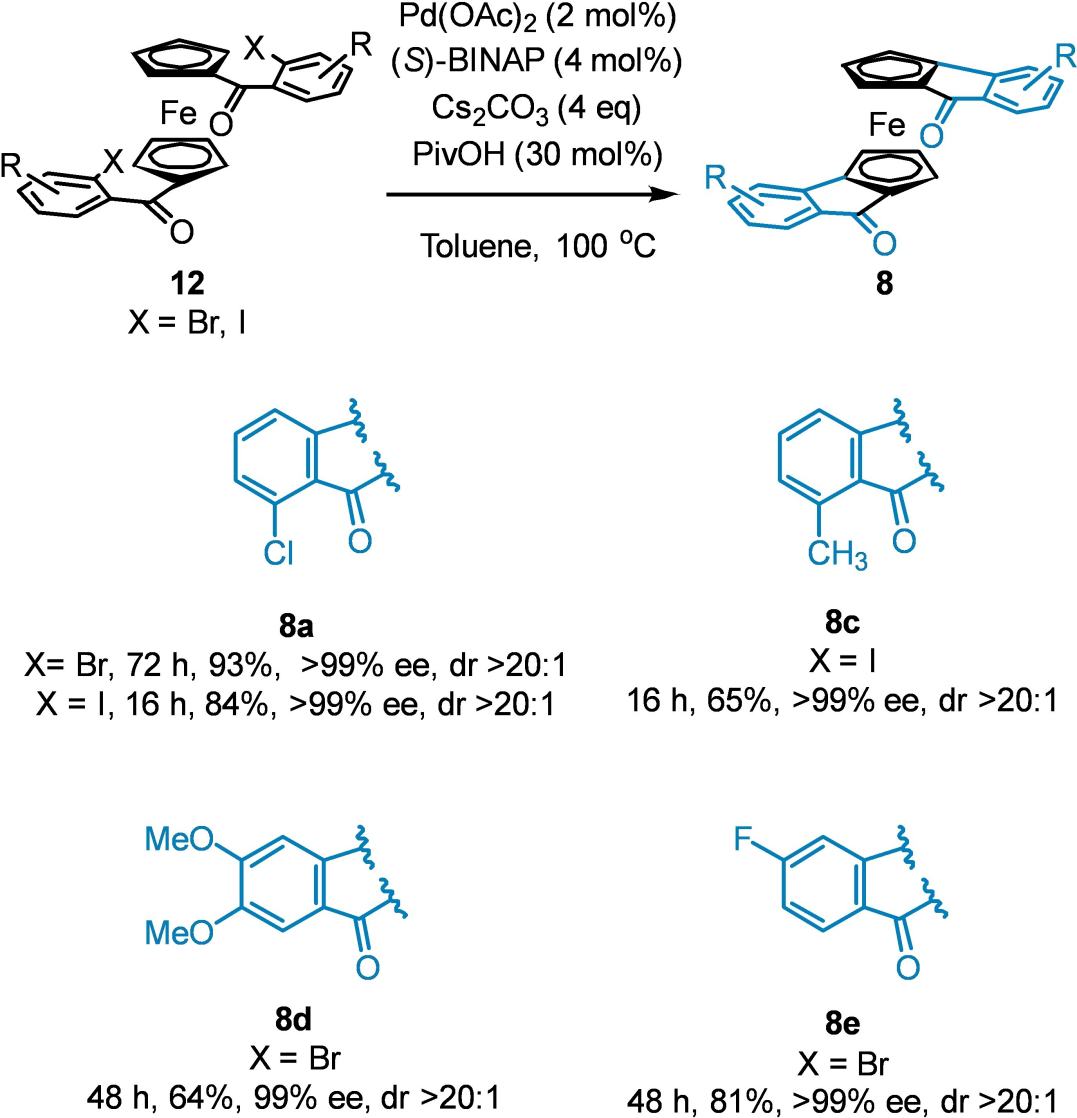
C−H activation/cyclization sequence to access planar chiral diketones.

With our small library of ferrocenyl dihalides in hand, we next sought to investigate the stereoselectivity of the Pd‐catalyzed C−H activation/cyclization previously described independently by the You and Gu groups.[^12^, ^13^] then subsequently optimized as described in our earlier work on this scaffold.^[9]^ In the original report by You, only a single example of a dihalogenated substrate was described, without any substitution, in 99 % ee and complete diastereoselectivity. As such, it was gratifying to see that the presence of substitution around the benzene ring had no deleterious effect on the enantio‐ or diastereoselectivity of the transformation, with effectively perfect enantio‐ and diastereocontrol observed in all cases (Scheme 5).

**Scheme 6:**
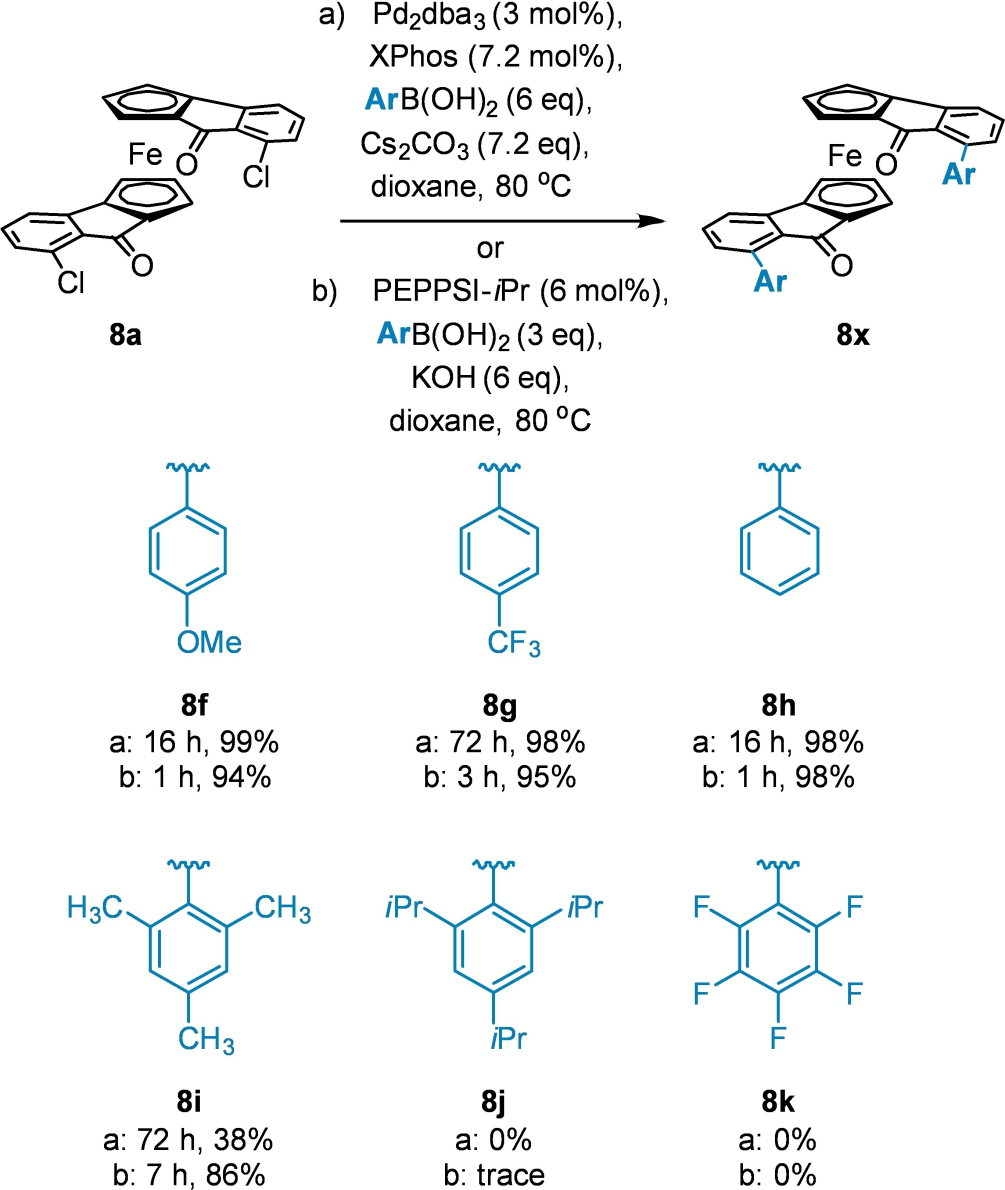
Conditions for the Suzuki‐Miyaura coupling of dichloride **8** **a** with aryl boronic acids.

Shorter reaction times were observed when iodides are used (16 h vs. 48–72 h), but otherwise all substrates reacted cleanly to give one product as a single stereoisomer in excellent ee (Scheme 5). Dichloride **8** **a** was subjected to Suzuki‐Miyaura coupling conditions to produce more sterically demanding arylated scaffolds (Scheme 6). After an initial screening of conditions using phenyl boronic acid, the desired cross coupled product **8** **h** was afforded in good yield (98 %). *p*‐Methoxyphenyl and *p*‐trifluoromethylphenyl boronic acid similarly reacted in excellent yield (99 % and 98 %, respectively). However, mesityl, tri‐*iso*‐propyl and pentafluorophenyl boronic acids were either completely unreactive or afforded the product in only trace amounts, and so further screening of reaction conditions was necessary.

It was found that of the many ligand, solvent and base combinations screened (Supporting Information Table 1), few improved upon the efficiency of condition set (a) (Scheme 6). While replacing XPhos with SPhos enabled shorter reaction times,[^14^, ^15^] no other Buchwald type ligands improved conversion or reaction time. However, condition set (b): using the PEPPSI‐*i*Pr precatalyst (Scheme 6),^[16]^ led to a marked reduction in reaction times (by up to a factor of 16), required fewer equivalents of boronic acid and gave complete conversion in the case of mesityl boronic acid. It was hoped that this rapid reactivity would allow for successful reaction with pentafluorophenyl boronic acid, by outcompeting protodeborylation, but no product was observed.

During the course of these Suzuki‐Miyaura reaction condition screens, an unexpected observation of dehalogenation was observed. We found that when PEPPSI‐*i*Pr, KO*t*Bu and *iso*‐propyl alcohol (IPA) were used in combination, fast and high levels of dehalogenation were seen (82 %). However, when aprotic solvents were employed, this was not observed. Nolan has previously described a Pd‐mediated transfer hydrogenation‐based dehalogenation requiring a sacrificial alcohol,^[17]^ which would rationalize our observation that non‐alcoholic solvents inhibit dehalogenation. Two control reactions were conducted; one in which the Pd‐precatalyst was absent and one in which *t*BuOH was used in place of IPA. In both experiments, dehalogenation was observed, albeit to a lesser degree (50 % and 34 %, respectively), suggesting an alternative mechanism of action.

Some reports show the generation of *tert*‐butoxide radicals from KO*t*Bu and describe phenanthroline as being capable of enabling attenuation of the redox potential of such radicals.[^18^, ^19^] While phenanthroline is not present in our system, we considered the possible role of the pyridine ligand of the precatalyst. Indeed, when the precatalyst is absent, but pyridine is added, 81 % dehalogenation is observed, suggesting it may be involved in the dehalogenation. However, replacing the base with KOH or Cs_2_CO_3_, or using the radical quencher BHT had no effect on the amount of dehalogenation, indicating such a radical based mechanism is not involved. The most significant result observed during these studies was that in the absence of base, no dehalogenation occurs; while there are some examples of base‐mediated dehalogenation of aryl halides in the literature, these are heteroaromatic systems with proposed mechanisms which are not transposable to benzene‐based systems.[^20^, ^21^] At this point, it appears likely that there is a base‐mediated mechanism at play, but the specific details of the mechanism continue to be elusive. Despite the difficulties with coupling pentafluorophenyl or tri‐*iso*‐propylphenylboronic acid, we now had 8 diketones prepared for transformation into our target diols.

**Scheme 7:**
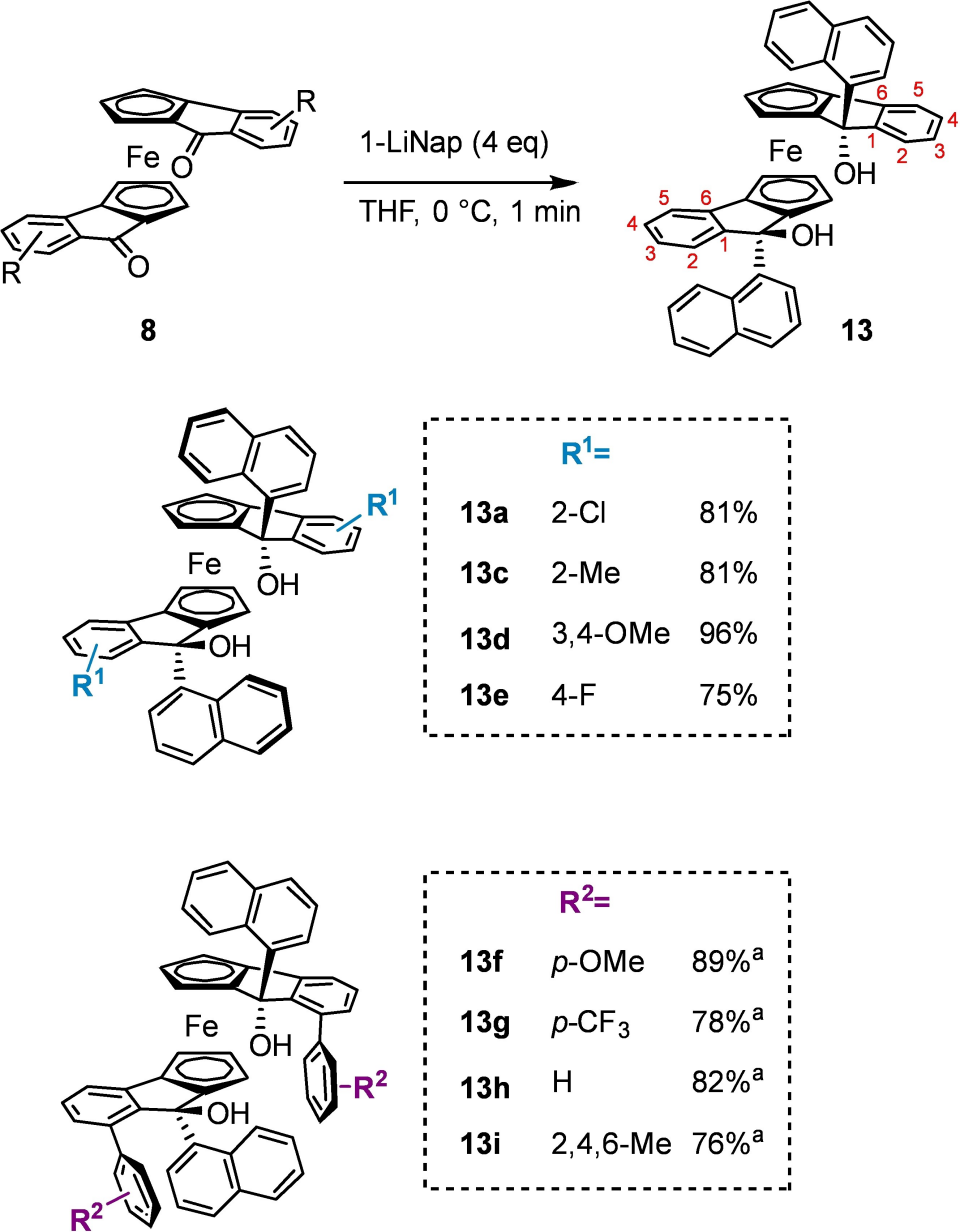
Addition of 1‐naphthyllithium to ferrocenyl diketones. [a] Full conversion of starting material observed to yield product as a mixture of inseparable epimers.

To furnish the final catalysts, C‐nucleophiles were employed to produce the key tertiary alcohols. When adding nucleophiles to systems such as these, it is typical for the incoming nucleophile to approach exclusively from the face opposite the Fe(II) atom enabling formation of the newly formed chiral centre with complete stereospecificity, which is indeed observed in all cases here. We first opted for 1‐naphthyllithium, as this nucleophile yielded the diol which previously achieved the highest levels of enantioselectivity in the HDA reaction.^[9]^ While ketones **8** **a**–**8** **e** cleanly afford the target diols in high yields (75–96 %), the arylated ketones **8** **f**–**8** **i** reacted with high conversion, but gave a complex mixture of products as a result of restricted rotation about the newly formed bond between the diol backbone and the naphthalene ring, giving rise to inseparable epimers (approximately 70 % de in each case; Scheme 7).

The major product obtained is symmetrical, indicating that the sense of axial chirality about the C_α_−C_naph_ bonds are the same on both the top and bottom face, with only 3 ferrocenyl proton signals observed in the ^1^H NMR spectrum. The minor product observed has 6 ferrocenyl proton signals as the axial chirality about the two C_α_−C_naph_ bonds is different, giving a non‐symmetrical epimer (SI figure 1). We tested the barrier to rotation of these epimers and, even at temperatures in excess of 100 °C in DMSO or toluene, no change in d.r. was observed. As such, naphthyl diols derived from the arylated ketones were considered unsuitable as chiral catalysts due to the inevitable competition between the epimeric diols in the mixtures during the HDA reaction.

**Scheme 8:**
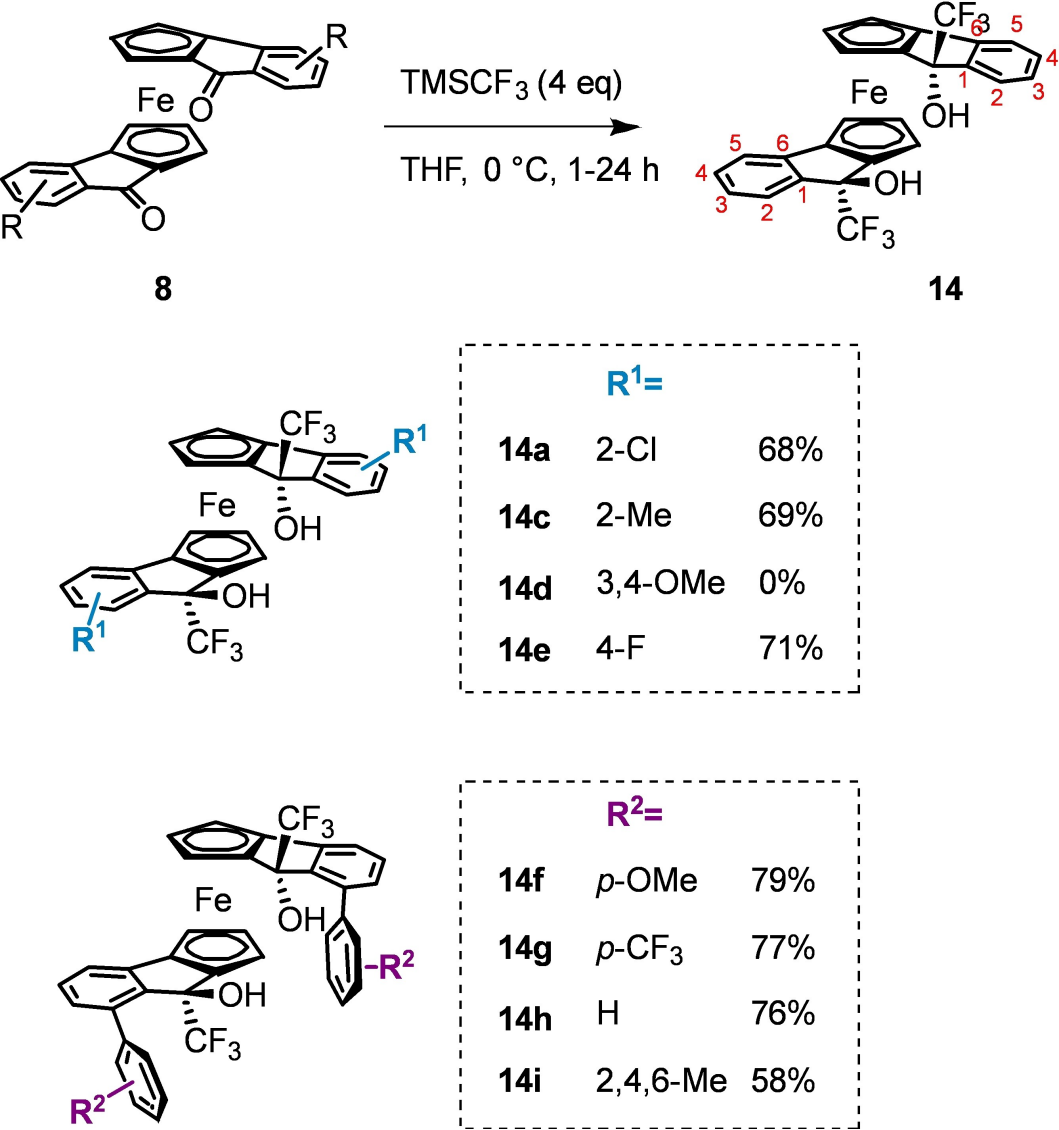
Synthesis of trifluoromethylated diols.

Given the difficulty associated with the addition of particularly bulky nucleophiles, we identified trifluoromethyl as a group likely to be compatible with the steric demand of the arylated ketones **8** **f**–**8** **i**. Employing the Ruppert‐Prakash reagent with TBAF enabled access to the trifluoromethylated diols in good yields (53–79 %) except in the case **8** **d** where only unreacted starting material remained. (Scheme 8). Gratifyingly, the sterically demanding arylated ketones reacted without difficulty, cleanly furnishing the desired products **8** **f**–**8** **i**.

We next sought to briefly examine if the reactivity and enantioselectivity provided by this new library was comparable to our previous library applied in the HDA. While in prior work, enantioselectivities of up to 92 % were observed previously when employing 1‐naphthaldehyde as the substrate, more than half of the ligand/substrate combinations achieved ee values less than 50 %. For example, in the absence of substitution around the catalyst backbone, the trifluoromethylated diol **14** previously achieved a 49 % yield and 47 % ee with benzaldehyde,^[9]^ while naphthyl diol **13** furnished the product in 29 % yield and 68 % ee. In the present work, it was found that these yields and enantioselectivities varied (+/−5 %) when benzaldehyde was employed. We found, however, that *p*‐chlorobenzaldehyde gave consistent results when reactions were carried out in triplicate, and so this was used as the model substrate to evaluate the performance of each ligand.

For comparison, the CF_3_‐ and 1‐naphthyl‐derived diols lacking substitution on the catalyst backbone, from the previous library,^[9]^ were employed in conjunction with Rawal's diene and *p*‐chlorobenzaldehyde, giving yields of 29 % and 47 % and ee values of 50 % and 66 %, respectively (Table 1, entries 1 and 9). An immediately obvious trend is that substitution at the 2‐position is detrimental to the yield of the reaction in all cases, particularly 2‐mesityl, which showed trace product with effectively no asymmetric induction (Table 1, entry 8).

**Table 1:** Application of diols in the HDA reaction.

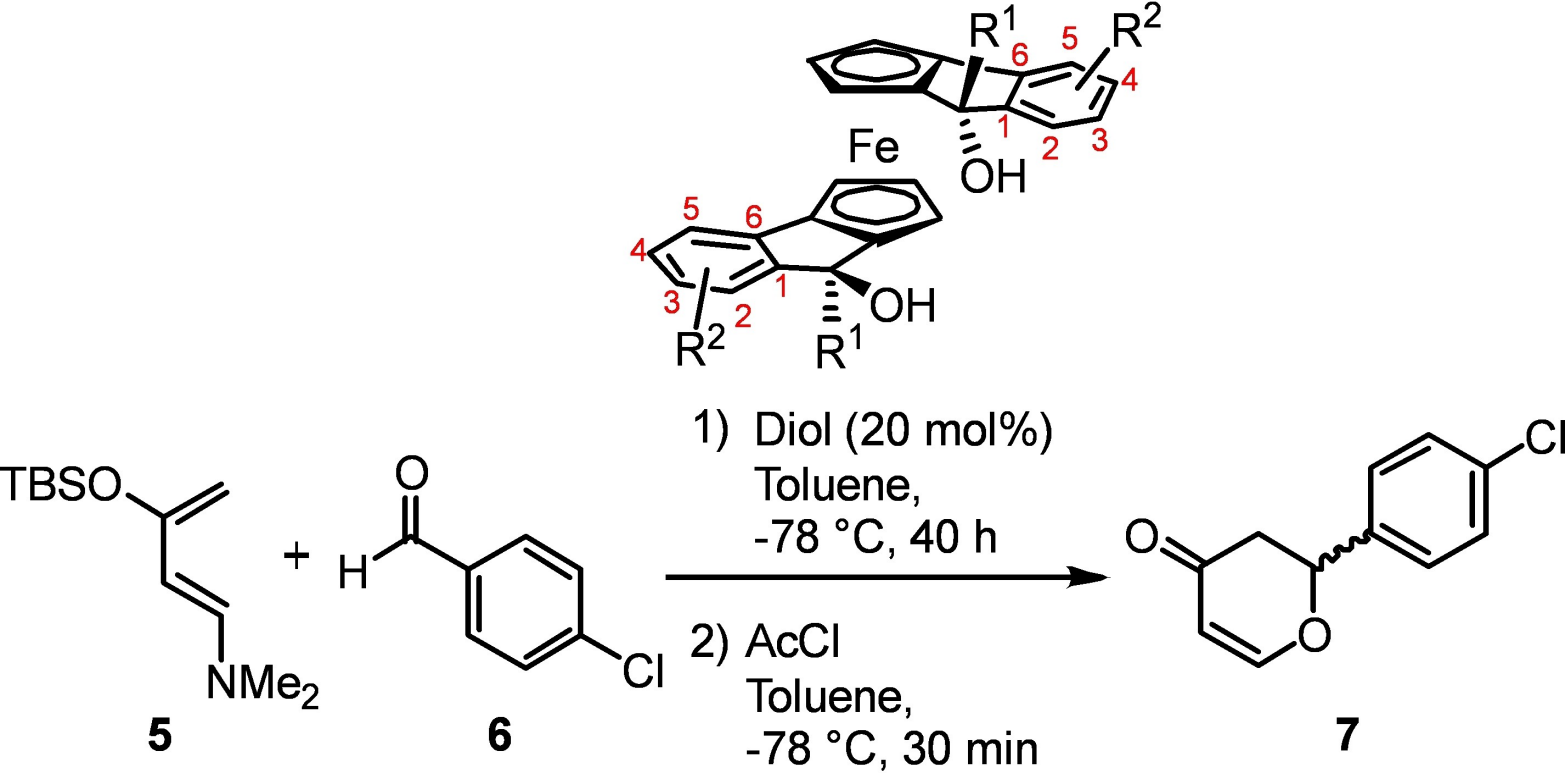					
Entry	Diol	R^1^	R^2^	Yield [%]^[a]^	ee [%]^[b]^
1	**14**	CF_3_	H	29	50
2	**14** **a**	CF_3_	2‐Cl	13	33
3	**14** **c**	CF_3_	2‐Me	19	62
4	**14** **e**	CF_3_	4‐F	36	46
5	**14** **f**	CF_3_	2‐(*p*‐MeOC_6_H_4_)	17	46
6	**14** **g**	CF_3_	2‐(*p*‐CF_3_C_6_H_4_)	12	18
7	**14** **h**	CF_3_	2‐Ph	12	33
8	**14** **i**	CF_3_	2‐mesityl	4	2
9	**13**	1‐naphthyl	H	47	‐66
10	**13** **a**	1‐naphthyl	2‐Cl	17	‐49
11	**13** **c**	1‐naphthyl	2‐Me	9	‐56
12	**13** **d**	1‐naphthyl	3,4‐OMe	27	‐70
13	**13** **e**	1‐naphthyl	4‐F	33	‐60
14	TADDOL	n/a	n/a	36	‐40

[a] Isolated yield. [b] ee value was measured by SFC analysis.

However, although a decrease in yield was observed for catalyst **14** **c**, a marked increase in the enantioselectivity was observed versus the parent ligand (62 % versus 50 %), lending itself to the significance of installing substitution on the catalyst backbone. The 1‐naphthyl‐derived diols induced greater enantioselectivity than their trifluoromethylated counterparts, as was expected. The 3,4‐OMe substituted naphthyl diol **13** **d** achieved a modest improvement in enantioselectivity versus the unsubstituted analogue (Table 1, entry 12 vs. entry 9) It is of note that despite both trifluoromethyl and 1‐naphthyl derived diols possessing the same planar and central chirality, the opposite enantiomer of the product is obtained, as was noted with the original library.^[9]^

**Figure 2:**
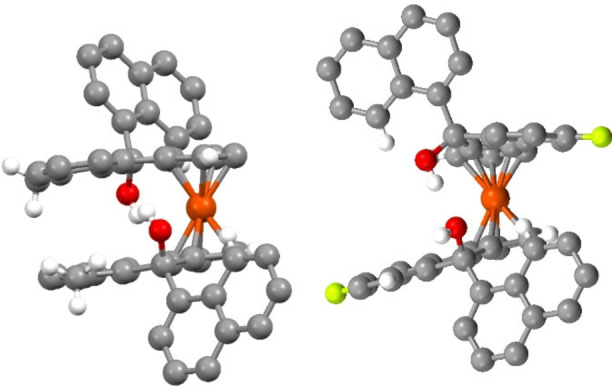
Crystal structures of **13** **c** (left)and **13** **e** (right) highlighting the orientation of the naphthylene rings, suggesting restricted rotation. (Lime=F, Red=O, Orange=Fe, Grey=C, White=H.).

Having now clearly observed that diastereomeric products are obtained upon addition of 1‐naphthyllithium to 2‐arylated ketones **8**, it seems plausible that restricted rotation about the C_α_−C_naph_ bond may also present in the non‐arylated scaffolds. Indeed, X‐ray crystallographic analysis of the methylated naphthyl diol **13** **c** and fluorinated naphthyl diol **13** **e** shows that the orientation of the naphthalene rings would not allow for rotation past the benzene ring of the scaffold due to a clash between the B ring of the naphthalene and the ferrocene ring, or indeed the 2‐substitutent of the benzene ring of the catalyst backbone (Figure 2). As such, it appears likely that the axial chirality at the C_α_−C_naph_ bond is the key contributor that gives rise to the opposite enantiomer of the product in the HDA.

Satisfied that our new catalysts performed similarly to the previous library, we sought to further characterize them by X‐ray crystallographic analysis. It is well understood that catalytically active diols in H‐bond catalysis act via an intramolecular H‐bond between both hydroxy groups, with the remaining hydroxy proton then H‐bonding to the incoming substrate, in this case, *p*‐chlorobenzaldehyde. Given that electron deficient **13** **e** and **14** **e** afford the highest yields of product, likely as a consequence of increased acidity of the hydroxy groups, it is reasonable to assume our diols similarly behave in this manner. Indeed, we observed that, while all of our diols showed hydrogen bonds in the crystal lattice, the fluorinated **13** **e** and **14** **e** showed the characteristic intramolecular hydrogen bonds consistent with the transition state of H‐bond catalysed HDA reactions.

**Figure 3:**
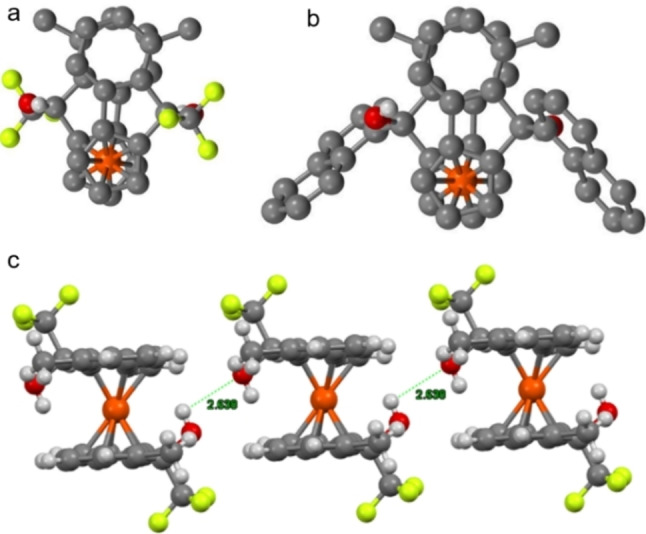
a) Top‐down view of methylated CF_3_ diol **14** **c** b) Top‐down view of methylated 1‐naphthyl diol **13** **c** c) Side‐on view of methylated CF_3_ diol **14** **c** lattice (Lime=F, Red=O, Orange=Fe, Grey=C, White=H.).

**Figure 4:**
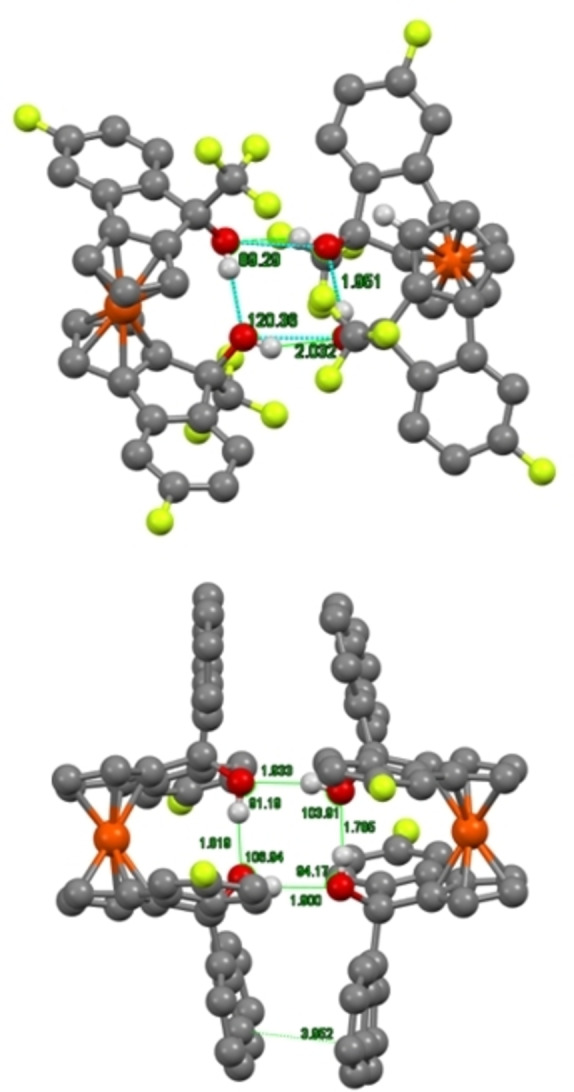
X‐ray crystal structure of diols **14** **e** (top) and **13** **e** (bottom) showing intra‐ and intermolecular H‐bonding. (Lime=F, Red=O, Orange=Fe, Grey=C, White=H).

Many of the crystals obtained show the “closed conformation”, whereby the hydroxy groups are on opposite sides of the molecule, and the two benzene rings of the backbone overlap; with a distance of 3.7 Å between the rings suggesting favourable π‐stacking (Figure 3a, 3b). This conformation gives rise to long chains of diols in the crystal structure, arising from intermolecular hydrogen bonds (Figure 3c). It is therefore feasible to suggest that in the case of the more acidic fluorinated diols **13** **e** and **14** **e**, the stabilization afforded by intramolecular hydrogen bonds is greater than that of potential π‐stacking. Both fluorinated diols give rise to dimeric unit cells, forming a relay of hydrogen bonds between the four hydroxy groups (Figure 4).

Although the trifluoromethylated diol **14** **e** has a twisted square, as is clear from the angles between the oxygens and relative orientation of the two ferrocenyl units, naphthyl diol **13** **e** has a near perfect square, with angles of close to 90°. Significant tilting of the cyclopentadiene rings can be seen in the crystal structure of **13** **e** (Figure 4, bottom), which allows such a conformation to be adopted. This highly organized dimer is potentially enabled by the apparent π‐stacking between the naphthalene rings (distance of 3.95 Å). Given the known mechanism for other diol scaffolds in organocatalyzed‐HDA reactions, it is likely these solid‐state structures represent the conformation of our diols in the transition state.^[22]^

**Figure 5:**
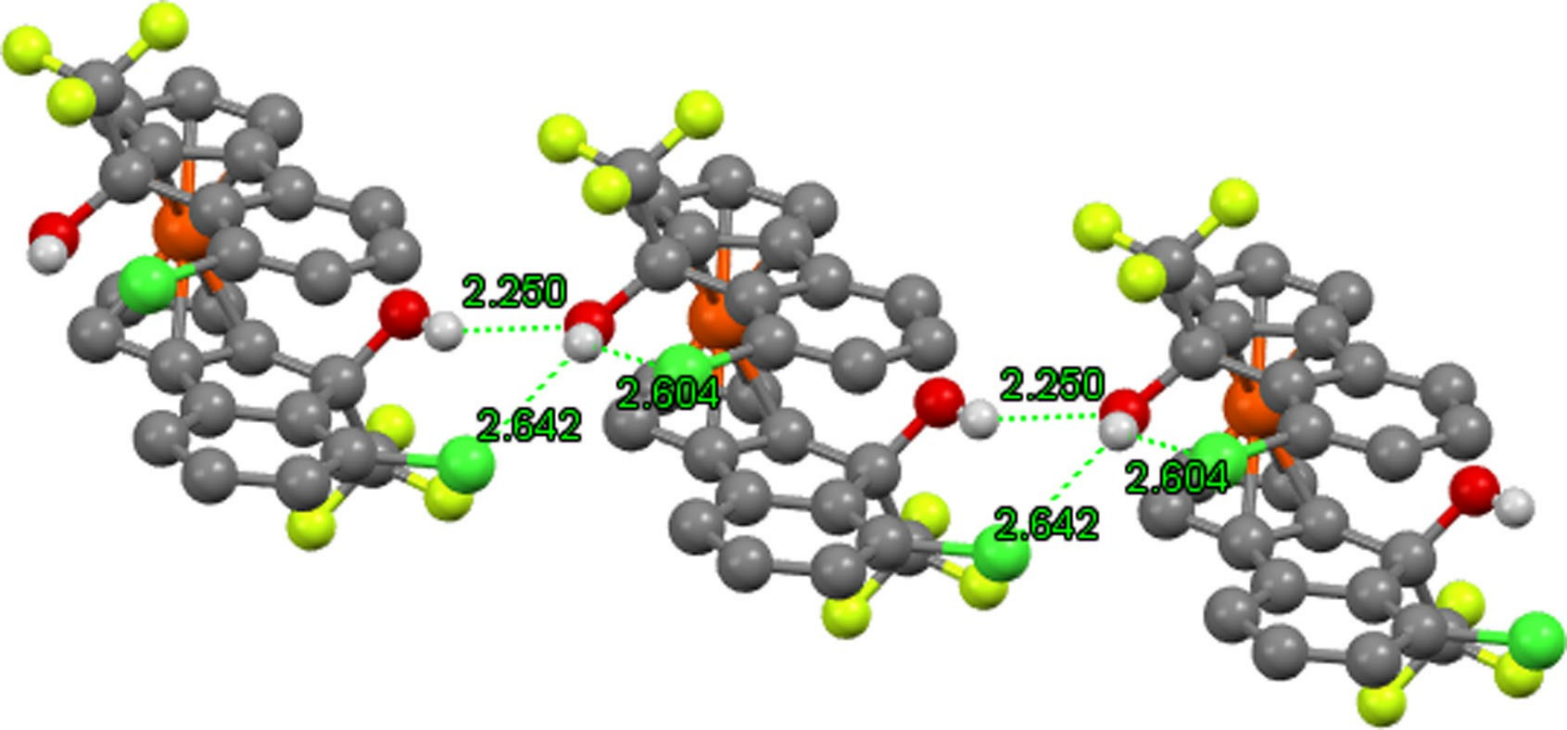
X‐ray crystal structure of three molecules of **14** **a** showing bifurcated hydrogen bonds. (Lime=F, Green=Cl, Red=O, Orange=Fe, Grey=C, White=H.).

An interesting observation noted during this X‐ray crystallographic study was the presence of bifurcated hydrogen bonds. In the case of the 2‐chlorinated trifluoromethyl diol **14** **a**, intricate networks of hydrogen bonding were observed, in which each hydroxy group of a single molecule are in chemically distinct environments (Figure 5). One hydroxy group acts as a typical intermolecular hydrogen bond donor to the hydroxy group of a neighbouring diol. The other hydroxy group accepts a hydrogen bond through the oxygen but donates two hydrogen bonds to two chlorine atoms by the hydroxy hydrogen: one intramolecular H‐bond to the chloride of the same backbone, and one intermolecular H‐bond to the chloride of the neighbouring diol. Bifurcated H‐bonds are mostly observed in macromolecular systems with elaborate hydrogen bonding networks (such as proteins and DNA),[^23^, ^24^] but there are some examples of this phenomenon in small molecules and metal complexes.[^25^, ^26^]

**Figure 6:**
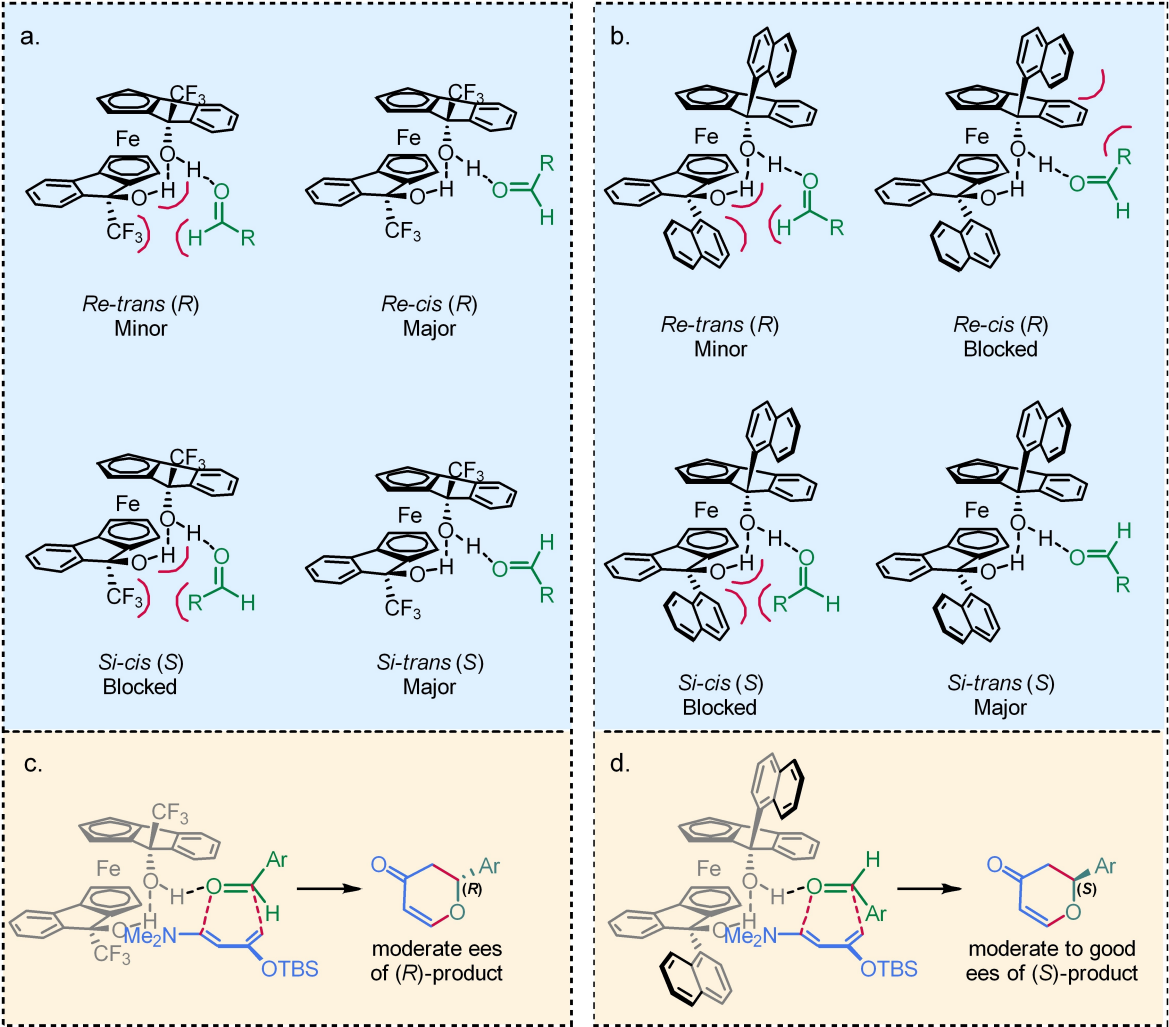
a) Proposed coordination geometries of aldehyde with trifluoromethylated diols. b) Proposed coordination geometries of aldehyde for 1‐naphthyl diols. c) Proposed major transition state for trifluoromethylated diols in the HDA d) Proposed major transition state for 1‐naphthyl diols in the HDA.

With these new observations regarding the likely conformation of our catalysts in the transition states, we can propose with more confidence our explanation for the observed stereochemical outcome of the HDA. The proposed transition states for the non‐naphthyl derived diols remains unchanged versus our previous proposal;^[9]^ two transitions states, *Re*‐*cis* and *Si*‐*trans*, are both likely to occur during catalysis (Figure 6a). The dominance of the (*R*)‐enantiomer of the product arises due to the fact the *Re*‐*trans* and *Re‐cis* transition states both generate the (*R*)‐product, while the *Si*‐*cis* is blocked, limiting the amount of (*S*)‐enantiomer generated (Figure 6a). In the case of naphthyl diols, X‐ray crystallographic analysis suggests that the naphthalene rings are projected away from the catalyst backbone, providing good evidence that the *Re*‐*cis* transition state would be almost completely inaccessible, and so *Si‐trans* becomes the dominant transition state, yielding the (*S*)‐product in good ee (Figure 6b).

A suggested approach for the diene is shown in Figure 6c and 6d, which we anticipate being unaffected by changes to the catalyst substitution. Approach from the back‐face will be inhibited by the catalyst backbone. When the diene approaches the aldehyde, the orientation in which the bulky TBS group is furthest from the catalyst is presumably favoured, with the smaller dimethylamine group occupying the space nearest the catalyst.

## Conclusion

We described the chromatography‐free synthesis of a library of ferrocenyl ketones **8** in two to three steps with overall yields of 77–91 % from a series of substituted benzoic acids. Each of these steps can be carried out on a multigram scale and employ cheaply available reagents, yielding products in >99 % ee and >99 % de. These ketones, possessing a range of substitution about the C‐ring, represent a powerful synthetic intermediate which were easily derivatized to yield 12 catalytically active chiral diols, with excellent stereocontrol of up to seven aspects of chirality (two centres, two planes and three axes in the case of naphthyl diols). While only modest enantioselectivity and yields were observed in the hetero‐Diels‐Alder reaction for some of the diols prepared in the present work, we highlight that these results are equivalent to, and in some cases, improvements upon, the results obtained from the use of TADDOL, a leading diol catalyst, suggesting this family of catalysts may similarly find significant success in other transformations. A plausible trend, in which substitution of the backbone influences catalytic activity has been observed; diols with increased acidity giving rise to increased conversions as a consequence of stronger hydrogen bond donation.

X‐ray crystallographic analysis of these diols has provided evidence for our proposed transition state of the H‐bond catalyzed HDA reaction and revealed a likely explanation for the stereochemical switch observed when 1‐naphthyl diols are employed. The observation of notably uncommon bifurcated hydrogen bonds has also been described. Having now established a fundamental understanding of the influence of substitution around the catalyst backbone on the hetero‐Diels‐Alder reaction, we now seek in future work to explore the synthesis of new applications of these systems in H‐bond catalysis and as bidentate O,O‐ligands in transition metal catalysis.

## Experimental Section

**General procedure for Friedel‐Crafts acylation of ferrocene**: 2‐halobenzoic acid (2.5 equiv) was dissolved in CH_2_Cl_2_ in a dry Schlenk flask under nitrogen and placed in an ice bath. 3 drops of DMF were added followed by oxalyl chloride (3 equiv) which was added slowly over 2 min. The reaction was stirred at rt for 1 h, after which time solvent and residual oxalyl chloride were removed by vacuum. After drying for 1 h under vacuum, the crude acid chloride was dissolved in CH_2_Cl_2_ to which ferrocene (1 equiv) was added followed by additional CH_2_Cl_2_. AlCl_3_ (2.1 equiv) was added in four portions and the reaction was refluxed until complete. The reaction was quenched with ice water (50 mL). The layers were separated, and the aqueous layers was back‐extracted with CH_2_Cl_2_.The combined organic layers were washed with 2.0 M NaOH, dried over MgSO4, filtered and dried in vacuo and used was isolated without further purification.

**General procedure for C−H activation/cyclisation**: dihalogenated benzoyl ferrocene (1 equiv was added to a dry Schlenk flask under nitrogen. Cs_2_CO_3_ (4 equiv), Pd(OAc)_2_ (2 mol%), (*S*)‐BINAP (4 mol%), and pivalic acid (0.3 equiv) were added sequentially. Toluene was added and the reaction was stirred at 100 °C until complete. The reaction was cooled to room temperature then filtered through a pad of celite using CH_2_Cl_2_. The solution was concentrated *in vacuo* to afford the di‐cyclised product as a fine powder which was used without further purification.

**General procedure for Suzuki‐Miyaura cross coupling of dichloride 8** **a**: 2‐chloro‐diketone **8** **a** (1 equiv), PEPPSI‐*i*Pr (18 mg, 0.026 mmol, 4 mol%) 4‐trifluoromethylphenyl boronic acid (3 equiv) and KOH (6 equiv) were combined in a flame dried Schlenk flask under nitrogen_._ 1,4‐Dioxane was added, and the reaction was stirred at room temperature until complete. The reaction mixture was cooled to room temperature and filtered through a pad of Celite with Et_2_O and concentrated. The resulting red oil was purified through a silica plug (40 % EtOAc/cHex), to afford the desired cross coupled product which was used without further purification.

**General procedure for the synthesis of 1‐naphthyl diols**: 1‐bromonaphthalene (4 equiv) was added to a flame dried, nitrogen flushed Schlenk flask equipped with a magnetic stirring bar and dissolved in THF. The flask was cooled to −78 °C in a liquid nitrogen/acetone bath and *n*BuLi (4 equiv) was added dropwise. After 10 min at −78 °C a solution of diketone (1 equiv) in THF was added and the reaction allowed to warm to room temperature over 5 min. The reaction was quenched with sat. aq. NaHCO_3_, the organic and aqueous layers were separated and the aqueous layer was back‐extracted with Et_2_O. The combined organic layers were dried (Na_2_SO_4_), filtered and concentrated in vacuo followed by silica gel chromatography (CH_2_Cl_2_) to give the product as an orange powder.

**General procedure for the synthesis of trifluoromethylated diols**: Diketone (1 equiv) and TMSCF_3_ (5 equiv) were added to a dry 2‐neck round bottom under nitrogen. THF was added, and the resulting red solution was cooled in an ice bath with stirring. TBAF (0.1 mL, 1 M in THF) was added and the reaction mixture was stirred until complete by TLC analysis at 0 °C. The remaining TBAF (5 equiv) was added slowly at 0 °C and the reaction was warmed to room temperature over 10 min. The reaction was quenched with H_2_O, the organic and aqueous layers were separated, and the aqueous layer was back‐extracted with Et_2_O. The combined organic layers were dried over Na_2_SO_4_, filtered and concentrated. The resulting oil was purified by SiO_2_ chromatography affording the desired product as an orange powder.

**General procedure for hetero‐Diels‐Alder reaction**: The diol catalyst (0.05 mmol, 0.2 equiv) was added to a dry, N_2_ flushed 10 mL Schlenk flask equipped with a magnetic stirring bar followed by 2 mL of anhydrous toluene. The aldehyde (0.50 mmol, 2.0 equiv) was added with stirring at RT before cooling to −78 °C. After 30 min the diene (65 μL, 0.25 mmol, 1.0 equiv) was added and the reaction was left to stir for 48 h. The reaction was quenched at −78 °C by the addition of acetyl chloride (70 μL, 1.0 mmol, 4.0 equiv) followed by dilution with CH_2_Cl_2_ (1 mL). The crude reaction mixture was purified directly by SiO_2_ chromatography (8 : 2 cHex/EtOAc).

## Supporting Information

Additional X‐ray crystal structures for 13c, 13 f, 14a, 14c, 14e and 14 f are included in the Supporting Information. Deposition Numbers 2209174, 2209175, 2209176, 2209177, 2209178, 2209179 contain the supplementary crystallographic data for this paper. These data are provided free of charge by the joint Cambridge Crystallographic Data Centre and Fachinformationszentrum Karlsruhe Access Structures service.

## Conflict of interest

The authors declare no conflict of interest.

1

## Supporting information

As a service to our authors and readers, this journal provides supporting information supplied by the authors. Such materials are peer reviewed and may be re‐organized for online delivery, but are not copy‐edited or typeset. Technical support issues arising from supporting information (other than missing files) should be addressed to the authors.

Supporting Information

## Data Availability

The data that support the findings of this study are available in the supplementary material of this article.

## References

[1] Y.-Q. Zou , F. M. Hörmann , T. Bach , Chem. Soc. Rev. 2018, 47, 278. 29155908 10.1039/c7cs00509a PMC5789435

[2] F. Peng , Z. Shao , J. Mol. Catal. A 2008, 285, 1–13.

[3] W. Y. Siau , J. Wang , Catal. Sci. Technol. 2011, 1298–1310.

[4] A. Madarász , Z. Dósa , S. Varga , T. Soós , A. Csámpai , I. Pápai , ACS Catal. 2016, 6, 4379–4387.

[5] R. Maji , S. C. Mallojjala , S. E. Wheeler , Chem. Soc. Rev. 2018, 47, 1142–1158. 29355873 10.1039/c6cs00475j

[6] N. T. Nguyen , P. A. Chen , K. Setthakarn , A. J. May , Molecules 2018, 23, 2317–2329. 30208621 10.3390/molecules23092317 PMC6225256

[7] S. Lou , P. N. Moquist , S. E. Schaus , J. Am. Chem. Soc. 2006, 128, 12660–12661. 17002355 10.1021/ja0651308

[8] S. Lou , P. N. Moquist , S. E. Schaus , J. Am. Chem. Soc. 2007, 129, 15398–15404. 18020334 10.1021/ja075204v PMC2638762

[9] C. Nottingham , H. Müller-Bunz , P. J. Guiry , Angew. Chem. Int. Ed. 2016, 55, 11115–11119; 10.1002/anie.201604840 27418323

[10] L. Cunningham , A. Benson , P. J. Guiry , Org. Biomol. Chem. 2020, 18, 9329–9370. 33155613 10.1039/d0ob01933j

[11] T. S. Mei , R. Giri , N. Maugel , J. Q. Yu , Angew. Chem. Int. Ed. 2008, 47, 5215–5219; 10.1002/anie.200705613 18523942

[12a] D. W. Gao , Q. Yin , Q. Gu , S. L. You , J. Am. Chem. Soc. 2014, 136, 4841–4844; 24625115 10.1021/ja500444v

[12b] R. Deng , Y. Huang , X. Ma , G. Li , R. Zhu , B. Wang , Y. B. Kang , Z. Gu , J. Am. Chem. Soc. 2014, 136, 4472–4475. 24617772 10.1021/ja500699x

[13a] Z.-Z. Zhang , D.-Y. Huang , B. F. Shi , Org. Biomol. Chem. 2022, 20, 4061–4073; 35521690 10.1039/d2ob00558a

[13b] C.-X. Liu , G. Qing , S.-L. You , Trends Chem. 2020, 2, 737–750;

[13c] D.-W. Gao , Q. Gu , C. Zheng , S.-L. You , Acc. Chem. Res. 2017, 50, 351–365; 28121428 10.1021/acs.accounts.6b00573

[13d] Z.-J. Cai , C.-X. Liu , Q. Gu , C. Zheng , S.-L. You , Angew. Chem. Int. Ed. 2019, 58, 2149–2153; 10.1002/anie.201813887 30589183

[13e] C.-X. Liu , Z.-J. Cai , Q. Wang , Z.-J. Wu , Q. Gu , S.-L. You , CCS Chemistry 2020, 2, 642–651.

[14] T. E. Barder , S. D. Walker , J. R. Martinelli , S. L. Buchwald , J. Am. Chem. Soc. 2005, 127, 4685–4696. 15796535 10.1021/ja042491j

[15] D. S. Surry , S. L. Buchwald , Chem. Sci. 2011, 2, 27–50. 22432049 10.1039/C0SC00331J PMC3306613

[16] T. Kinzel , Y. Zhang , S. L. Buchwald , J. Am. Chem. Soc. 2010, 132, 14073–14075. 20858009 10.1021/ja1073799 PMC2953245

[17] M. S. Viciu , G. A. Grasa , S. P. Nolan , Organometallics 2001, 20, 3607–3612.

[18] R. Ueno , T. Shimizu , E. Shirakawa , Synlett 2016, 27, 741–744.

[19] J. P. Barham , G. Coulthard , K. J. Emery , E. Doni , F. Cumine , G. Nocera , M. P. John , L. E. A. Berlouis , T. McGuire , T. Tuttle , J. A. Murphy , J. Am. Chem. Soc. 2016, 138, 7402–7410. 27183183 10.1021/jacs.6b03282

[20] L. Jedinák , R. Zátopková , H. Zemánková , A. Šustková , P. Cankař , J. Org. Chem. 2017, 82, 157–169. 27997179 10.1021/acs.joc.6b02306

[21] T.-H. Ding , J.-P. Qu , Y.-B. Kang , Org. Lett. 2020, 22, 3084–3088. 32227906 10.1021/acs.orglett.0c00827

[22] P. Christ , A. G. Lindsay , S. S. Vormittag , J. M. Neudörfl , A. Berkessel , A. C. O'Donoghue , Chem. Eur. J. 2011, 17, 8524–8528. 21714022 10.1002/chem.201101157

[23] P. Hobza , J. Am. Chem. Soc. 1994, 116, 709–714.

[24] K. Manikandan , S. Ramakumar , Proteins Struct. Funct. Genet. 2004, 56, 768–781. 15281129 10.1002/prot.20152

[25] M. Sojka , J. Tousek , Z. Badri , C. Foroutan-Nejad , M. Necas , Polyhedron 2019, 170, 593–601.

[26] S. F. Bureiko , N. S. Golubev , K. Pihlaja , J. Mol. Struct. 1999, 480, 297–301.

