# Supplementary material for: Design and Synthesis of a Ferrocene‐Based Diol Library and Application in the Hetero‐Diels‐Alder Reaction
Source: Chemistry. 2022 Dec 8;29(6):e202203006. doi: 10.1002/chem.202203006 (PMC10108280; doi:10.1002/chem.202203006)
Supplement: Supplementary file 1 — Supporting Information [file CHEM-29-0-s001.pdf]

Chemistry–A European Journal

Supporting Information

Design and Synthesis of a Ferrocene-Based Diol Library and Application in the Hetero-Diels-Alder Reaction

Laura Cunningham and Patrick J. Guiry*

Supporting Information

Table of Contents

General Experimental Information …..……………………………………………………… 2

Experimental procedures and physical/spectroscopic data ……………………………………. 3

Table SI1. Control reactions for investigation of the mechanism of dehalogenation of 2-chloro diketone ……..…………… ……………………………………......................…………… 22

Figure S1. ^{1}H NMR Spectrum of 2-mesityl naphthyl diol……………………………………. 23

General Procedure for hetero-Diels-Alder Reaction…………………………………………… 24

^{1}H NMR, ^{13}C NMR and ^{31}P NMR Spectra of New Compounds..…………… …..................… 25

SFC Chromatograms of Racemic and Enantioenriched Compounds……..…………………..… 42

X-Ray Crystallographic Structures……..……………………... 48

References…………………………………………………………………………………... 60

General Information: Unless otherwise noted, all commercial reagents were used as received without further purification. Standard Schlenk line techniques were employed for moisture sensitive reactions. Column chromatography was performed on Davisil LC60A 40-63 micron silica gel. Thin-layer chromatography (TLC) was performed on aluminium-backed sheets purchased from Merck pre-coated with silica gel 60 F_{254}. ^{1}H NMR spectra were recorded on Varian-Inova spectrometers (300, 400,) using tetramethylsilane as an internal standard. ^{13}C NMR spectra were recorded on 400 MHz Varian-Inova spectrometers (101 MHz) using tetramethylsilane as an internal standard and were proton decoupled. ^{19}F NMR spectra were recorded on 400 MHz Varian-Inova spectrometers (376 MHz). HRMS were measured on a Micromass/Waters LCT mass spectrometer. Supercritical fluid chromatography (SFC) was performed on a Waters Acquity UPC^{2}® instrument with Chiralpak® IA3, IB3, IC3 and ID3 columns. Optical rotation measurements were recorded using a Schmidt-Haensch Unipol L2000 polarimeter at 589 nm and are quoted in units of deg dm^{-1} cm^{3} g^{-1} (concentration c is given in g/100 mL).

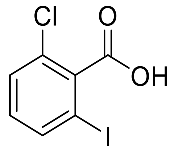

2-Chloro-6-iodobenzoic acid 9a: 2-Chlorobenzoic acid (1.14 g, 7.3 mmol), iodobenzene diacetate (2.35 g, 7.3 mmol), $Pd(OAc)_2$ (82 mg, 0.36 mmol, 5 mol%) and iodine (1.85 g, 7.3 mmol) were combined in a dry 250 mL Schlenk flask under nitrogen and dissolved in DMF (36.5 mL). The reaction was stirred at 100 ^{o}C for 16 h and diluted with EtOAc (100 mL). This solution was washed with 10% HCl (3 x 30 mL) and sat. sodium thiosulfate solution (3 x 200 mL), and the organic layer was dried over $MgSO_4$ and concentrated. The resulting yellow oil was purified by column chromatography (1% formic acid in 8:2 CH_2Cl_2/EtOAc) to afford the product as an off-white solid (1.83 g, 89%). ^{1}H NMR (400 MHz, $CDCl_3$) δ 9.57 (s, br, 1H), 7.76 (dd, *J* = 8.0, 1.0 Hz, 1H), 7.42 (dd, *J* = 8.0, 1.0 Hz, 1H), 7.07 (t, *J* = 8.0 Hz, 1H). All physical data were identical to those previously reported.[1]

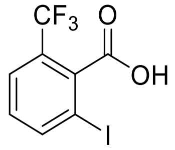

2-Iodo-6-trifluoromethylbenzoic acid 9b: 2-Trifluoromethylbenzoic acid (1.38 g, 7.3 mmol), iodobenzene diacetate (2.35 g, 7.3 mmol), $Pd(OAc)_2$ (82 mg, 0.36 mmol 5 mol%) and iodine (1.85 g, 7.3 mmol) were combined in a dry 250 mL Schlenk flask under nitrogen and dissolved in DMF (36.5 mL). The reaction was stirred at 100 ^{o}C for 16 h and diluted with EtOAc (100 mL). This solution was washed with 10% HCl (3 x 30 mL) and sat. sodium thiosulfate solution (3 x 200 mL), and the organic layer was dried over $MgSO_4$ and concentrated. The resulting yellow oil was purified by column chromatography (1% formic acid in 8:2 CH_2Cl_2/EtOAc) to afford the product as an off-white solid (2.08 g, 90%). ^{1}H NMR (400 MHz, CD_3OD) δ 8.14 (d, *J* = 8.0 Hz, 1 H), 7.72 (d, *J* = 8.0 Hz, 1H), 7.30 (t, *J* =8.0 Hz, 1H). All physical data were identical to those previously reported.[1]

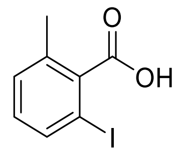

2-Iodo-6-methylbenzoic acid 9c: *o*-Toluic acid (1.0 g, 7.3 mmol), iodobenzene diacetate (2.35 g, 7.3 mmol), $Pd(OAc)_2$ (82 mg, 0.36 mmol 5 mol%) and iodine (1.85 g, 7.3 mmol) were combined in a dry 250 mL Schlenk flask under nitrogen and dissolved in DMF (36.5 mL). The reaction was stirred at 100 °C for 16 h, and diluted with EtOAc (100 mL). This solution was washed with 10% HCl (3 x 30 mL) and sat. sodium thiosulfate solution (3 x 200 mL), and the organic layer was dried over $MgSO_4$ and concentrated. The resulting yellow oil was purified by column chromatography (1% formic acid in 8:2 CH_2Cl_2/EtOAc) to afford the product as an off-white solid (2.04 g, 100%). ^1H NMR (400 MHz, $CDCl_3$) δ ^1H NMR (400 MHz, $CDCl_3$) δ 7.69 (d, *J* = 7.8 Hz, 1H), 7.21 (d, *J* = 7.8 Hz, 1H), 7.02 (t, *J* = 7.8 Hz, 1H), 2.45 (s, 3H). All physical data were identical to those previously reported.[1]

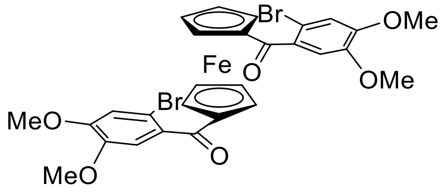

1,1'-Di(2-bromo-4,5-dimethoxybenzoyl)ferrocene 12d: 2-Bromo-4,5-dimethoxybenzoic acid (6.60 g, 25.3 mmol) was dissolved in CH_2Cl_2 (50 mL) in a dry 250 mL Schlenk flask under nitrogen and placed in an ice bath. 3 drops of DMF were added followed by oxalyl chloride (2.8 mL, 33.0 mmol) which was added slowly over 2 min. The reaction was stirred at rt for 1 h, after which time solvent and residual oxalyl chloride were removed by vacuum. After drying for 1 h under vacuum, the crude acid chloride was dissolved in CH_2Cl_2 (40 mL) to which ferrocene (1.75 g, 9.5 mmol) was added followed by additional CH_2Cl_2 (40 mL). $AlCl_3$ (3.39 g, 25.3 mmol) was added in four portions and the reaction was refluxed for 19 h. The reaction was quenched with ice water (50 mL). The layers were separated, and the aqueous layers

was back extracted with CH_2Cl_2 (3 x 40 mL). The combined organic layers were washed with 2.0 M NaOH (2 x 40 mL), dried over $MgSO_4$, filtered and dried *in vacuo.* The red powder (3.9 g, 62%) was isolated without further purification. M.P.: 238-240°C (with degradation). ^{1}H NMR (500 MHz, $CDCl_3$) δ 7.08 (s, 1H), 7.00 (s, 1H), 4.85 (t, *J* = 2.0 Hz, 2H), 4.66 (t, *J* = 2.0 Hz, 2H), 3.94 (s, 3H), 3.92 (s, 3H). ^{13}C NMR (126 MHz, $CDCl_3$) δ 197.3, 150.8, 147.9, 132.6, 116.0, 112.0, 110.5, 80.2, 74.3, 72.8, 56.28, 56.27. IR (ATR) ν_{max} 3663, 2973, 14647, 1271, 1080 cm^{-1}. HRMS: (ESI-TOF) calculated for $C_{28}H_{24}Br_2FeO_6$ $[M+H]^+$ 670.9367, found 670.9366.

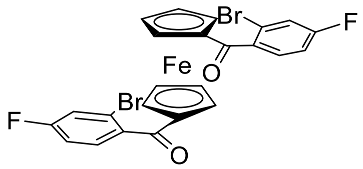

1,1'-Di(2-bromo-6-fluorobenzoyl)ferrocene 12e: 2-Bromo-6-fluorobenzoic acid (4.00 g, 18.2 mmol) was dissolved in CH_2Cl_2 (30 mL) in a dry 250 mL Schlenk flask under nitrogen and placed in an ice bath. 3 drops of DMF were added followed by oxalyl chloride (1.9 mL, 21.9 mmol) which was added slowly over 2 min. The reaction was stirred at rt for 1 h, after which time solvent and residual oxalyl chloride were removed by vacuum. After drying for 1 h under vacuum, the crude acid chloride was dissolved in CH_2Cl_2 (30 mL) to which ferrocene (1.35 g, 7.3 mmol) was added followed by additional CH_2Cl_2 (30 mL). $AlCl_3$ (2.43 g, 18.2 mmol) was added in four portions and the reaction was refluxed for 19 h. The reaction was quenched with ice water (50 mL). The layers were separated, and the aqueous layers was back-extracted with CH_2Cl_2 (3 x 40 mL). The combined organic layers were washed with 2.0 M NaOH (2 x 40 mL), dried over $MgSO_4$, filtered and dried *in vacuo.* The red powder (4.30 g, 100%) was isolated without further purification. M.P.: 138-144 °C. ^{1}H NMR (300 MHz, $CDCl_3$) δ 7.50 (dd, *J* = 8.6, 5.8 Hz, 1H), 7.36 (dd, *J* = 8.3, 2.4 Hz, 1H), 7.11 (td, *J* = 8.2, 2.5 Hz, 1H), 4.82 (t, *J* = 1.9 Hz, 1H), 4.71 (t, *J* = 2.0 Hz, 1H).). ^{19}F NMR (282 MHz, $CDCl_3$) δ -107.23 ^{13}C NMR (126 MHz, $CDCl_3$) δ 197.1, 163.0 (d, *J* = 255.1 Hz), 136.6, 130.6 (d, *J* = 8.4 Hz), 121.2 (d, *J* = 24.4 Hz), 120.6 (d, *J* = 9.5 Hz), 114.4 (d, *J* = 21.0 Hz), 79.7, 74.8,

72.7. IR (ATR) ν_{max} 3060, 1637, 827, 819 cm^{-1}. HRMS: (ESI-TOF) calculated for $C_{24}H_{14}Br_2F_2FeO_2$ $[M+H]^+$ 586.8756, found 586.8759.

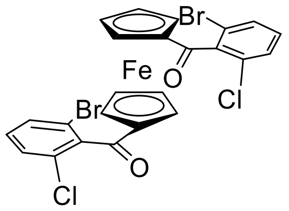

1,1’-Di(2-chloro-6-bromobenzoyl)ferrocene 12f: 2-Chloro-6-bromobenzoic acid (6.00 g, 25.3 mmol) was dissolved in CH_2Cl_2 (50 mL) in a dry 250 mL Schlenk flask under nitrogen and placed in an ice bath. 3 drops of DMF were added followed by oxalyl chloride (2.8 mL, 33.0 mmol) which was added slowly over 2 min. The reaction was stirred at rt for 1 h, after which time solvent and residual oxalyl chloride were removed by vacuum. After drying for 1 h under vacuum, the crude acid chloride was dissolved in CH_2Cl_2 (40 mL) to which ferrocene (1.75 g, 9.5 mmol) was added followed by additional CH_2Cl_2 (40 mL). $AlCl_3$ (3.39 g, 25.3 mmol) was added in four portions and the reaction was refluxed for 19 h. The reaction was quenched with ice water (100 mL). The layers were separated, and the aqueous layers was back-extracted with CH_2Cl_2 (3 x 50 mL). The combined organic layers were washed with 2.0 M NaOH (3 x 40 mL), dried over $MgSO_4$, filtered and dried *in vacuo.* The resulting powder was recrystallised from hot CH_2Cl_2 overnight in a freezer at -20 ^{o} C, affording the product as dark purple crystals (1.55 g, 97%). M.P.: 231-233 ^oC. ^1H NMR (300 MHz, $CDCl_3$) δ 7.51 (dd, *J* = 8.0, 1.1 Hz, 2H), 7.39 (dd, *J* = 8., 1.1 Hz, 2H), 7.23 (t, *J* = 8. Hz, 2H), 4.85-4.79 (m, 6H), 4.72-4.69 (m, 2H). ^{13}C NMR (101 MHz, $CDCl_3$) δ 197.4, 139.5, 132.0, 131.7, 131.1, 129.1, 120.5, 80.3, 72.7, 71.8. IR (ATR) ν_{max} 3674, 2981, 1646, 1293, 1080 cm^{-1}. HRMS: (ESI-TOF) calculated for $C_{24}H_{14}Br_2Cl_2FeO_2$ $[M+Na]^+$ 640.7985, found 640.7982.

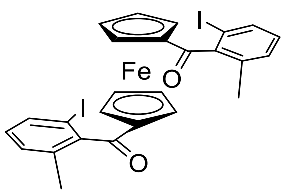

1,1'-Di(2-bromo-6-methylbenzoyl)ferrocene 12c: 2-Bromo-6-methylbenzoic acid (1.00 g, 3.8 mmol) was dissolved in CH_2Cl_2 (20 mL) in a dry 250 mL Schlenk flask under nitrogen and placed in an ice bath. 3 drops of DMF were added followed by oxalyl chloride (0.9 mL, 4.7 mmol) which was added slowly over 2 min. The reaction was stirred at rt for 1 h, after which time solvent and residual oxalyl chloride were removed by vacuum. After drying for 1 h under vacuum, the crude acid chloride was dissolved in CH_2Cl_2 (20 mL) to which ferrocene (0.28 g, 1.5 mmol) was added followed by additional CH_2Cl_2 (20 mL). $AlCl_3$ (1.03 g, 4.59 mmol) was added in four portions and the reaction was refluxed for 19 h. The reaction was quenched with ice water (50 mL). The layers were separated, and the aqueous layers was back-extracted with CH_2Cl_2 (3 x 20 mL). The combined organic layers were washed with 2.0 M NaOH (2 x 20 mL), dried over $MgSO_4$, filtered and dried *in vacuo.* The resulting powder was recrystallised from hot CH_2Cl_2 overnight in a freezer at -20 ^oC, affording the product as dark purple crystals (1.28 g, 84%). M.P.: 127-129 ^oC. ^1H NMR (500 MHz, $CDCl_3$) δ 7.68 (d, J = 7.8 Hz, 2H), 7.20 (d, J = 7.8 Hz, 2H), 7.00 (t, J = 7.8 Hz, 2H), 4.79 (s, 2H), 2.29 (s, 2H). ^{13}C NMR (126 MHz, $CDCl_3$) δ 202.7, 144.2, 137.2, 136.6, 130.5, 130.4, 120.8, 92.8, 81.1, 74.4, 20.7. IR (ATR) ν_{max} 2890, 1641, 1438, 1267, 676 cm^{-1}. HRMS: (ESI-TOF) calculated for $C_{26}H_{20}FeI_2O_2$ $[M+H]^+$ 674.8980, found 674.8975.

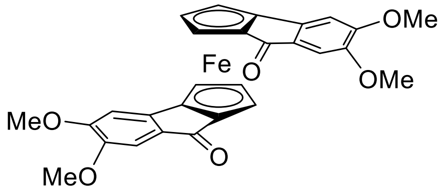

(S_p)-Dimethoxy-diketone – 8d: 1,1'-Di(2-bromo-4,5-dimethoxybenzoyl)ferrocene **12d** (1.50 g, 2.6 mmol) was added to a dry Schlenk flask under nitrogen. Cs_2CO_3 (3.32 g, 10.2 mmol), $Pd(OAc)_2$ (11.7 mg, 0.05 mmol, 2 mol%), (*S*)-BINAP (64.8 mg, 0.10 mmol, 4 mol%), and pivalic acid (102 mg, 0.78 mmol) were added sequentially.

Toluene (26 mL) was added and the reaction was stirred 100 °C for 48 h. The reaction was cooled to room temperature then filtered through a pad of Celite 545® using CH_2Cl_2. The solution was concentrated *in vacuo* to afford the di-cyclised product as dark purple crystals (412 mg, 64%, 99% ee, >99% de). SFC analysis (CHIRALPAK IC, $scCO_2$/iPrOH, 99/5 to 60/40 over 10 min, 2 mL/min) t_R = 6.05 min (S_p) and t_R = 6.57 min (R_p). M.P.: 290-283. $[\alpha]^{20}_D$ = -4960.8 (c = 0.0125 CH_2Cl_2), ^{1}H NMR (500 MHz, $CDCl_3$) δ 6.47 (s, 2H), 6.46 (s, 2H), 4.74 (t, *J* = 2.4 Hz, 2H), 4.69 (dd, *J* = 2.3, 0.7 Hz, 2H), 4.57 (dd, *J* = 2.4, 0.7 Hz, 2H), 3.97 (s, 6H), 3.81 (s, 6H). ^{13}C NMR (126 MHz, $CDCl_3$) δ 192.5, 153.5, 148.5, 132.6, 131.8, 105.8, 105.0, 95.3, 82.8, 76.8, 66.9, 65.8, 56.4, 55.7. IR (ATR) ν_{max} 33096, 1686, 1584, 775, 713 cm^{-1}. HRMS: (ESI-TOF) calculated for $C_{19}H_{17}OFe$ $[M+Na]^+$ calculated 533.0664, found 533.0657.

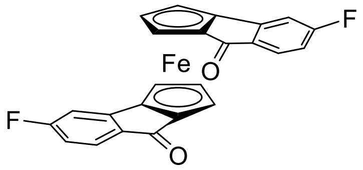

(S_p)-*p*-Fluoro-diketone – 8e: 1,1′-Di(2-bromo-4-fluorobenzoyl)ferrocene **12e** (1.50 g, 2.6 mmol, 1 eq) was added to a dry Schlenk flask under nitrogen. Cs_2CO_3 (3.32 g, 10.2 mmol, 4 eq), $Pd(OAc)_2$ (11.7 mg, 0.05 mmol, 2 mol%), (*S*)-BINAP (64.8 mg, 0.10 mmol, 4 mol%), and pivalic acid (102 mg, 0.78 mmol, 0.3 eq) were added sequentially. Toluene (26 mL) was added and the reaction was stirred 100 °C for 48 h. The reaction was cooled to room temperature then filtered through a pad of Celite 545® using CH_2Cl_2. The solution was concentrated *in vacuo* to afford the di-cyclised product as dark purple crystals (900 mg, 81%, 99% ee, >99% de). SFC analysis (CHIRALPAK IC, $scCO_2$/iPrOH, 99/1 to 60/40 over 7 min, 3 mL/min) t_R = 6.05 min (S_p) and t_R = 6.57 min (R_p). M.P.: 270-275 °C (with degradation). $[\alpha]^{20}_D$ = -1838.3 (c = 0.05 CH_2Cl_2); ^{1}H NMR (300 MHz, $CDCl_3$) δ 7.09 (t, *J* = 7.6 Hz, 1H), 6.74 (d, *J* = 7.6 Hz, 1H), 6.73 (d, *J* = 7.6 Hz, 1H), 4.72-4.68 (m, 2H), 4.68 – 4.65 (m, 1H). ^{19}F NMR (282 MHz, $CDCl_3$) δ -104.09 ^{13}C NMR (101 MHz, $CDCl_3$) δ 191.61, 166. (d, *J* = 254.6 Hz), 142.5 (d, *J* = 10.5 Hz), 135.2 (d, *J* = 3.0 Hz), 125.2 (d, *J* = 10.4 Hz), 113.4 (d, *J* = 23.3 Hz), 110.0 (d, *J* = 25.1 Hz), 93.3

(d, *J* = 2.6 Hz), 82.0, 77.7, 69.8, 66.9. IR (ATR) ν_{max} 3096, 1688, 1125, 1179, 492 cm^{-1}. HRMS: (ESI-TOF) calculated for $C_{24}H_{12}F_2FeO_2$ $[M+H]^+$ 427.0233, found 427.0227.

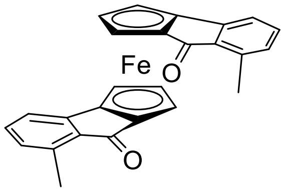

(*S_p*)-*o*-Methyl-diketone 8c: 1,1'-Di(2-bromo-6-methylbenzoyl)ferrocene **12c** (646 mg, 0.96 mmol) was added to a dry Schlenk flask under nitrogen. Cs_2CO_3 (1.35 g, 4.16 mmol), $Pd(OAc)_2$ (4.3 mg, 0.019 mmol, 2 mol%), (*S*)-BINAP (23.6 mg, 0.038 mmol, 4 mol%), and pivalic acid (29 mg, 0.288 mmol) were added sequentially. Toluene (10 mL) was added and the reaction was stirred 100 °C for 48 h. The reaction was cooled to room temperature then filtered through a pad of Celite 545® using CH_2Cl_2. The solution was concentrated *in vacuo* to afford the di-cyclised product as dark purple crystals (273 mg, 65%, 99% ee, >99% de). SFC analysis (CHIRALPAK ID, $scCO_2$/MeOH, 99/1 to 60/40 over 10 min, 3 mL/min) t_R = 7.13 min (S_p) and t_R = 7.54 min (R_p). M.P.: 249-251 °C. $[\alpha]^{20}_D$ = -4751.6 (c = 0.0125 CH_2Cl_2); ^{1}H NMR (500 MHz, $CDCl_3$) δ 7.09 (t, *J* = 7.6 Hz, 1H), 6.74 (d, *J* = 7.6 Hz, 1H), 6.73 (d, *J* = 7.6 Hz, 1H), 4.72-4.68 (m, 2H), 4.68 – 4.65 (m, 1H); ^{13}C NMR (126 MHz, $CDCl_3$) δ 194.0, 139.1, 138.4, 136.0, 132.9, 129.7, 119.3, 94.6, 82.4, 76.7, 66.9, 65.9, 17.8. IR (ATR) ν_{max} 2981, 1672, 831, 507 cm^{-1}. HRMS: (ESI-TOF) calculated for $C_{26}H_{19}FeO_2$ $[H+]^+$ 419.0734, found 419.0738.

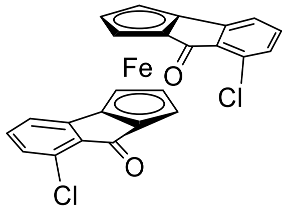

(*S_p*)-*o*-Chloro-diketone -8a: 1,1'-Di(2-chloro-6-bromobenzoyl)ferrocene **12a** (1.00 g, 1.6 mmol) was added to a dry Schlenk flask under nitrogen. Cs_2CO_3 (2.08 g, 6.4 mmol), $Pd(OAc)_2$ (7.2 mg, 0.032 mmol, 2 mol%), (*S*)-BINAP (38.6 mg, 0.064 mmol, 4 mol%), and pivalic acid (49 mg, 0.48 mmol) were added sequentially. Toluene (16 mL) was added and the reaction was stirred 100 °C for 48 h. The reaction was cooled to room

temperature then filtered through a pad of Celite 545® using CH_2Cl_2. The solution was concentrated *in vacuo* to afford the di-cyclised product as dark purple crystals (732 mg, 93%, 99% ee, >99% de). SFC analysis (CHIRALPAK IB, $scCO_2$/*i*PrOH, 99/1 to 60/40 over 7 min, 3 mL/min) t_R = 5.96 min (S_p) and t_R = 7.14 min (R_p). M.P.: >300 °C. $[\alpha]^{20}_D$ = -3437.2 (c = 0.0125 CH_2Cl_2); ^{1}H NMR (500 MHz, $CDCl_3$) δ 7.21 (d, *J* = 0.8 Hz, 1H), 6.95 (dd, *J* = 8.2, 0.9 Hz, 1H), 6.80 (dd, *J* = 7.4, 0.9 Hz, 1H), 4.82 (t, *J* = 2.4 Hz, 1H), 4.76 (d, *J* = 2.5 Hz, 2H). ^{13}C NMR (101 MHz, $CDCl_3$) δ 189.7, 140.3, 134.7, 133.8, 131.3, 129.0, 120.1, 93.2, 82.5, 76.8, 67.6, 66.5. IR (ATR) ν_{max} 3089, 1685, 713 cm^{-1}. HRMS: (ESI-TOF) calculated for $C_{24}H_{12}Cl_2FeO_2$ $[M+H]^+$ 458.9642, found 458.9639.

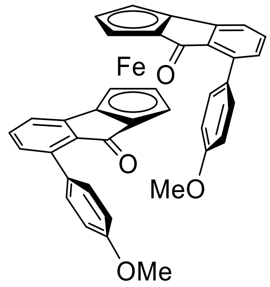

(S_p)- *o*-(4-Methoxyphenyl)-diketone 8f: *o*-Chloro-diketone – **8a** (300 mg, 0.65 mmol), $Pd_2(dba)_3$ (18 mg, 0.02 mmol, 13 mol%), Xphos (23 mg, 0.05 mmol, 7.2 mol%), 4-methoxyphenyl boronic acid (646 mg, 4.25 mmol), and Cs_2CO_3 (1.53 g, 4.7 mmol) were combined in a 25 mL Schlenk under nitrogen. 1,4-Dioxane (6 mL) was added, and the reaction was stirred at room temperature for 16 h. The reaction mixture was cooled to room temperature and filtered through a pad of Celite with Et_2O and concentrated. The resulting red oil was purified through a silica plug (30% EtOAc/cHex), to afford the desired cross coupled product as a fine red powder (387 mg, 99% yield). M.P.: 240-242 °C. $[\alpha]^{20}_D$ = -5155.0 (c = 0.013 CH_2Cl_2); ^{1}H NMR (500 MHz, $CDCl_3$) δ 7.41 (d, *J* = 8.3 Hz, 2H), 7.07 (t, *J* = 7.4 Hz, 1H), 6.98 (d, *J* = 8.3 Hz, 2H), 6.95 (d, *J* = 7.4 Hz, 1H) 6.60 (d, *J* = 7.4 Hz, 1H), 4.80 (t, *J* = 2.7 Hz, 1H), 4.68 (m, 2H), 3.89 (s, 3H). ^{13}C NMR (126 MHz, $CDCl_3$) δ 193.2, 159.6, 141.1, 140.9, 134.5, 133.4, 130.5, 129.8, 129.7, 119.9, 113.2, 94.3, 81.7, 69.4, 66.4, 55.3. IR (ATR) ν_{max} 3670, 2993, 1686, 1049 cm^{-1}. HRMS: (ESI-TOF) calculated for $C_{38}H_{26}FeO4$ $[M+H]^+$ 603.1258, found 603.1245.

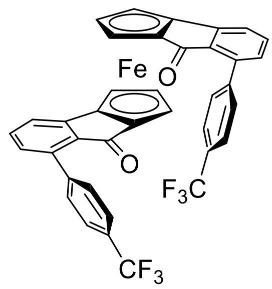

(S_p)- *o*-(4-Trifluoromethylphenyl)-diketone 8g: Procedure A: 2-Chloro-diketone –**8a** (300 mg, 0.65 mmol), $Pd_2(dba)_3$ (18 mg, 0.02 mmol, 13 mol%), Xphos (23 mg, 0.05 mmol, 7.2 mol%), 4-trifluoromethylphenyl boronic acid (807 mg, 4.25 mmol), and Cs_2CO_3 (1534 mg, 4.7 mmol) were combined in a 25 mL Schlenk under nitrogen. 1,4-Dioxane (6 mL) was added, and the reaction was stirred at room temperature for 70 h. The reaction mixture was cooled to room temperature and filtered through a pad of Celite with Et_2O and concentrated. The resulting red oil was purified through a silica plug (40% EtOAc/cHex), to afford the desired cross coupled product as a fine red powder (287 mg, 98%). M.P.: 239-242 °C. $[\alpha]^{20}_{D}$ = -2220.6 (c = 0.025 CH_2Cl_2); ^{1}H NMR (400 MHz, $CDCl_3$) δ 7.66 (d, *J* = 7.6 Hz, 4H), 7.50 (d, *J* = 7.6 Hz, 4H), 7.15 (t, *J* = 7.7 Hz, 2H), 6.94 (dd, *J* = 7.7, 1.0 Hz, 2H), 6.74 (dd, *J* = 7.7, 1.0 Hz, 2H), 4.83 (t, *J* = 2.4 Hz, 4H), 4.69 (dd, *J* = 2.4, 0.7 Hz, 2H), 4.68 (dd, *J* = 2.4, 0.7 Hz, 2H. ^{19}F NMR (282 MHz, $CDCl_3$) δ -62.51. ^{13}C NMR (101 MHz, $CDCl_3$) δ 192.5, 141.3, 141.0, 141.0, 139.6, 134.9, 133.6, 130.1, 129.8, 129.6, 124.7 (q, *J* = 4.0 Hz), 120.9, 93.8, 81.7, 77.5, 68.9, 67.1. IR (ATR) ν_{max} 3734, 2987, 1692, 1323, 1056 cm^{-1}. HRMS: (ESI-TOF) calculated for $C_{38}H_{20}F_6FeO_2$ $[M+H]^+$ 679.0795, found 679.0791.

Procedure B: 2-Chloro-diketone **8a** (300 mg, 0.65 mmol), PEPPSI-*i*Pr (18 mg, 0.026 mmol, 4 mol%) 4-trifluoromethylphenyl boronic acid (296 mg, 1.56 mmol), and KOH (218mg, 3.9 mmol) were combined in a 25 mL Schlenk under nitrogen. 1,4-Dioxane (6 mL) was added, and the reaction was stirred at room temperature for 1 h. The reaction mixture was cooled to room temperature and filtered through a pad of Celite with Et_2O and concentrated. The resulting red oil was purified through a silica plug (40% EtOAc/cHex), to afford the desired cross coupled product as a fine red

powder (277 mg, 86%). Identical in all respects to the sample previously prepared by Procedure A.

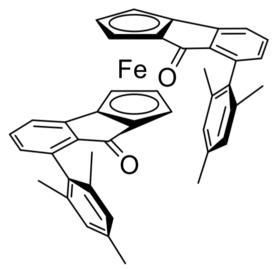

(S_p)- *o*-Mesityl-diketone 8i: 2-Chloro-diketone **8a** (300 mg, 0.65 mmol, 1 eq), PEPPSI-*i*Pr (18 mg, 0.026 mmol, 4 mol%) 2,4,6-trimethylphenyl boronic acid (256 mg, 1.56 mmol, 2.4 eq), and KOH (218mg, 3.9 mmol, 6 eq) were combined in a 25 mL Schlenk under nitrogen. 1,4-dioxane (6 mL) was added, and the reaction was stirred at room temperature for 70 h. The reaction mixture was cooled to room temperature and filtered through a pad of Celite with Et_2O and concentrated. The resulting red oil was purified through a silica plug (40% EtOAc/cHex), to afford the desired cross coupled product as a fine red powder (251 mg, 86%). M.P.: 245-247 °C. $[\alpha]^{20}_D$ = -1450.3 (c = 0.05 CH_2Cl_2);

^{1}H NMR (400 MHz, $CDCl_3$) δ 7.27 (t, *J* = 7.6 Hz, 2H), 7.01 (s, 2H), 6.94 (s, 2H), 6.88 (dd, *J* = 7.8, 1.0 Hz, 2H), 6.62 (dd, *J* = 7.4, 1.0 Hz, 2H), 4.81 (t, *J* = 2.4 Hz, 2H), 4.66 (dd, *J* = 2.4, 0.7 Hz, 2H), 4.60 (dd, *J* = 2.4, 0.7 Hz, 2H), 2.36 (s, 6H), 2.27 (s, 6H), 1.87 (s, 6H). ^{13}C NMR (101 MHz, $CDCl_3$) δ 193.1, 141.3, 139.7, 136.8, 136.4, 135.2, 134.9, 134.2, 133.5, 129.8, 128.2, 128.0, 120.7, 94.6, 80.0, 77.9, 71.1, 67.1, 21.2, 20.9, 20.3. IR (ATR) ν_{max} 3674, 2987, 1695, 1066 cm^{-1}.HRMS: (ESI-TOF) calculated for $C_{42}H_{34}FeO_2$ $[M+H]^+$ 627.1986, found 627.1974.

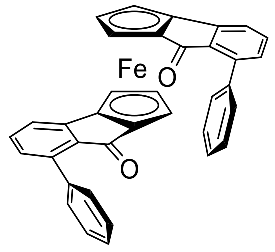

(S_p)- *o*-phenyl-diketone 8h: Procedure A: 2-Chloro-diketone **8a** (300 mg, 0.65 mmol), $Pd_2(dba)_3$ (18 mg, 0.02 mmol, 13 mol%), Xphos (23 mg, 0.05 mmol, 7.2 mol%), phenyl boronic acid (646 mg, 3.9 mmol), and Cs_2CO_3 (1534 mg, 4.7 mmol) were combined in a 25 mL Schlenk under nitrogen. 1,4-dioxane (6 mL) was added, and the reaction was stirred at room temperature for 46 h. The reaction mixture was cooled to room temperature and filtered through a pad of Celite with Et_2O and concentrated. The resulting red oil was purified through a silica plug (30% EtOAc/cHex), to afford the desired cross coupled product as a fine red powder (287 mg, 98%). M.P.: 270-272 ^{o}C. $[\alpha]^{20}_{D}$ = -4880.5 (c = 0.013 CH_2Cl_2); ^{1}H NMR (400 MHz, $CDCl_3$) δ 7.49-7.39 (m, 10H), 7.07 (t, *J* = 7.6 Hz, 2H), 6.96 (d, *J* = 7.6 Hz, 2H), 6.60 (d, *J* = 7.6 Hz, 2H), 4.79 (t, *J* = 2.5 Hz, 2H), 4.67 (d, *J* = 2.5 Hz, 4H). ^{13}C NMR (126 MHz, $CDCl_3$) δ 193.0, 141.2 141.2, 137.5, 134.8, 133.5, 129.8, 129.2, 128.0, 127.7, 120.3, 94.3, 81.6, 69.6, 66.4. IR (ATR) ν_{max} 3675, 2987, 1689, 757 cm^{-1}. HRMS: (ESI-TOF) calculated for $C_{36}H_{22}FeO_2$ $[M+H]^+$ 543.1047, found 543.1052.

Procedure B: 2-Chloro-diketone **8a** (300 mg, 0.65 mmol), PEPPSI-*i*Pr (18 mg, 0.026 mmol, 4 mol%) phenyl boronic acid (190 mg, 1.56 mmol), and KOH (218 mg, 3.9 mmol) were combined in a 25 mL Schlenk under nitrogen. 1,4-Dioxane (6 mL) was added, and the reaction was stirred at room temperature for 1 h. The reaction mixture was cooled to room temperature and filtered through a pad of Celite with Et_2O and concentrated. The resulting red oil was purified through a silica plug (40% EtOAc/cHex), to afford the desired cross coupled product as a fine red powder (289 mg, 98%). Identical in all respects to the sample previously prepared by Procedure A.

General procedure for the synthesis of bis-trifluoromethyl diols 14

Diketone (200 mg, 0.33 mmol) and $TMSCF_3$ (0.25 mL, 1.65 mmol) were added to a dry 25 mL 2-neck round bottom under nitrogen. THF (3 mL) was added, and the resulting red solution was cooled in an ice bath with stirring. TBAF (0.1 mL, 1.0 M solution in THF, 0.1 mmol) was added and the reaction mixture was stirred until complete by TLC analysis at 0 ^{o}C. The remaining TBAF (1.6 mL, 1.0 M solution in THF,

1.6 mmol) was added slowly at 0 °C and the reaction was warmed to room temperature over 10 min. The reaction was quenched with H_2O (10 mL), the organic and aqueous layers were separated and the aqueous layer was back-extracted with Et_2O (3 x 30 mL). The combined organic layers were dried over Na_2SO_4, filtered and concentrated. The resulting oil was purified by SiO_2 chromatography affording the desired product.

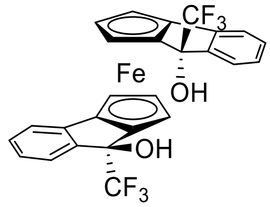

(*Sp*)- CF_3-diol 14:** Dark orange solid (170 mg, 97%). ^{1}H NMR (300 MHz, $CDCl_3$) δ 7.49 – 7.40 (m, 2H), 7.34 – 7.12 (m, 6H), 4.76 – 4.69 (m, 2H), 4.45 – 4.37 (m, 2H), 4.21 (t, *J* = 2.4 Hz, 2H), 4.15 (s, 2H); ^{13}C NMR (101 MHz, $CDCl_3$) δ 145.0, 139.2, 130.4, 126.6, 125.1, 124.5 (q, *J* = 284 Hz), 121.1, 92.9, 92.9, 91.4, 73.9, 67.4, 62.9; ^{19}F NMR (282 MHz, $CDCl_3$) δ - 79.4. All physical data were identical to those previously reported.[2]

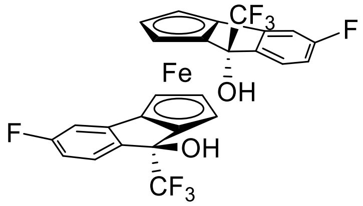

(S_p)- *p*-Fluoro-CF_3-diol 14e: Fine orange needles (132 mg, 71%). Crystals suitable for X-ray analysis were grown by dissolving **14e** in CH_2Cl_2 (1 mL), and carefully layering with pentane (0.5 mL) followed by slow evaporation of the solvents at room temperature. M.P.: 196-200 °C. $[\alpha]^{20}_D$ = -1537.5 (c = 0.044 CH_2Cl_2); ^{1}H NMR (400 MHz, cdcl$_3$) δ 7.40 (dd, *J* = 8.5, 4.9 Hz, 1H), 6.89 (ddd, *J* = 8.5, 8.4, 2.4 Hz, 1H), 6.75 (dd, *J* = 8.5, 2.4 Hz, 1H), 4.75 (dd, *J* = 2.5, 0.7 Hz, 1H), 4.60 (s, 1H), 4.41 (d, *J* = 2.5 Hz, 1H), 4.23 (t, *J* = 2.4 Hz, 1H. ^{19}F NMR (282 MHz, $CDCl_3$) δ-111.13, -79.46. ^{13}C NMR (126 MHz, $CDCl_3$) δ 164.3 (d, *J* = 248.4 Hz), 142.0, 139.7, 126.1 (d, *J* = 9.6 Hz), 124.3 (q, *J* = 284.8 Hz), 113.0 (d, *J* = 23.0 Hz), 109.1 (d, *J* = 24.3 Hz), 93.8, 90.1, 77.9 (q, *J* = 31.4 Hz), 73.9, 67.3, 63.0. IR (ATR) ν_{max} 3328, 1176,

816, 510 cm^{-1}. HRMS: (ESI-TOF) calculated for $C_{26}H_{14}O_2FeF_8$ $[M]^+$ 566.0215, found 566.0219.

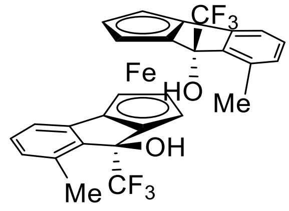

(S_p)- *o*-Methyl-CF_3-diol 14c: Dark orange solid (127 mg, 69%). Crystals suitable for X-ray analysis were grown by dissolving **14c** in CH_2Cl_2 (1 mL), and carefully layering with pentane (0.5 mL) followed by slow evaporation of the solvents at room temperature.M.P.: 247-250 °C. $[\alpha]^{20}_D$ = -1537.5 (c = 0.05 CH_2Cl_2); ^1H NMR (500 MHz, $CDCl_3$) δ 7.17 (t, *J* = 7.5 Hz, 2H), 7.08 (d, *J* = 7.5 Hz, 2H), 6.98 (d, *J* = 7.5 Hz, 2H), 4.68 (d, *J* = 2.4 Hz, 2H), 4.53 (d, *J* = 2.4 Hz, 2H), 4.32 (t, *J* = 2.4 Hz, 2H), 2.85 (s, 2H), 2.32 (s, 6H). ^{19}F NMR (282 MHz, $CDCl_3$) δ-76.99; ^{13}C NMR (126 MHz, $CDCl_3$) δ 141.7, 139.0, 137.8, 130.3, 130.0, 124.3 (q, *J* = 285.4 Hz), 118.2, 92.5 (q, *J* = 1.9 Hz), 91.8, 81.1 (q, *J* = 31.1 Hz), 72.5, 64.6, 61.5, 17.9 (q, *J* = 3.6 Hz). IR (ATR) ν_{max} 3571, 2922, 1224, 1158 cm^{-1}. HRMS: (ESI-TOF) calculated for $C_{28}H_{20}O_2FeF_6$ $[M]^+$ 558.0717, found 558.0719.

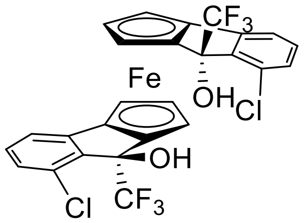

(S_p)- *o*-Chloro-CF_3-diol 14a: Fine orange needles (135 mg, 68%). Crystals suitable for X-ray analysis were grown by dissolving **14a** in CH_2Cl_2 (1 mL), and carefully layering with pentane (0.5 mL) followed by slow evaporation of the solvents at room temperature. M.P.: 188-194 °C (with degradation). $[\alpha]^{20}_D$ = -1639.0 (c = 0.05 CH_2Cl_2); ^1H NMR (300 MHz, $CDCl_3$) δ 7.08 (d, *J* = 1.5 Hz, 2H), 7.07 (m, 2H), 6.89 (dd, *J* = 4.9, 3.6 Hz, 2H), 4.78 (dd, *J* = 2.4, 0.8 Hz, 2H), 4.49 (d, *J* = 2.4 Hz, 2H), 4.41 (s, 2H), 4.25 (d, *J* = 2.4 Hz, 2H). ^{19}F NMR (282 MHz, $CDCl_3$) δ -76.39. ^{13}C NMR (101 MHz, $CDCl_3$) δ 141.3, 139.3, 131.4, 130.8, 127.3, 124.14 (q, *J* = 286.9 Hz), 119.8, 92.1, 90.8, 80.4 (d, *J* = 31.9 Hz),

73.7, 65.9, 63.3. IR (ATR) ν_{max} 3514, 1267, 1158, 790 cm^{-1}. HRMS: (ESI-TOF) calculated for $C_{26}H_{14}O_2FeCl_2F_6$ $[M]^+$ 597.9646, found 597.9624.

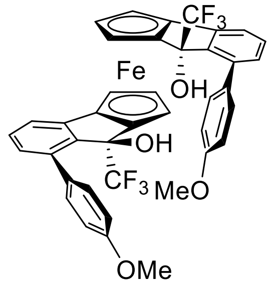

(S_p)- *o*-(4-Methoxyphenyl)-CF$_3$-diol 14f: Fine orange powder (193 mg, 79%). Crystals suitable for X-ray analysis were grown by dissolving **14f** in CH_2Cl_2 (1 mL), and carefully layering with pentane (0.5 mL) followed by slow evaporation of the solvents at room temperature. M.P.: 216-220 °C. $[\alpha]^{20}_D$ = -1433.4 (c = 0.05 CH_2Cl_2); ^1H NMR (500 MHz, $CDCl_3$) δ 7.39 (d, *J* = 8.6 Hz, 4H), 7.25 (t, *J* = 7.5 Hz, 2H), 7.20 (dd, *J* = 7.5, 1.3 Hz, 2H), 7.01 (dd, *J* = 7.6, 1.3 Hz, 2H), 6.98 (d, *J* = 8.6 Hz, 4H), 4.84 (d, *J* = 2.3 Hz, 2H), 4.60 (s, 2H), 4.40 (d, *J* = 2.3 Hz, 2H), 4.11 (t, *J* = 2.4 Hz, 6H), 3.88 (s, 3H). ^{19}F NMR (282 MHz, $CDCl_3$) δ -76.89. ^{13}C NMR (126 MHz, $CDCl_3$) δ 159.1, 141.6, 141.2, 140.2, 132.4, 130.4, 129.7, 124.3 (q, *J* = 286.8 Hz), 119.8, 113.5, 92.8, 90.3, 80.5 (q, *J* = 31.6 Hz), 74.1, 67.6, 62.9, 55.3. IR (ATR) ν_{max} 3529, 3482, 2917, 1149, 804 cm^{-1}. HRMS: (ESI-TOF) calculated for $C_{19}H_{17}OFe$ $[M+Na]^+$ 765.1147, found 765.1139.

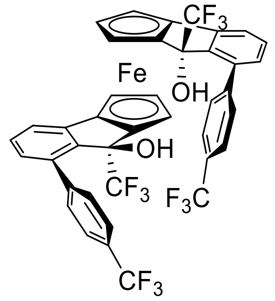

(S_p)- *o*-(4-Trifluromethylphenyl)-CF$_3$-diol 14g: Orange powder (207 mg, 77%). M.P.: 218-233 °C (with degradation). $[\alpha]^{20}_D$ = -1836 (c = 0.25, CH_2Cl_2); ^1H NMR (500 MHz, $CDCl_3$) δ 7.70 (d, *J* = 8.0 Hz, 4H), 7.57 (d, *J* = 8.0 Hz, 4H), 7.39 – 7.27 (m, 4H), 7.00 (dd, *J* = 7.2, 1.6 Hz, 2H), 4.88 (d, *J* = 2.3 Hz, 2H), 4.44 (d, *J* = 2.3 Hz, 2H), 4.37 (s, 2H), 4.17 (t, *J* = 2.3 Hz, 2H).^{19}F NMR (470 MHz, $CDCl_3$) δ -62.52, -76.89.^{13}C NMR (126 MHz,

$CDCl_3$) δ 143.8, 141.0, 140.5, 137.9, 130.1, 129.9 (q, *J* = 32.5 Hz), 129.8, 129.1, 128.2, 127.8–122.2 (m)*, 120.6, 92.9, 90.2, 80.5, 74.3, 67.5, 63.0 (d, *J* = 4.4 Hz), 26.9, 21.5. *Overlap of signals from CF_3 carbon signals prevents detection of coupling constants. IR (ATR) ν_{max} 2980, 2897, 1311, 1108, 1073 cm^{-1}. HRMS: (ESI-TOF) calculated for $C_{40}H_{22}F_{12}FeO_2$ $[M]^+$ 818.0778, found 818.0772.

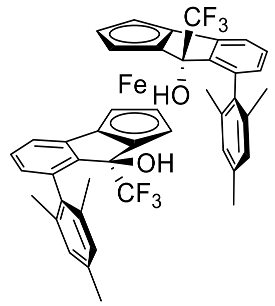

(S_p)- *o*-Mesityl-CF_3-diol 14i: Orange powder (146 mg, 58%). M.P.: 235-237 °C. $[\alpha]^{20}_D$ = -1203 (c = 0.05 CH_2Cl_2); ^{1}H NMR (400 MHz, $CDCl_3$) δ 7.33 (t, *J* = 7.5 Hz, 2H), 7.27 (d, *J* = 1.3 Hz, 2H), 7.25 (d, *J* = 1.3 Hz, 2H), 6.93 (s, 2H), 6.92 (s, 2H), 6.87 (dd, *J* = 7.5, 1.3 Hz, 12), 4.81 (d, *J* = 2.4 Hz, 2H), 4.62 (s, 2H), 4.23 (d, *J* = 2.4 Hz, 2H), 4.11 (t, *J* = 2.4 Hz, 2H), 2.33 (s, 6H), 2.14 (s, 6H), 1.94 (s, 6H). ^{19}F NMR (282 MHz, $CDCl_3$) δ -69.88. ^{13}C NMR (101 MHz, $cdcl_3$) δ 141.4, 140.7, 139.9, 137.4, 137.3, 135.4, 135.3, 130.3, 129.1, 128.3, 127.9, 124.0 (q, *J* = 286.2 Hz), 120.0, 92.4 (q, *J* = 1.9 Hz), 90.5, 80.1 (q, *J* = 31.3 Hz), 74.0, 67.7, 62.9, 21.1, 20.9, 20.5. IR (ATR) ν_{max} 3665, 3417, 2914, 1266, 1167 cm^{-1}. HRMS: (ESI-TOF) calculated for $C_{44}H_{33}F_6FeO_2$ $[M]^+$ 766.1959, found 766.1959.

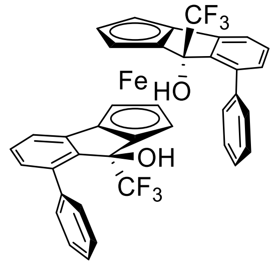

(S_p)- *o*-Phenyl-CF_3-diol 14h: Orange solid (171 mg, 76%). M.P.: 189-190 °C. $[\alpha]^{20}_D$ = -1324 (c = 0.05 CH_2Cl_2); ^{1}H NMR (300 MHz, $CDCl_3$) δ 7.50 – 7.38 (m, 10H), 7.34 – 7.18 (m, 4H), 7.03 (dd, *J* = 7.3, 1.5 Hz, 2H), 4.86 (d, *J* = 2.4, 2H), 4.54 (s, 2H), 4.43 (d, *J* = 2.4 Hz, 2H), 4.13 (t, *J* = 2.4 Hz, 2H). ^{19}F NMR (282 MHz, $CDCl_3$) δ -76.93. ^{13}C NMR (101 MHz, $CDCl_3$) δ 141.8, 141.0, 140.2, 140.1, 129.7, 129.4, 129.2, 128.0, 127.6, 124.24

(q, *J* = 286.7 Hz), 120.0, 92.8, 90.3, 80.5 (q, *J* = 31.5 Hz), 74.1, 67.6, 62.9. IR (ATR) ν_{max} 3493, 3410, 2977, 1269, 1155 cm^{-1}. (ESI-TOF) calculated for $C_{38}H_{24}F_6FeO_2$ $[M]^+$ 682.1029, found 682.1026.

General procedure for the synthesis of bis-naphthyl diols:

1-Bromonaphthalene (0.28 mL, 2.0 mmol) was added to a dry, nitrogen flushed 50 mL Schlenk flask equipped with a magnetic stirring bar and dissolved in THF (5 mL). The flask was cooled to -78 ^{o}C in a liquid nitrogen/acetone bath and *n*BuLi (0.8 mL, 2.5 M in hexanes, 2.0 mmol) was added dropwise. After 10 min at -78 ^{o}C a solution of diketone (0.5 mmol) in THF (10 mL) was added and the reaction allowed to warm to room temperature over 5 min. The reaction was quenched with sat. aq. $NaHCO_3$ (10 mL), the organic and aqueous layers were separated and the aqueous layer was back-extracted with Et_2O (2 x 20 mL), the combined organic layers were dried (Na_2SO_4), filtered and concentrated in vacuo followed by silica gel chromatography (CH_2Cl_2).

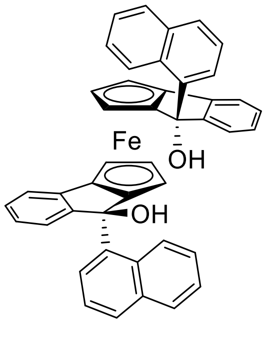

(*S*$_p$)- Naphthyl-diol 13: Orange solid (272 mg, 84%). ^{1}H NMR (400 MHz, $CDCl_3$) δ 7.83 – 7.76 (m, 2H), 7.72 – 7.64 (m, 2H), 7.51 – 7.39 (m, 4H), 7.25 – 7.10 (m, 14H), 4.69 – 4.63 (m, 2H), 4.44 – 4.39 (m, 2H), 4.29 (t, *J* = 2.4 Hz, 2H), 3.81 (s, 2H). TLC (CH_2Cl_2), R_f: 0.60 (UV, Vis, orange). All physical data was identical to those previously reported.[2]

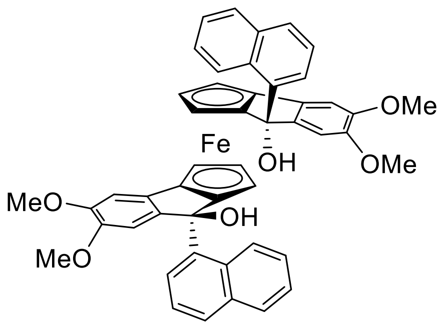

(S_p)- Dimethoxy-naphthyl-diol 13d: Orange solid (367 mg, 96%). M.P.: 189-192 °C (with degradation). $[\alpha]^{20}_{D}$ = -2013 (c = 0.05 CH_2Cl_2); ^{1}H NMR (500 MHz, $CDCl_3$) δ 9.18 (s, 2H), 7.83 (d, *J* = 8.2 Hz, 2H), 7.72 (d, *J* = 8.2 Hz, 2H), 7.66 – 7.39 (m, 4H), 7.18 (s, 2H), 6.93 - 6.73 (m, 6H), 4.76 (s, 2H), 4.45 (s, 2H), 4.28 (s, 2H), 3.92 (s, 6H), 3.83 (s, 6H), 3.29 (s, 2H). ^{13}C NMR (126 MHz, $CDCl_3$) δ 149.6, 148.7, 139.1, 134.8, 131.3, 129.0, 128.9, 128.2, 127.5, 125.4, 124.8, 124.2, 109.5, 104.3, 92.8, 70.8, 63.8, 59.9, 56.1, 56.0. IR (ATR) ν_{max} 3687, 2987, 1250, 1049 cm^{-1}. HRMS: (ESI-TOF) calculated for $C_{48}H_{38}O_6Fe$ $[M]^+$ 766.2018, found 766.2020.

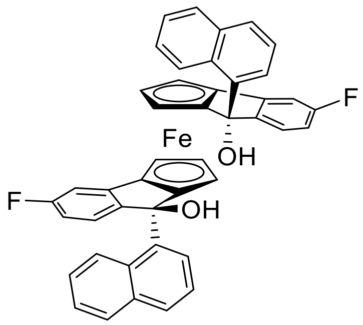

(S_p)- *p*-Fluoro-naphthyl-diol 13e: Orange powder (255 mg, 75%). Crystals suitable for X-ray analysis were grown by dissolving **13e** in CH_2Cl_2 (1 mL), and carefully layering with pentane (0.5 mL) followed by slow evaporation of the solvents at room temperature. M.P.: 188-194 °C (with degradation). $[\alpha]^{20}_{D}$ = -2048.1 (c = 0.025 CH_2Cl_2); ^{1}H NMR (300 MHz, $CDCl_3$) δ ^{1}H NMR (300 MHz, $CDCl_3$) δ 8.87 – 8.41 (m, 2H), 7.83 – 7.74 (m, 2H), 7.63 (d, *J* = 8.0 Hz, 2H), 7.46 (d, *J* = 6.7 Hz, 4H), 7.25 – 7.15 (m, 2H), 7.10 – 6.99 (m, 2H), 6.93 (t, *J* = 8.9 Hz, 2H), 6.78 (dd, *J* = 8.6, 2.4 Hz, 2H), 4.69 (d, *J* = 2.5 Hz, 2H), 4.35 (d, *J* = 2.5 Hz, 2H), 4.32 (t, *J* = 2.5 Hz, 2H).^{19}F NMR (282 MHz, $CDCl_3$) δ -114.19. ^{13}C NMR (126 MHz, $CDCl_3$) δ 163.42 (d, J = 245.8 Hz), 148.7, 139.3 (d, *J* = 116.2 Hz), 134.7, 130.9, 129.2, 129.0, 127.2, 126.7, 125.5, 125.3, 124.7, 124.1, 123.9, 112.8

(d, J = 22.8 Hz), 108.6 (d, J = 23.8 Hz), 90.1, 81.2, 72.0, 65.3, 60.9. IR (ATR) ν_{max} 3443, 2989, 1269, 1172 cm^{-1}. HRMS: (ESI-TOF) calculated for $C_{44}H_{28}O_2FeF_2$ $[M]^+$ 682.1407, found 682.1425.

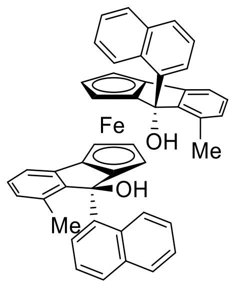

(S_p)- *o*-Methyl-naphthyl-diol 13c: Orange solid (273 mg, 81%). Crystals suitable for X-ray analysis were grown by dissolving **13c** in CH_2Cl_2 (1 mL), and carefully layering with pentane (0.5 mL) followed by slow evaporation of the solvents at room temperature. M.P.: 171-174 °C. $[\alpha]^{20}_D$ = -2107.3 (c = 0.,25 CH_2Cl_2); ^1H NMR (500 MHz, $CDCl_3$) δ 9.25 (d, J = 8.8 Hz, 2H), 7.85 (d, J = 8.2 Hz, 2H), 7.69 (d, J = 8.3 Hz, 4H), 7.53 (t, J = 7.5 Hz, 2H), 7.20-7.11 (m, 4H), 7.09 – 6.96 (m, 4H), 6.75 (d, J = 7.6 Hz, 2H), 4.76-4.71 (m, 2H), 4.59 – 4.47 (m, 2H), 4.22-4.18 (m, 2H), 3.07 (s, 2H), 2.15 (s, 6H).^{13}C NMR (126 MHz, $CDCl_3$) δ 149.5, 138.0, 137.4, 137.1, 135.1, 131.5, 129.4, 129.2, 129.0, 128.6, 127.7, 125.4, 125.2, 124.8, 124.5, 122.9, 118.0, 103.2, 91.6, 83.5, 71.3, 63.7, 60.5, 53.4. IR (ATR) ν_{max} 3675, 2987, 1047, 815 cm^{-1}. HRMS: (ESI-TOF) calculated for $C_{46}H_{34}O_2Fe$ $[M]^+$ 674.1908, found 674.1908.

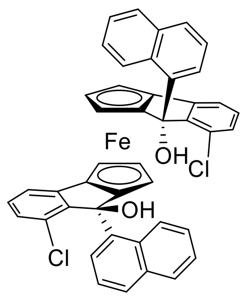

(S_p)- *o*-Chloro-naphthyl-diol 13a: Orange powder. (289 mg, 81%). M.P.: 178-181 °C. $[\alpha]^{20}_D$ = -1047 (c = 0.05 CH_2Cl_2); ^1H NMR (500 MHz, $CDCl_3$) δ 9.28 (d, J = 8.4 Hz, 1H), 7.87 (d, J = 8.2 Hz, 1H), 7.73 (d, J = 8.2 Hz, 1H), 7.67 (m, 1H), 7.49 – 7.57 (m, 1H), 7.20 – 7.07 (m, 2H), 7.05 (d, J = 8.0 Hz, 1H), 6.94 – 6.75 (m, 2H), 4.94 – 4,84, (m, 1H), 4.54 – 5.41 (m, 1H), 4.34 – 4.30 (m, 1H), 3.96 (s, 1H).^{13}C NMR (126 MHz, $CDCl_3$) δ 147.1,

140.3, 137.8, 135.1, 131.2, 130.8, 130.2, 129.1, 129.0, 127.7, 126.5, 125.5, 125.4, 124.9, 123.2, 119.9, 101.5, 90.9, 83.5, 72.2, 64.0, 62.5. IR (ATR) ν_{max} 3677, 3436, 2980, 1172 cm^{-1}. HRMS: (ESI-TOF) calculated for $C_{44}H_{28}O_2Cl_2Fe$ $[M]^+$ 714.0816, found 714.0820.

Table S1. Control reactions for investigation of the mechanism of dehalogenation of 2-chloro diketone.

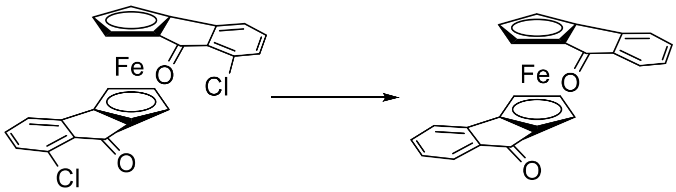

Entry	Change	Base	Solvent	Dehalogenation (%)[a]
1	Standard[b]	KO*t*Bu	IPA	82
2	Pyridine[c]	KO*t*Bu	IPA	81
3	BHT	KO*t*Bu	IPA	80
4	-	Cs_2CO_3	IPA	82
5	No PEPPSI	KO*t*Bu	IPA	50
6	-	KO*t*Bu	*t*BuOH	34
7	No Boronic	KO*t*Bu	IPA	80
8	No Base	-	IPA	0

a. Dehalogenation measured using SFC-UV analysis after recording extinction coefficient value for starting material and dehalogenation product. [b.] Diketone 7c (1 eq) PEPPSI-*i*Pr (6 mol%), Base (4 eq), TRIP boronic acid (2 eq), Pentafluorophenyl boronic acid (2 eq), 80 °C, 20 h, N_2. [c.] No PEPPSI-*i*Pr precatalyst.

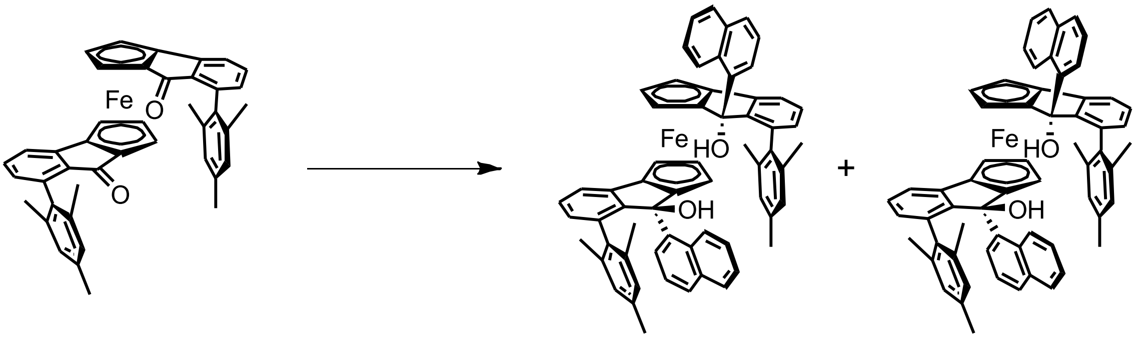

8i **13i** (*epi*)-**13i**

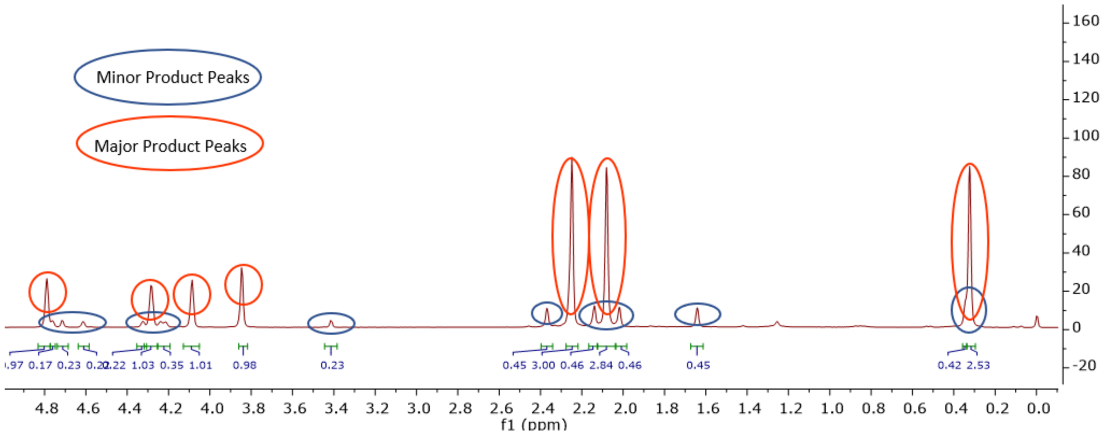

Figure S1. ^{1}H NMR Spectrum of 2-mesityl naphthyl diol. Addition of 1-naphthyl lithium gives rise to diastereomeric isomers as a result of uncontrolled axially chirality about the newly formed C_{α}-C_{naph} bond. Based on X-ray crystal structures of other naphthyl diols, major isomer is tentatively assigned as **13i**, where the B ring of the naphthalene projects outwards, and the minor isomer is assumed to be (*epi*)-**13i**, where the B ring is directed back across the catalyst backbone.

General procedure for hetero-Diels Alder reaction:

The diol catalyst (0.05 mmol, 0.2 eq) was added to a dry, N2 flushed 10 mL Schlenk flask equipped with a magnetic stirring bar followed by 2 mL of anhydrous toluene. The aldehyde (0.50 mmol, 2.0 eq) was added with stirring at RT before cooling to -78 ºC. After 30 min the diene (65 µL, 0.25 mmol, 1.0 eq) was added and the reaction was left to stir for 48 h. The reaction was quenched at -78 ºC by the addition of acetyl chloride (70 µL, 1.0 mmol, 4.0 eq) followed by dilution with CH_2Cl_2 (1 mL). The crude reaction mixture was purified directly by SiO_2 chromatography (8:2 cyclohexane/EtOAc).

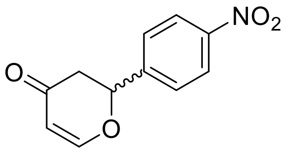

2-(4-Nitrophenyl)-2,3-dihydro-4H-pyran-4-one

^{1}H NMR (300 MHz, $CDCl_3$) δ 8.33 – 8.25 (m, 2H), 7.64 – 7.56 (m, 2H), 7.51 (d, *J* = 6.1 Hz, 1H), 5.58 (dd, *J* = 6.1, 1.2 Hz, 1H), 5.55 (dd, *J* = 13.7, 4.2 Hz, 1H), 2.85 (dd, *J* = 16.8, 13.7 Hz, 1H), 2.72 (ddd, *J* = 16.8, 4.2, 1.2 Hz, 1H); TLC (pentane:EtOAc, 4:1), Rf: 0.15 (UV). SFC analysis (CHIRALPAK IA, CO2/MeOH 99/1 to 60/40 over 7 min, 3 mL/min) t_R = 4.34 min (*R*) and t_R = 6.01 min (*S*). All physical data were identical to those previously reported.[2]

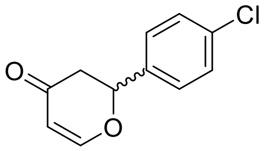

2-(4-Chlorophenyl)-2,3-dihydro-4H-pyran-4-one

^{1}H NMR (300 MHz, $CDCl_3$) δ 7.47 (dd, *J* = 6.1, 0.7 Hz, 1H), 7.43– 7.29 (m, 4H), 5.53 (dd, *J* = 6.1, 1.3 Hz, 1H), 5.41 (dd, *J* = 14.2, 3.6 Hz, 1H), 2.86 (dd, *J* = 16.8, 14.2 Hz, 1H), 2.65 (ddd, *J* = 16.8, 3.6, 1.3 Hz, 1H); TLC (pentane:EtOAc, 4:1), Rf: 0.30 (UV). SFC analysis (CHIRALPAK IA, CO2/MeOH, 99/1 to 60/40 over 7 min, 3 mL/min) t_R = 3.14min (*R*) and t_R = 4.15 min (*S*). All physical data were identical to those previously reported.[2]

2-Cl Diketone **8a**

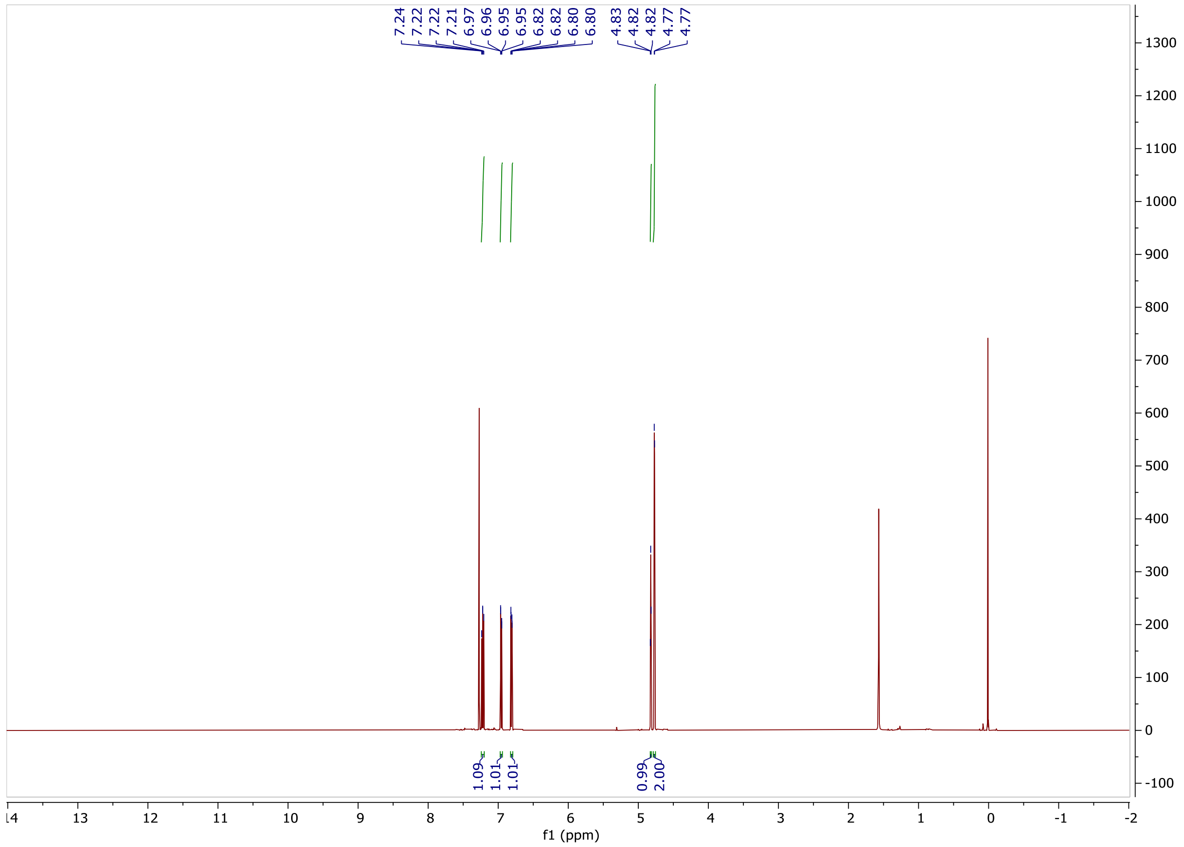

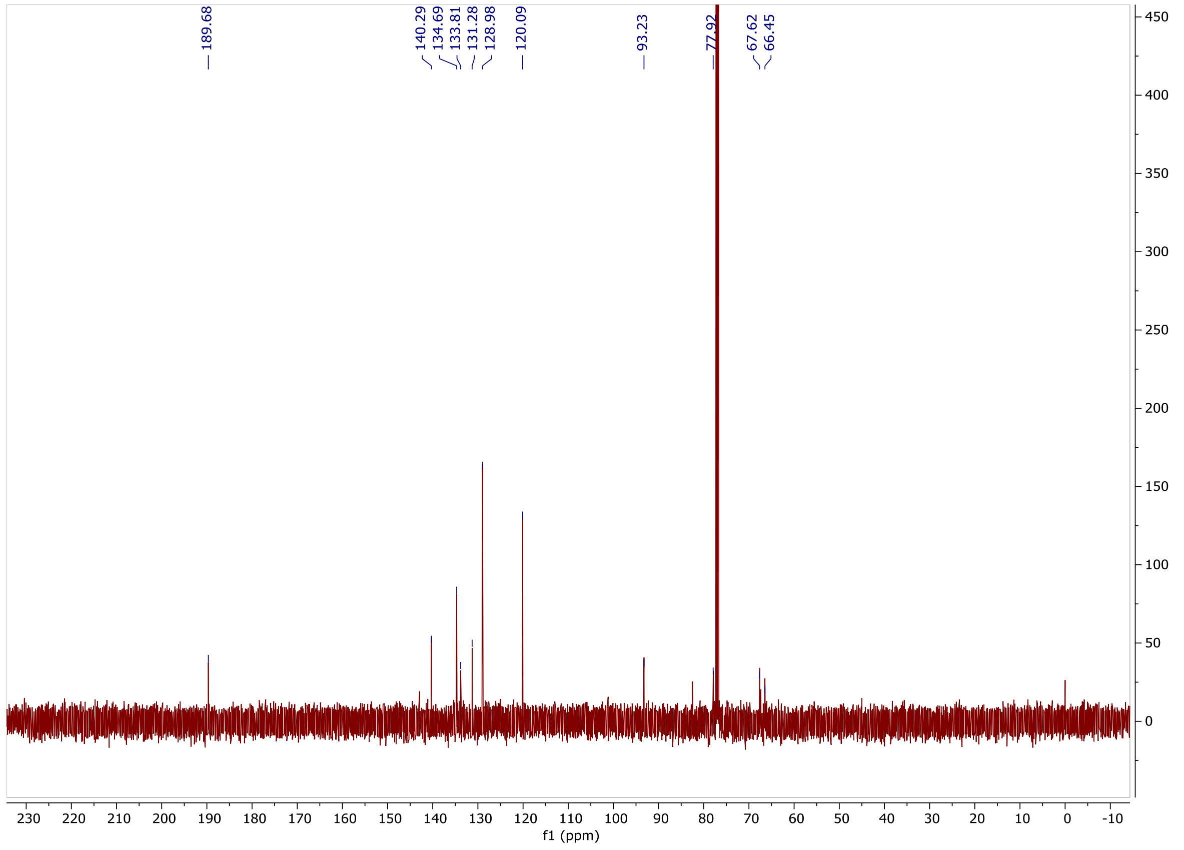

4-F, Diketone **8e**

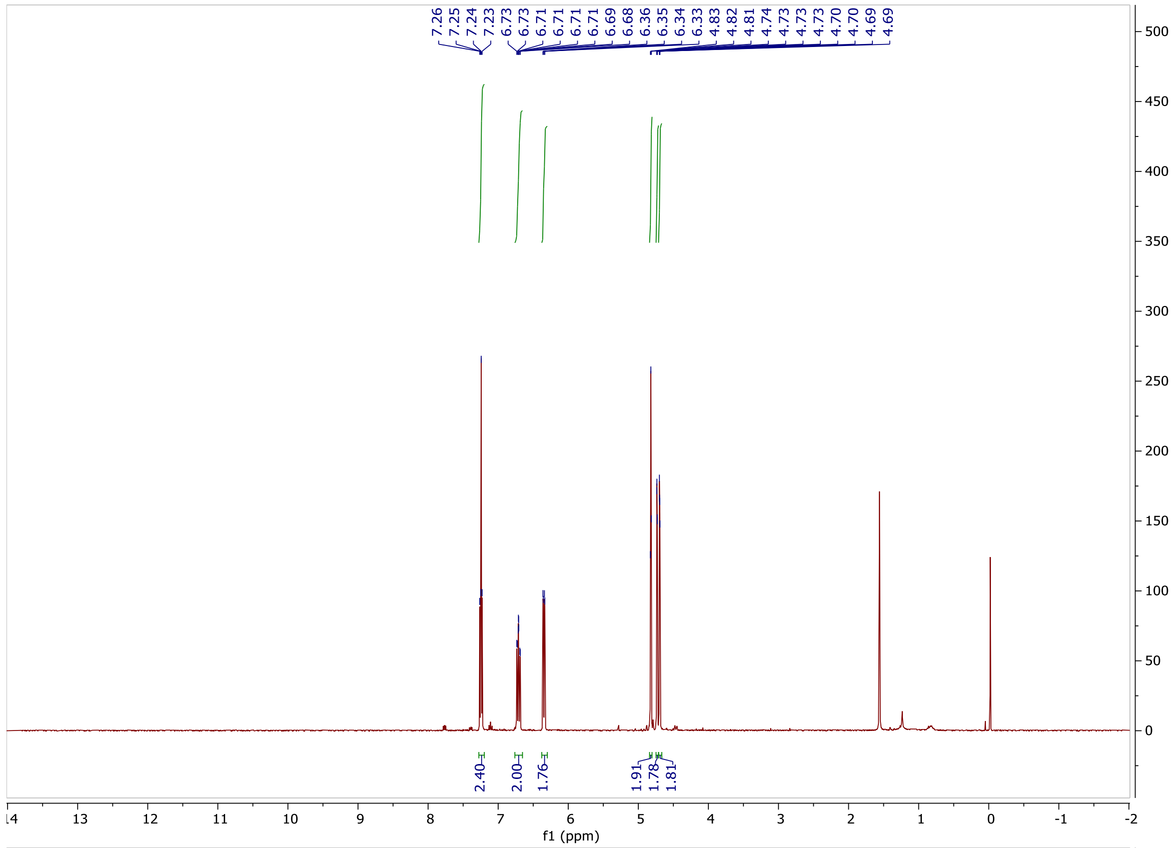

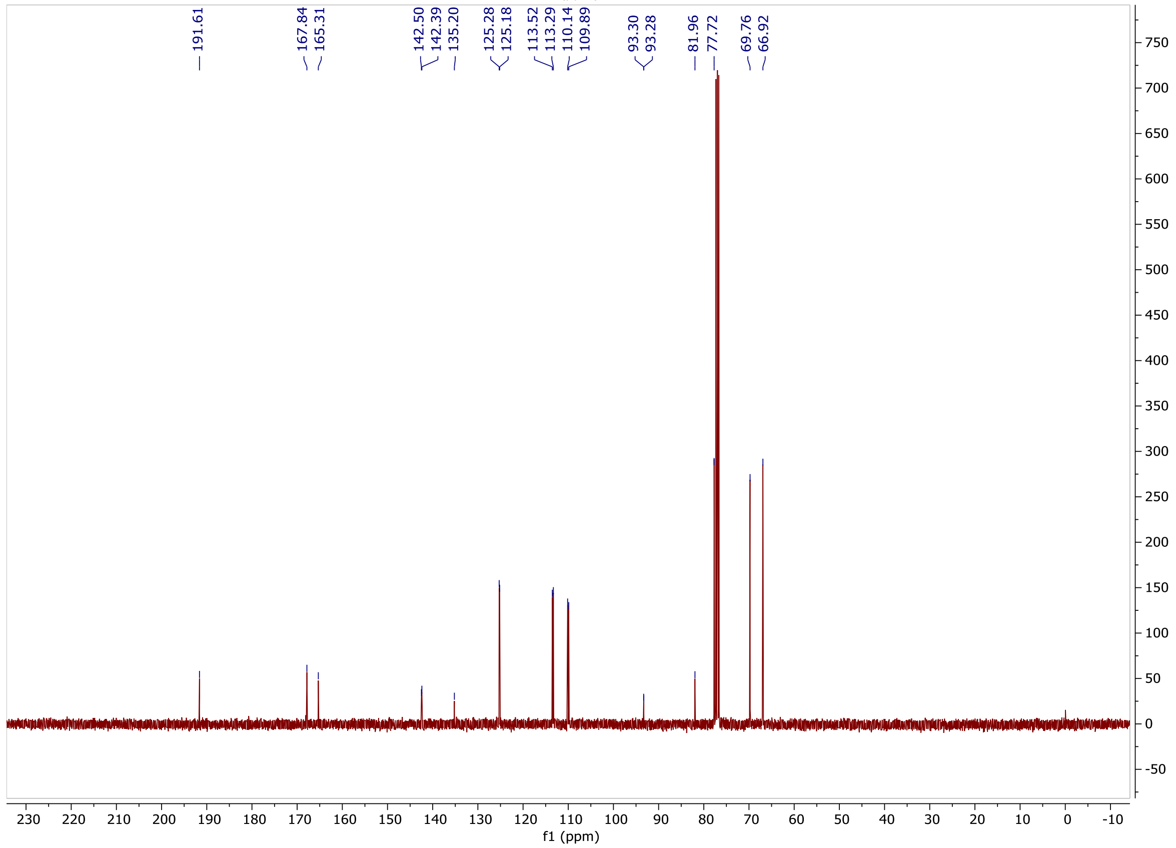

2-Me, Diketone **8c**

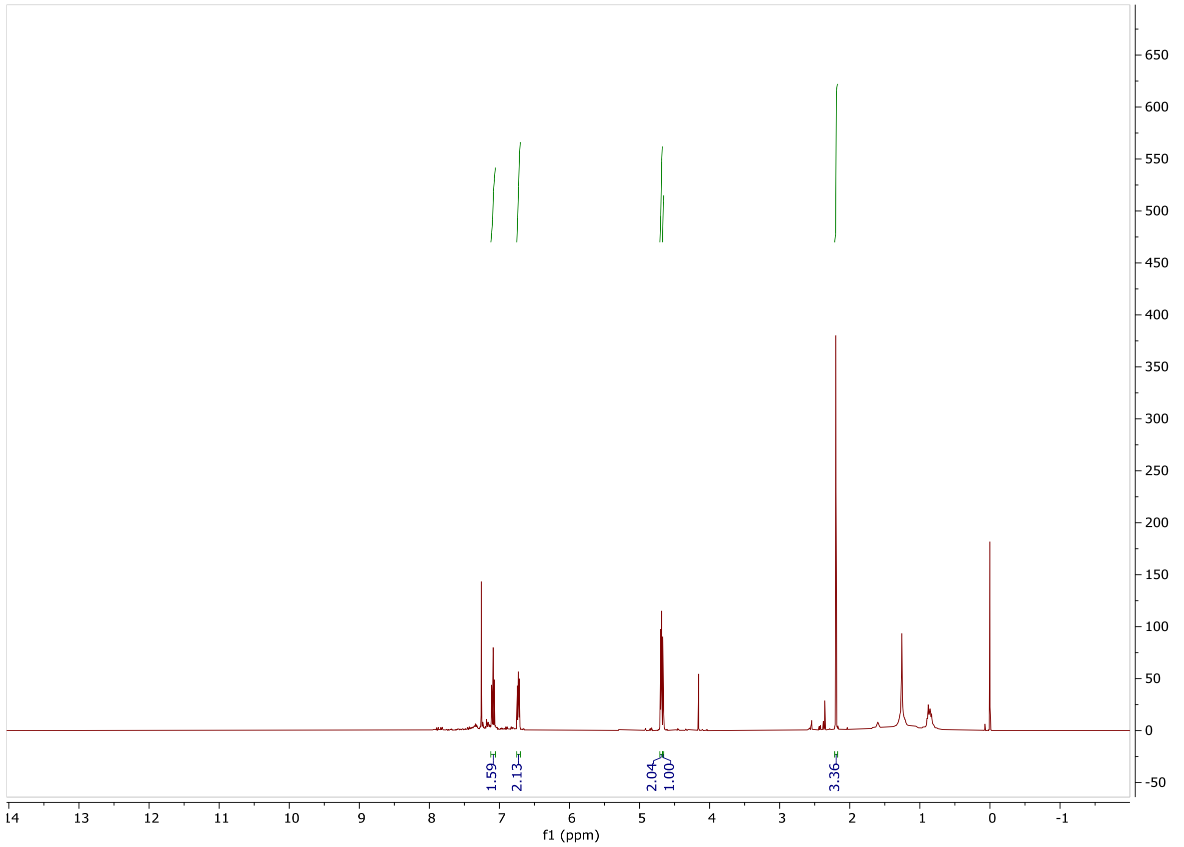

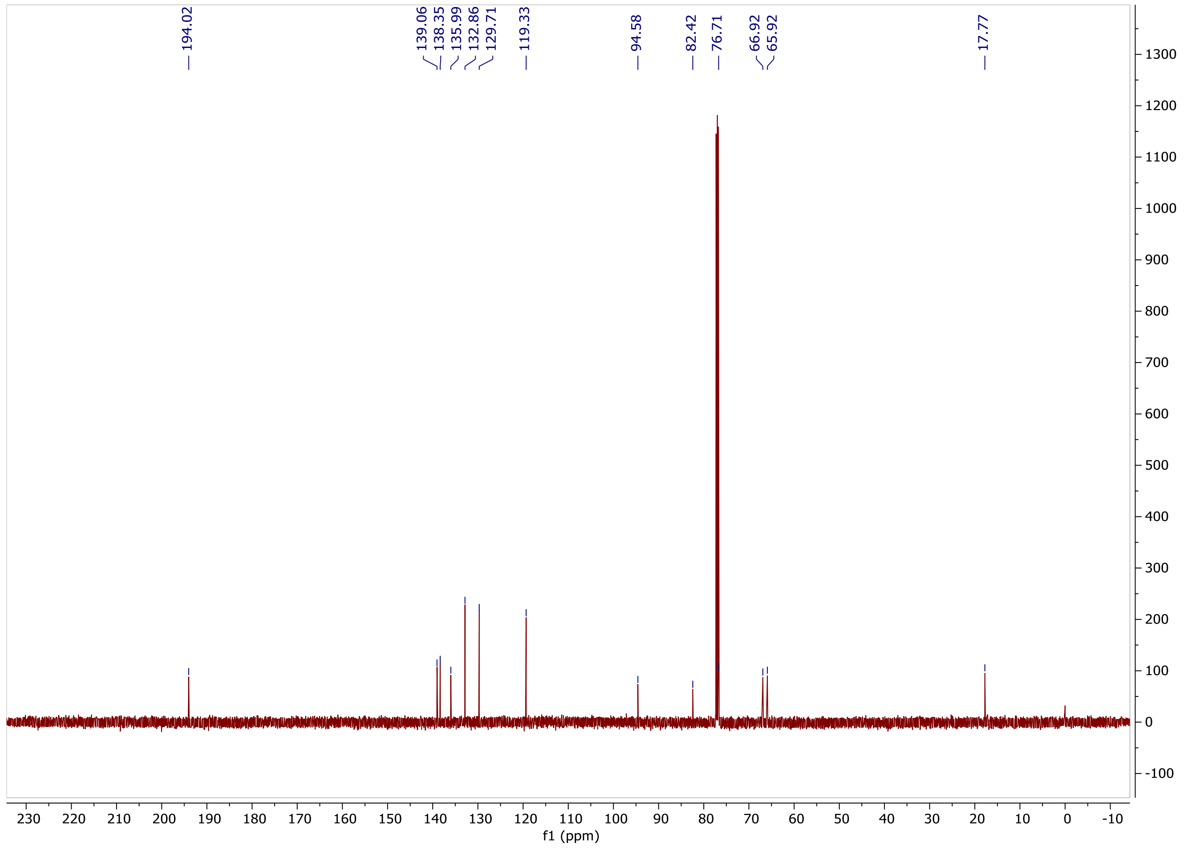

2-*p*-$MeOC_6H_4$ Diketone **8f**

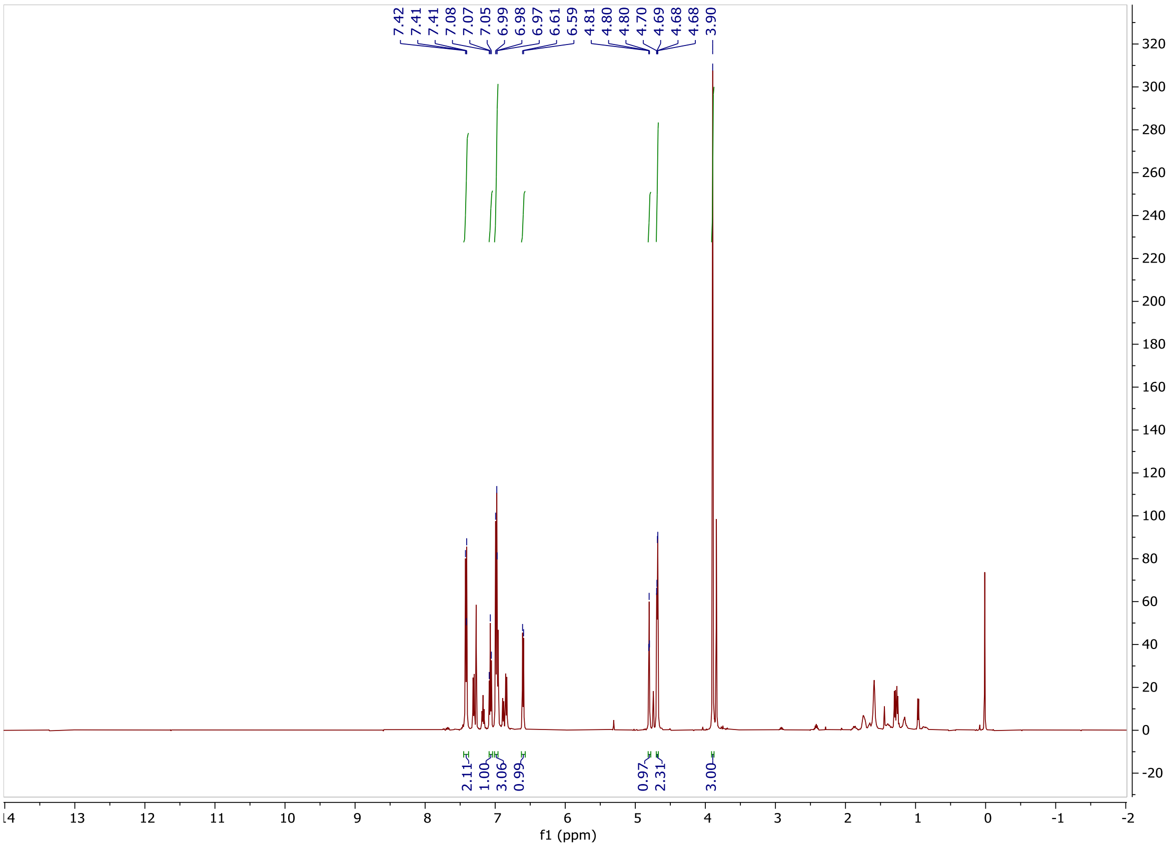

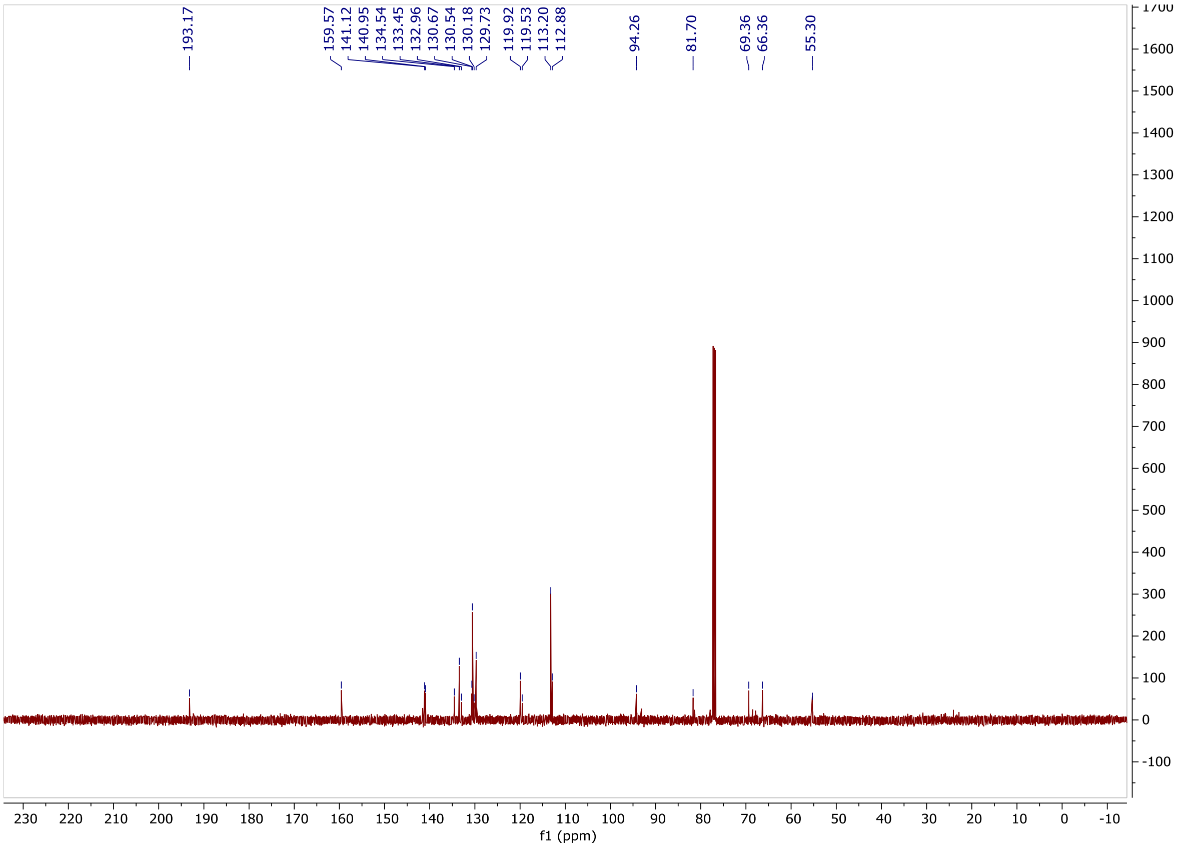

2-pCF$_3$C$_6$H$_4$ Diketone **8g**

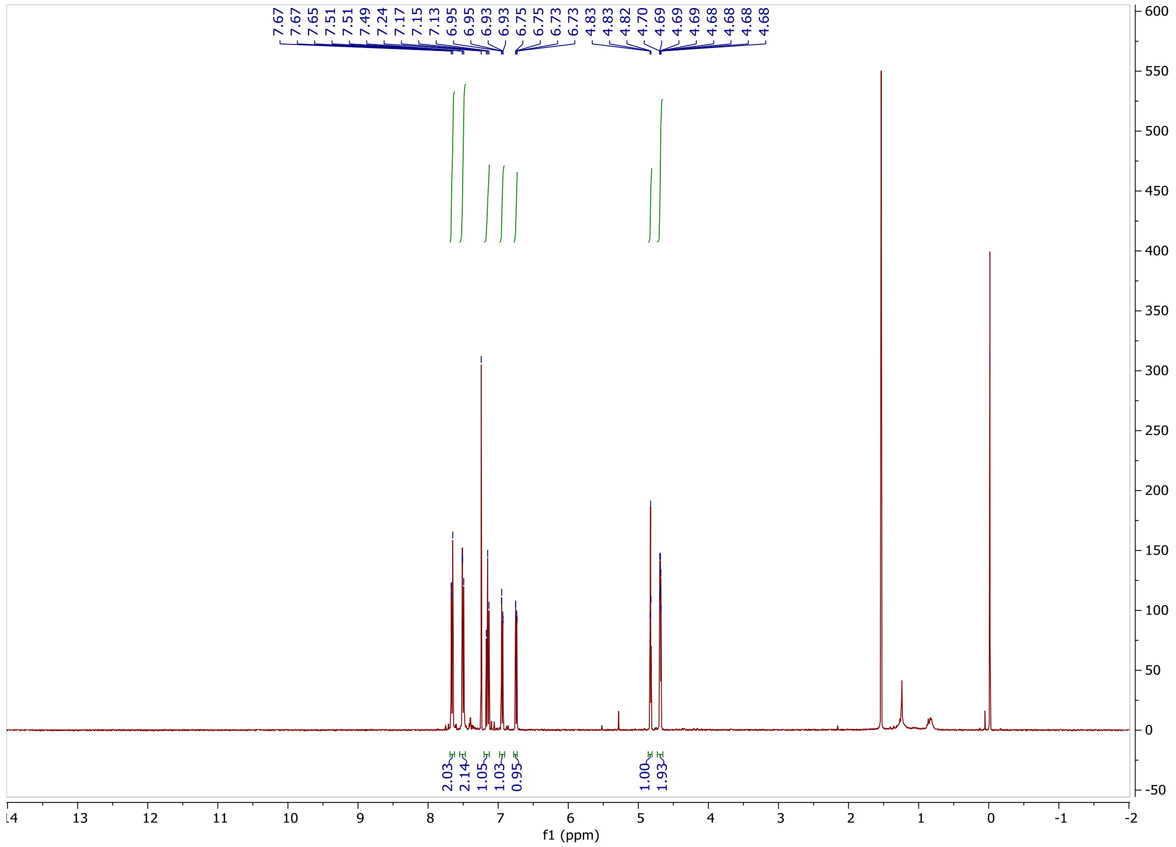

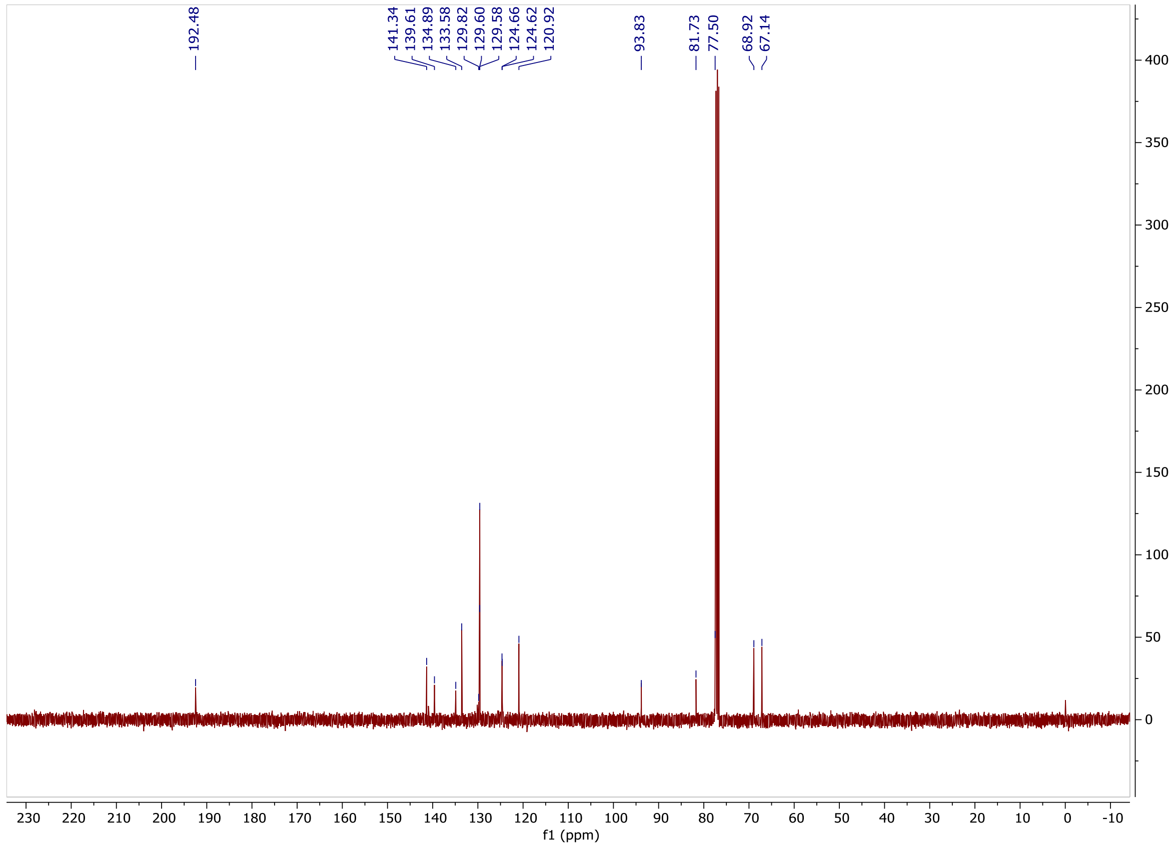

2-Ph, Diketone **8h**

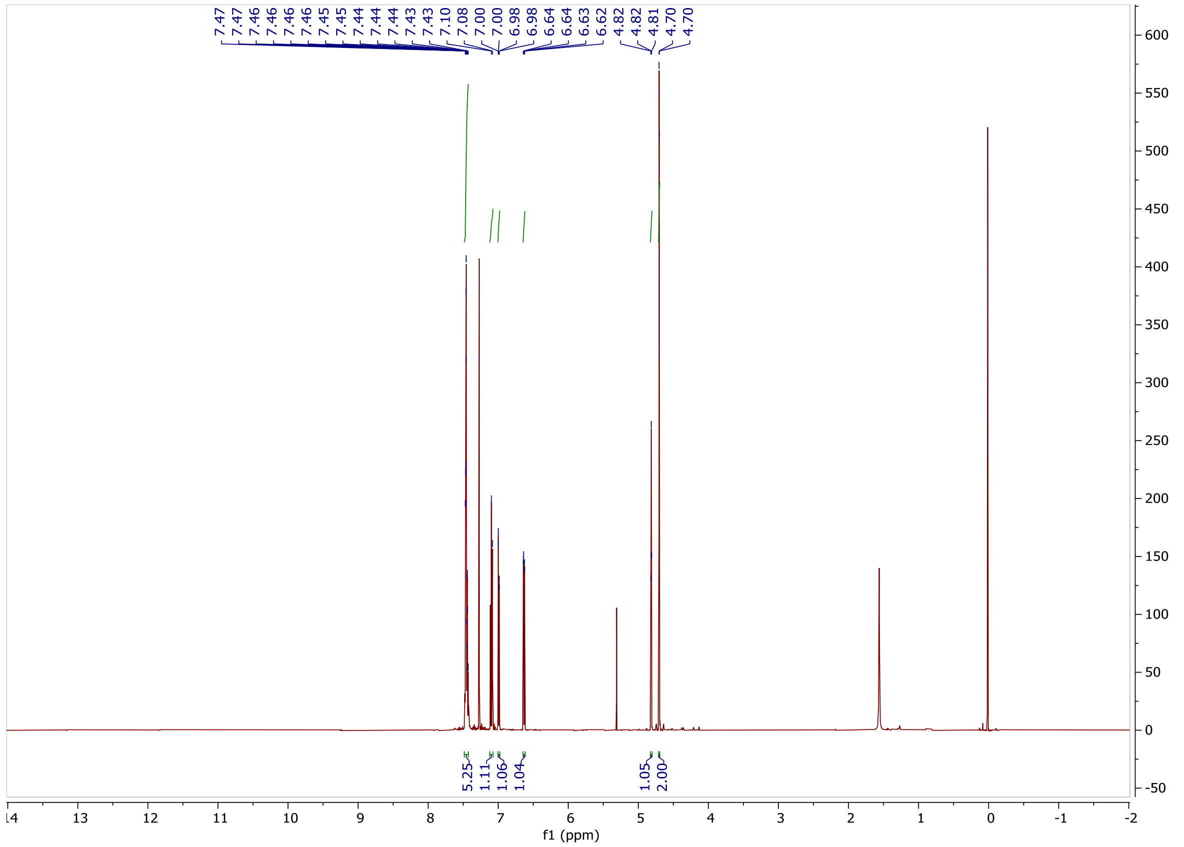

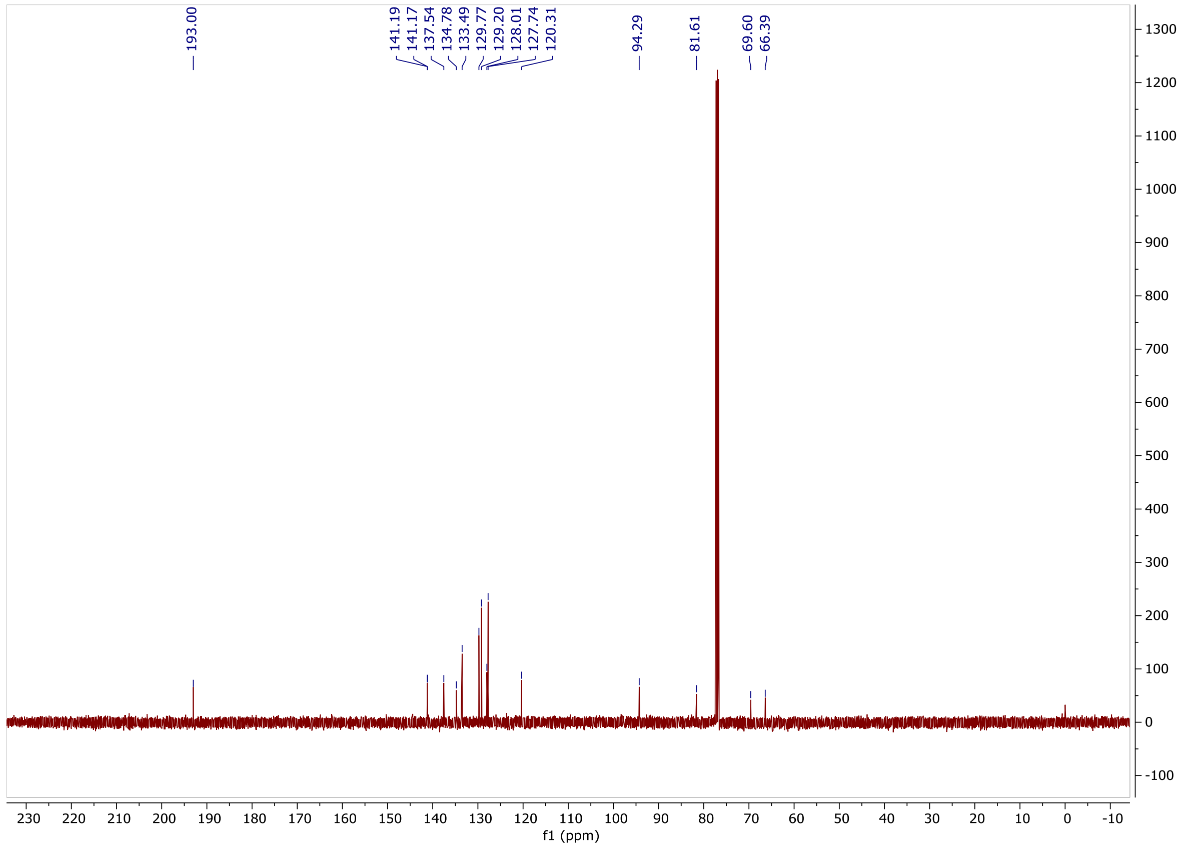

2-TRIP-Diketone **8i**

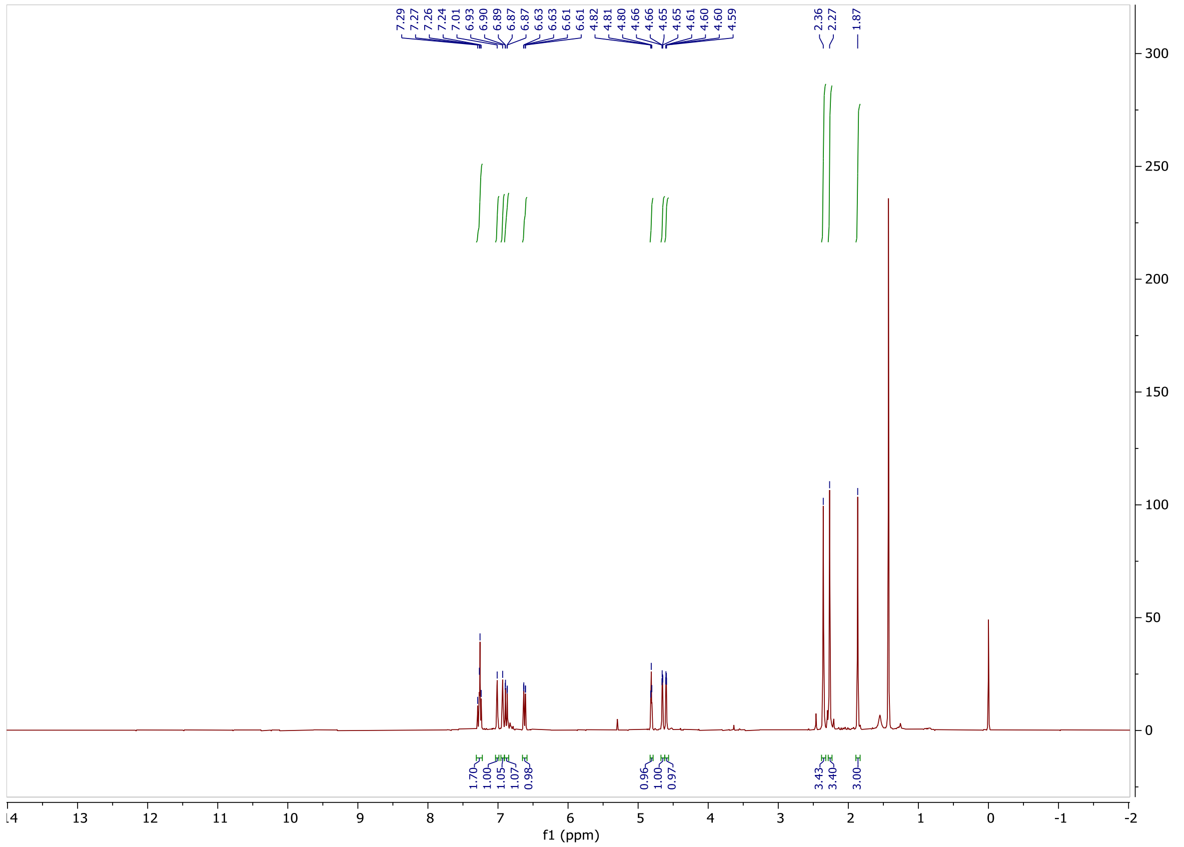

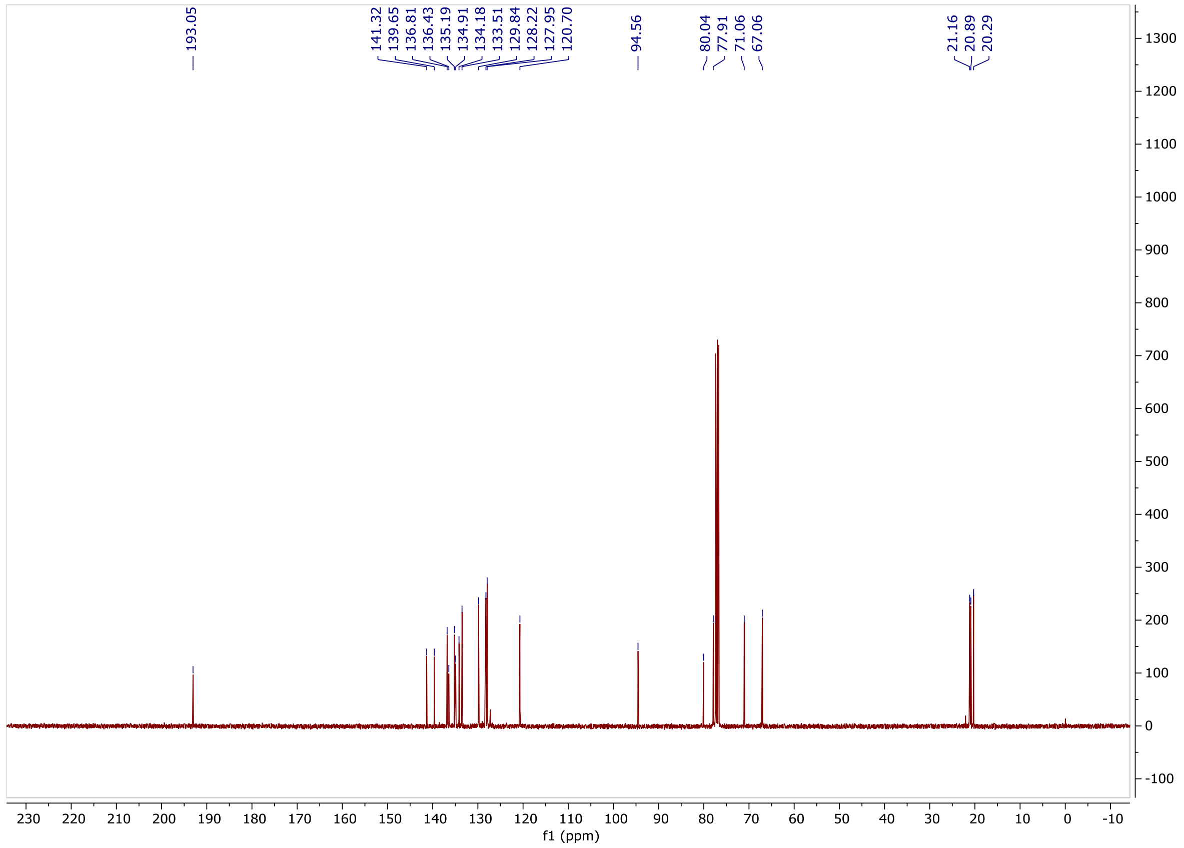

2-Cl, CF_3 Diol **14a**

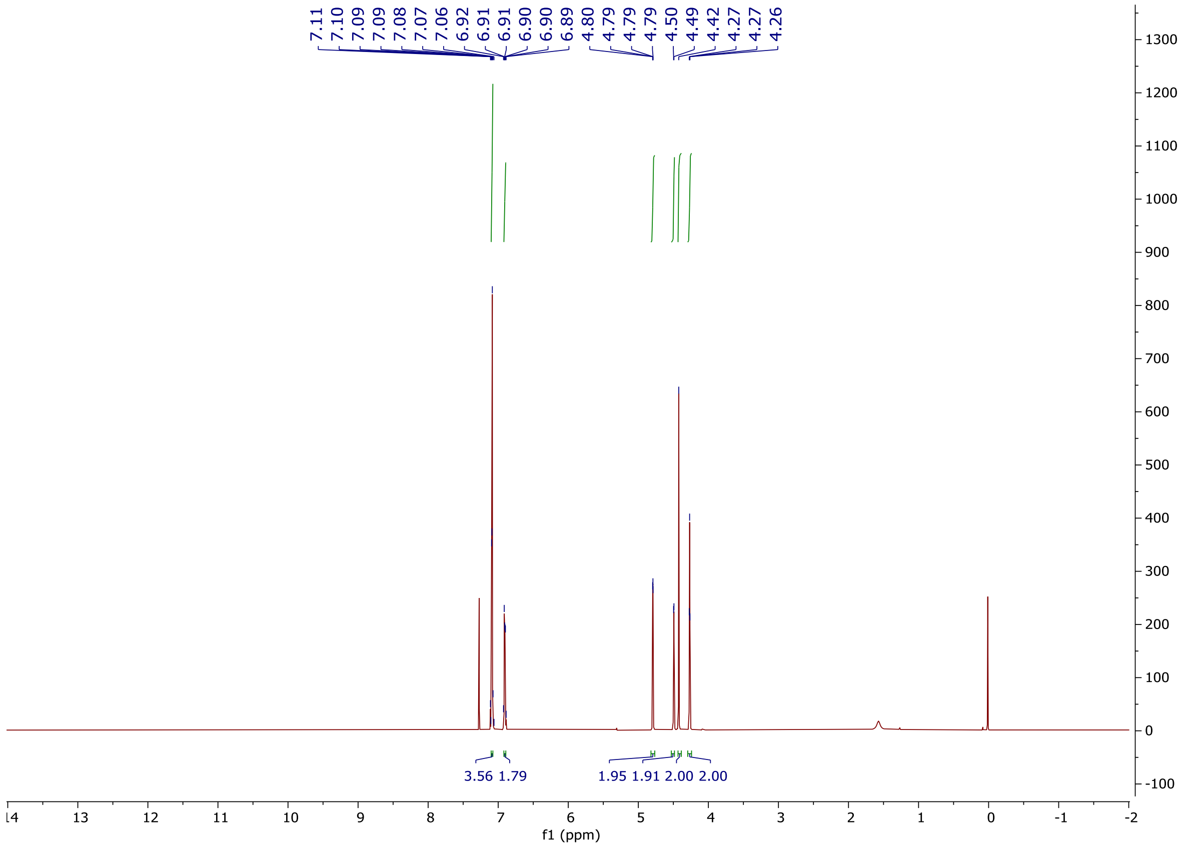

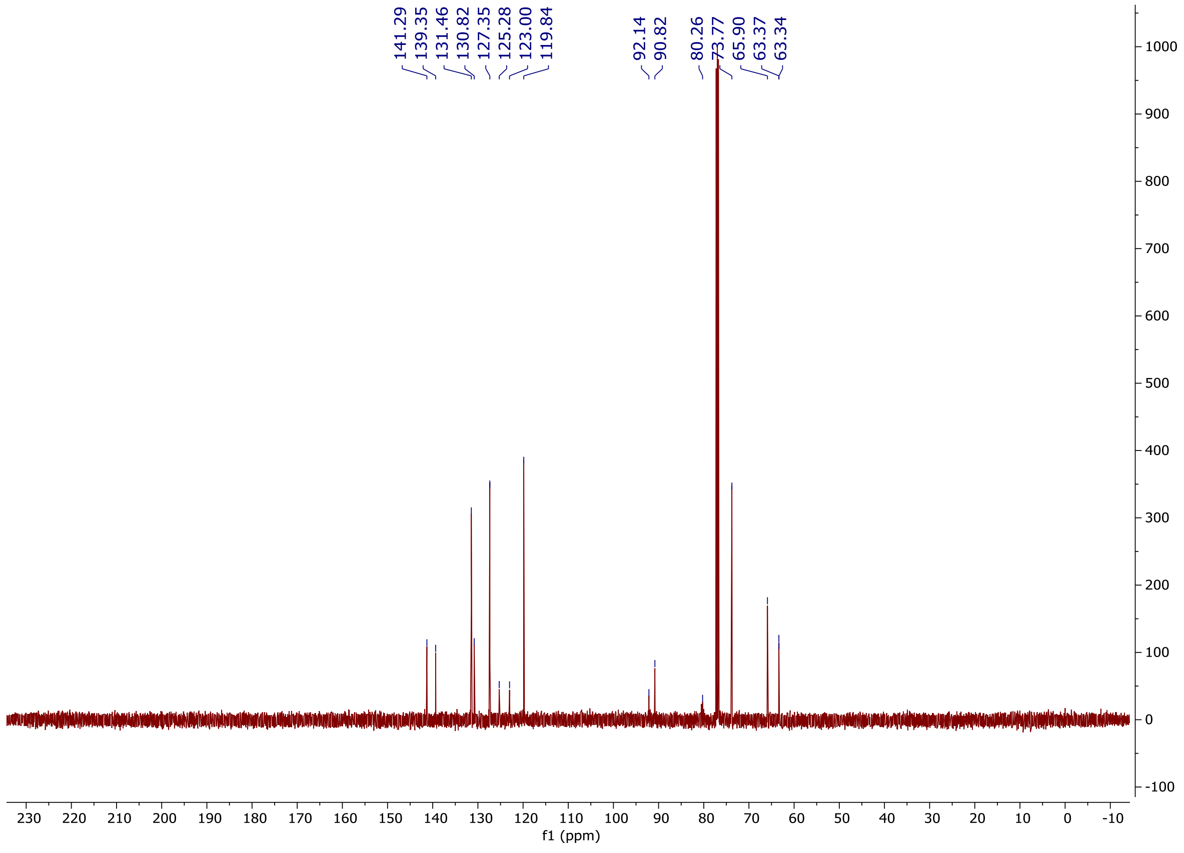

4-F, CF3 Diol **14e**

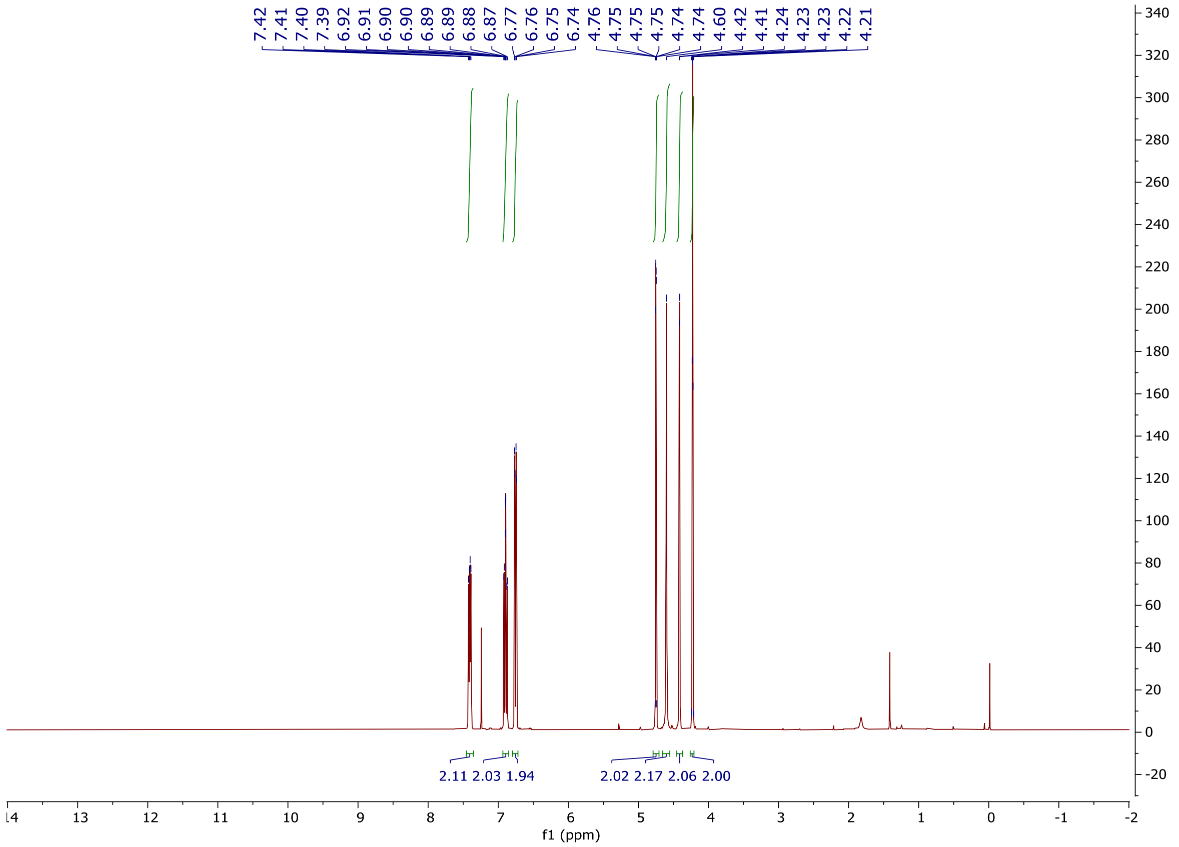

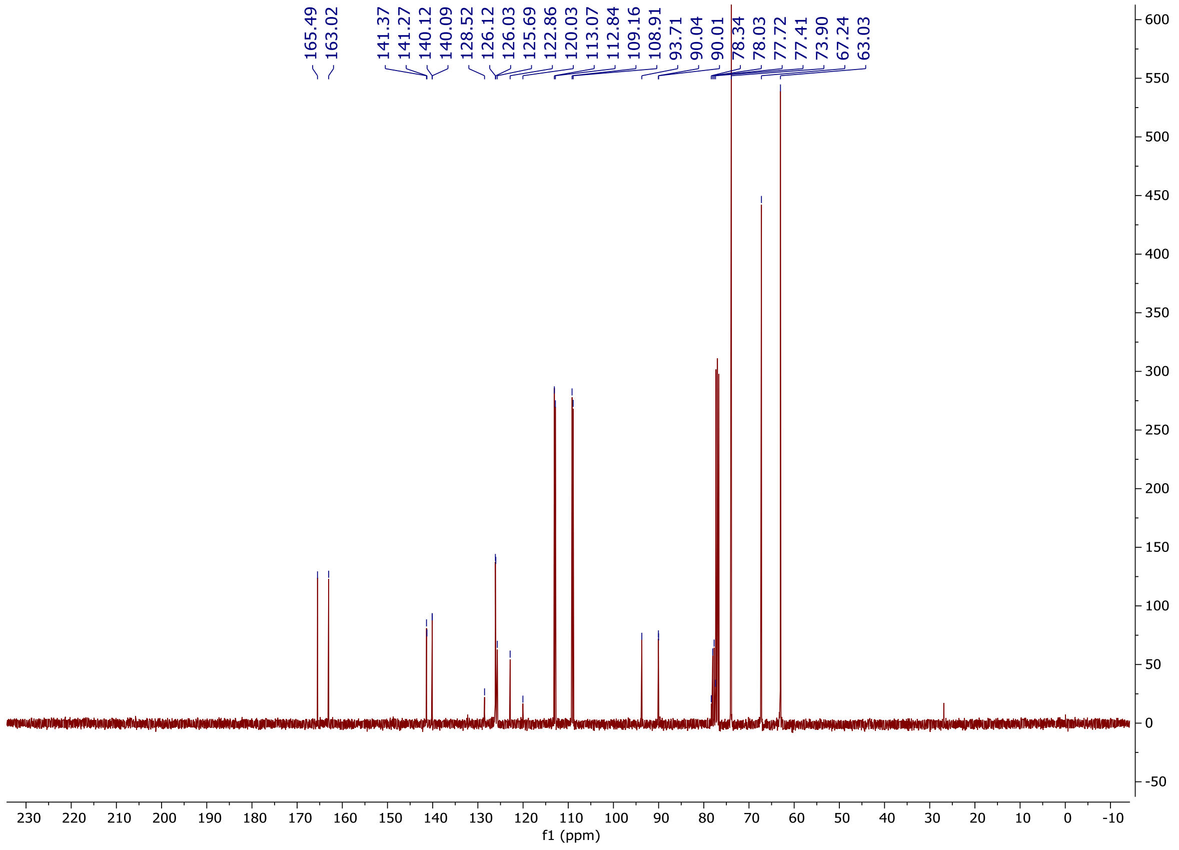

2-*p*OMeC_6H_4, CF_3 Diol **14f**

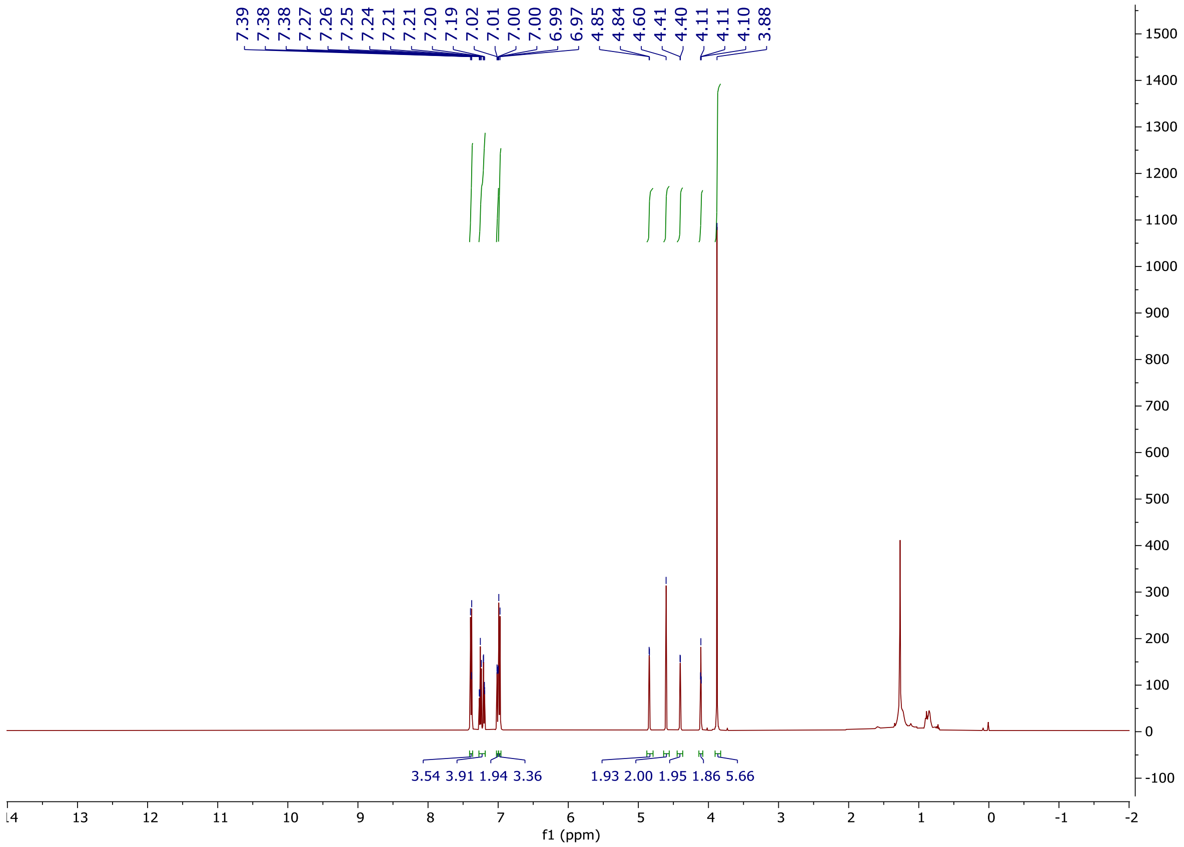

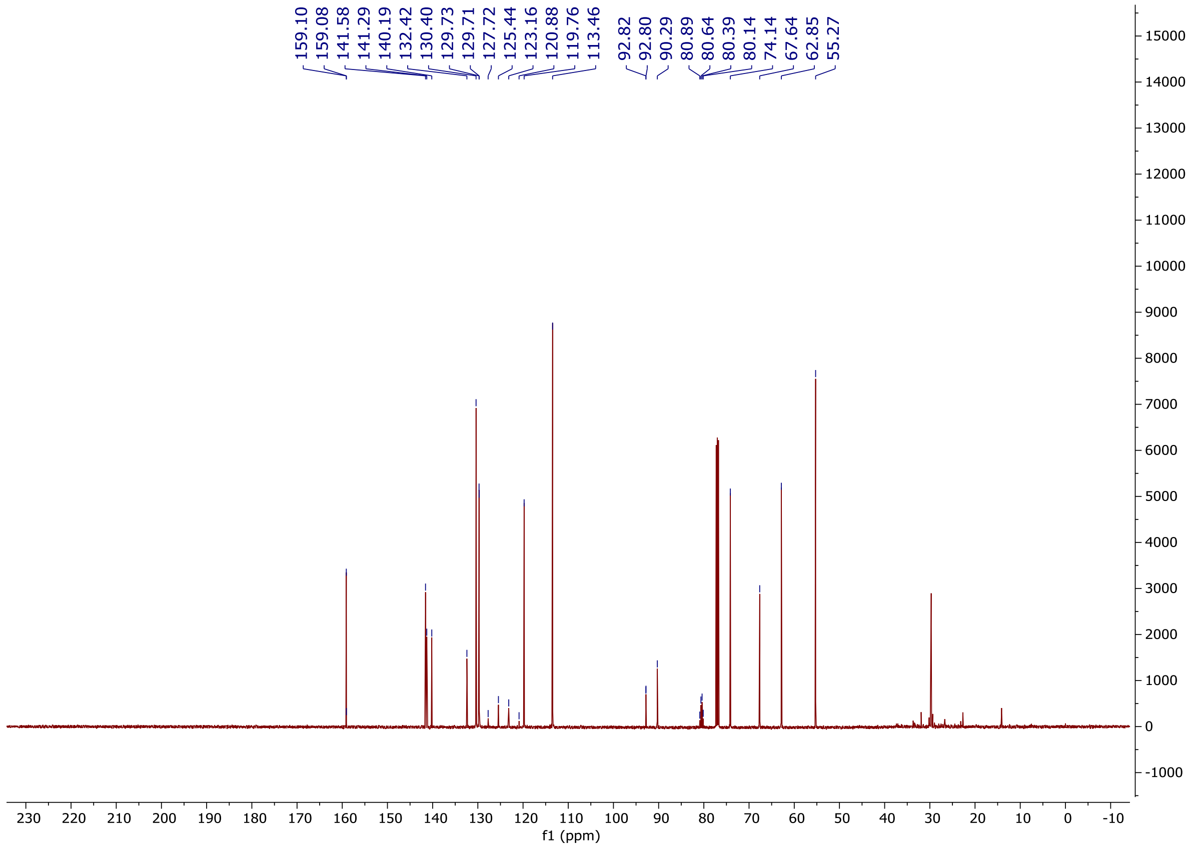

2-*p*$CF_3C_6H_4$, CF_3 Diol **14g**

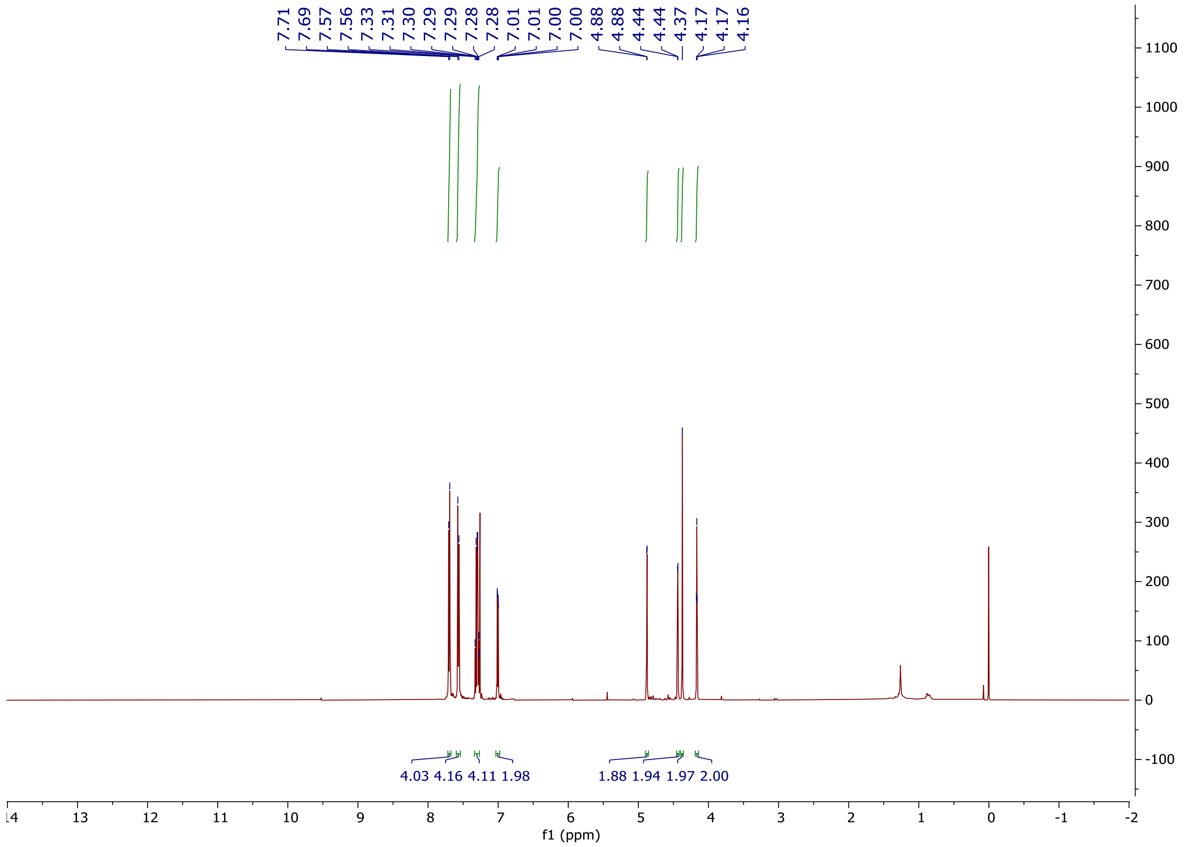

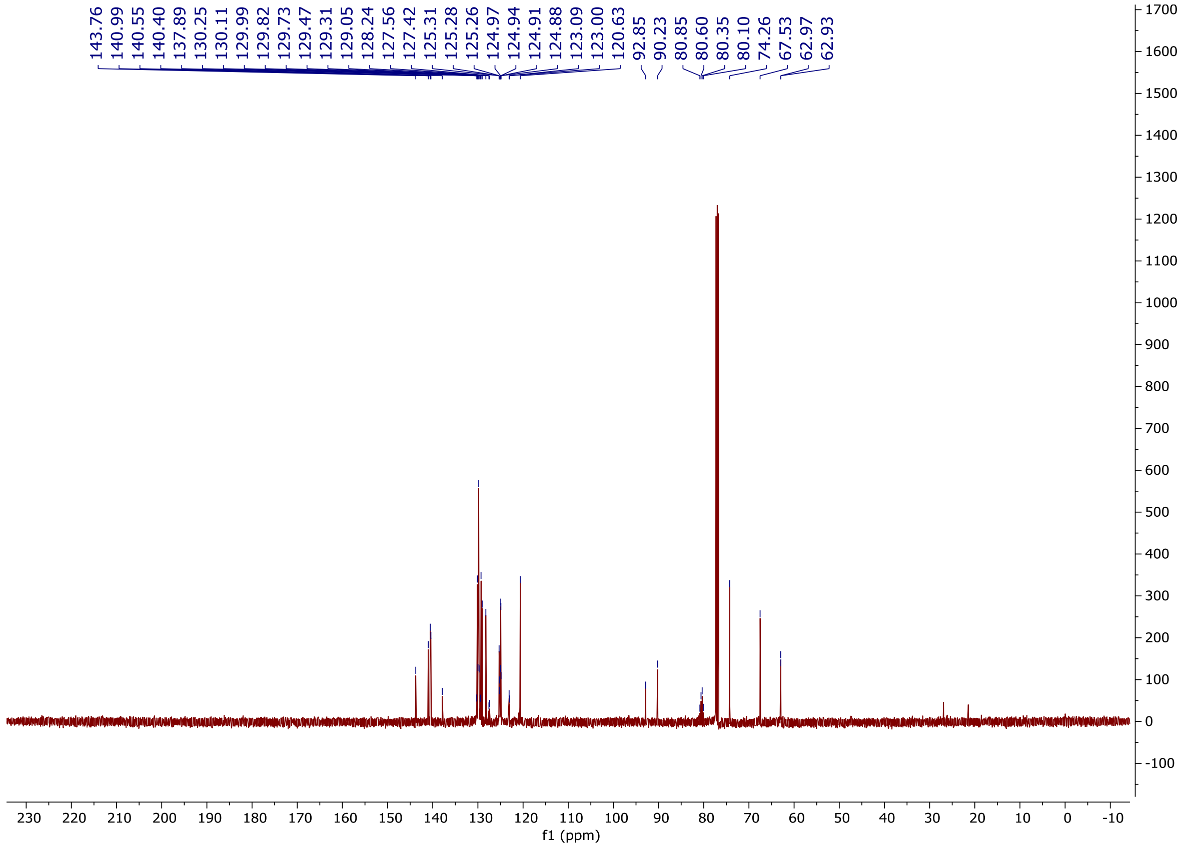

2-Ph, CF_3 Diol **14h**

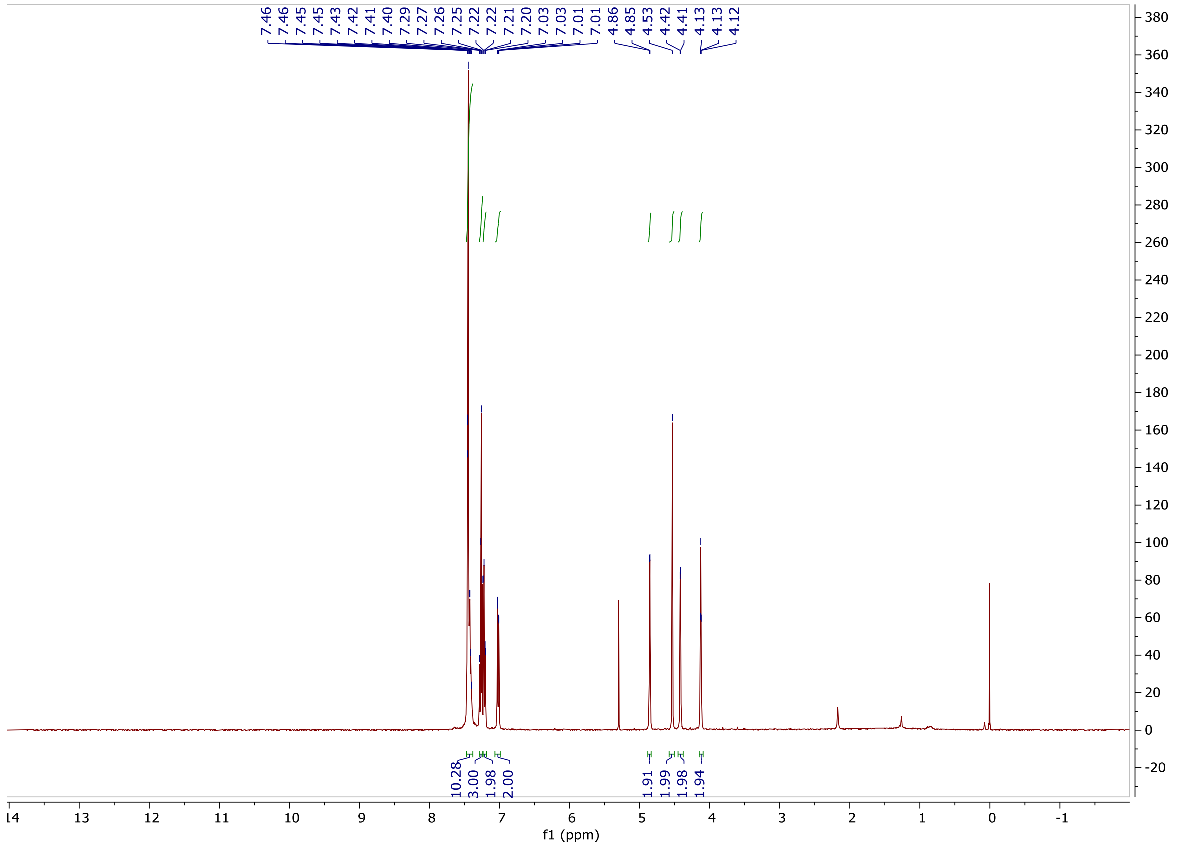

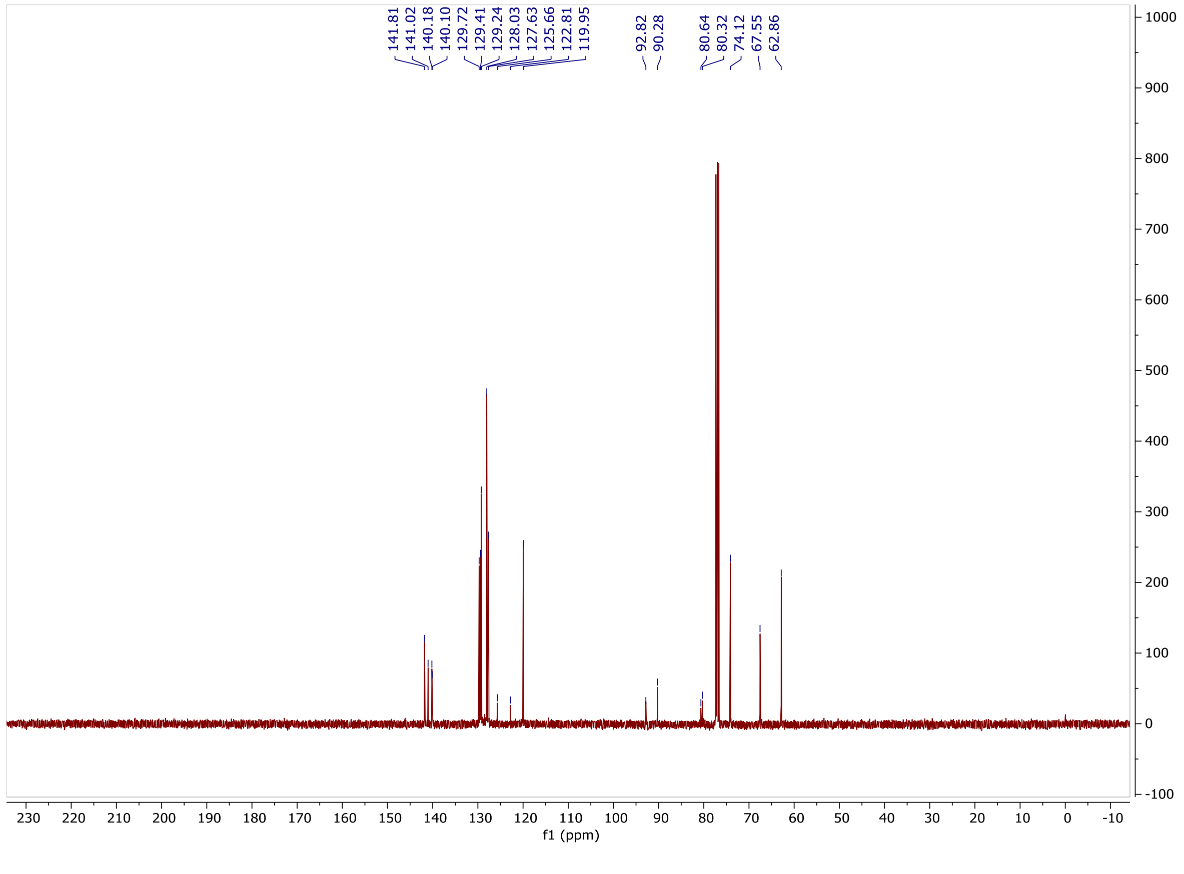

2-Mesityl, CF_3 Diol **14i**

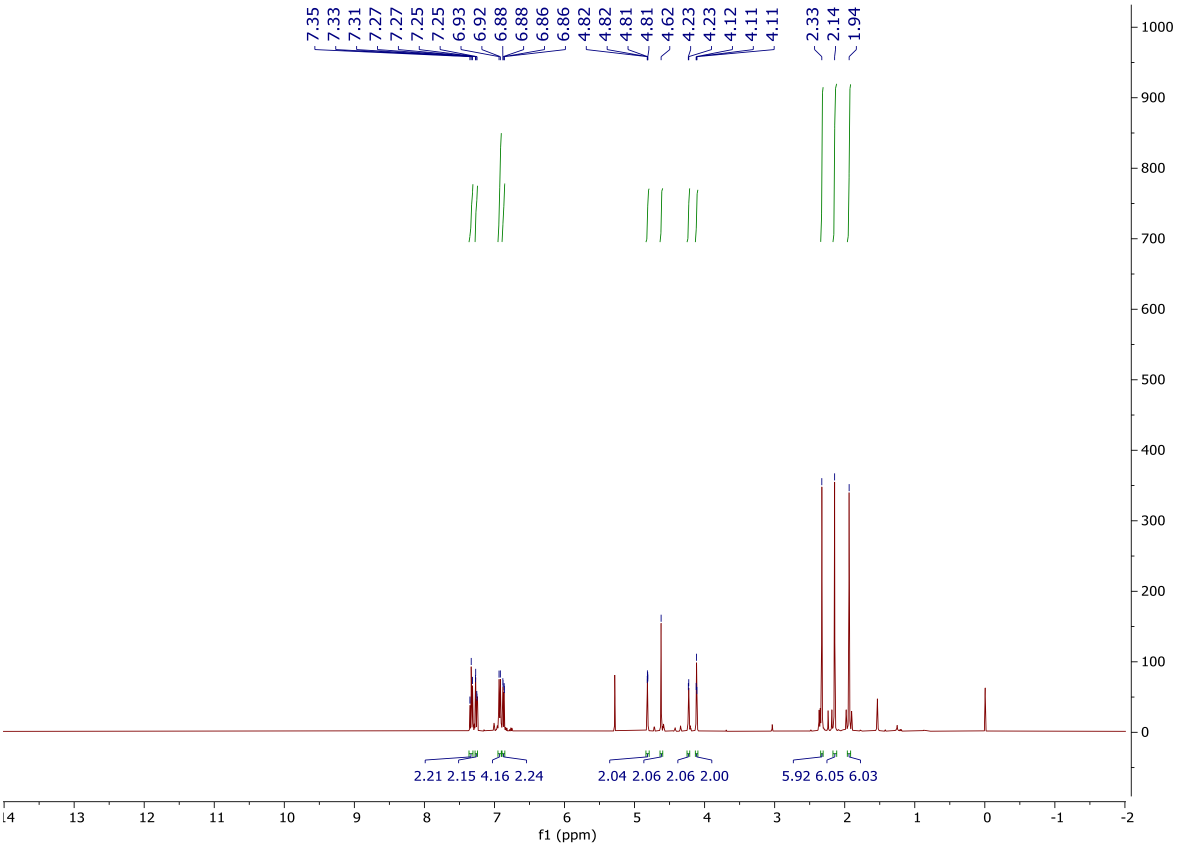

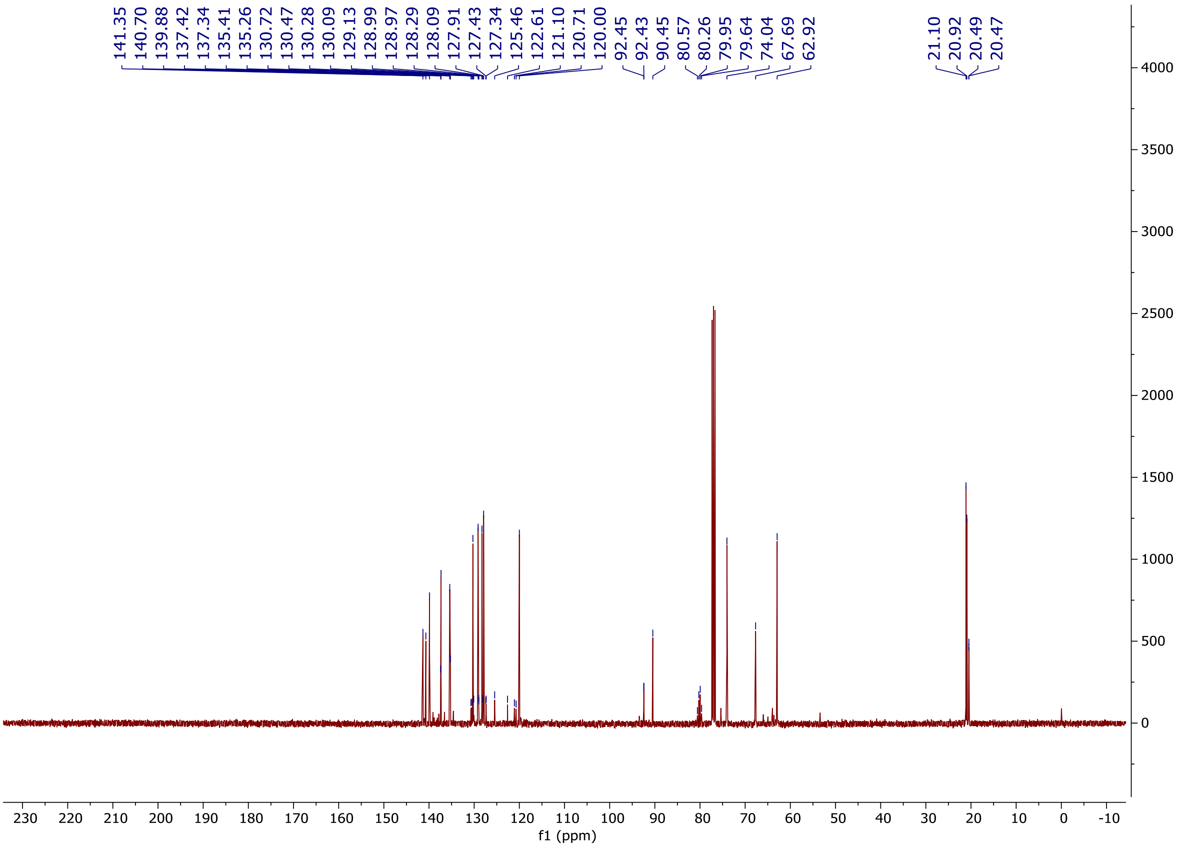

3,4-OMe, Naphthyl Diol **13d**

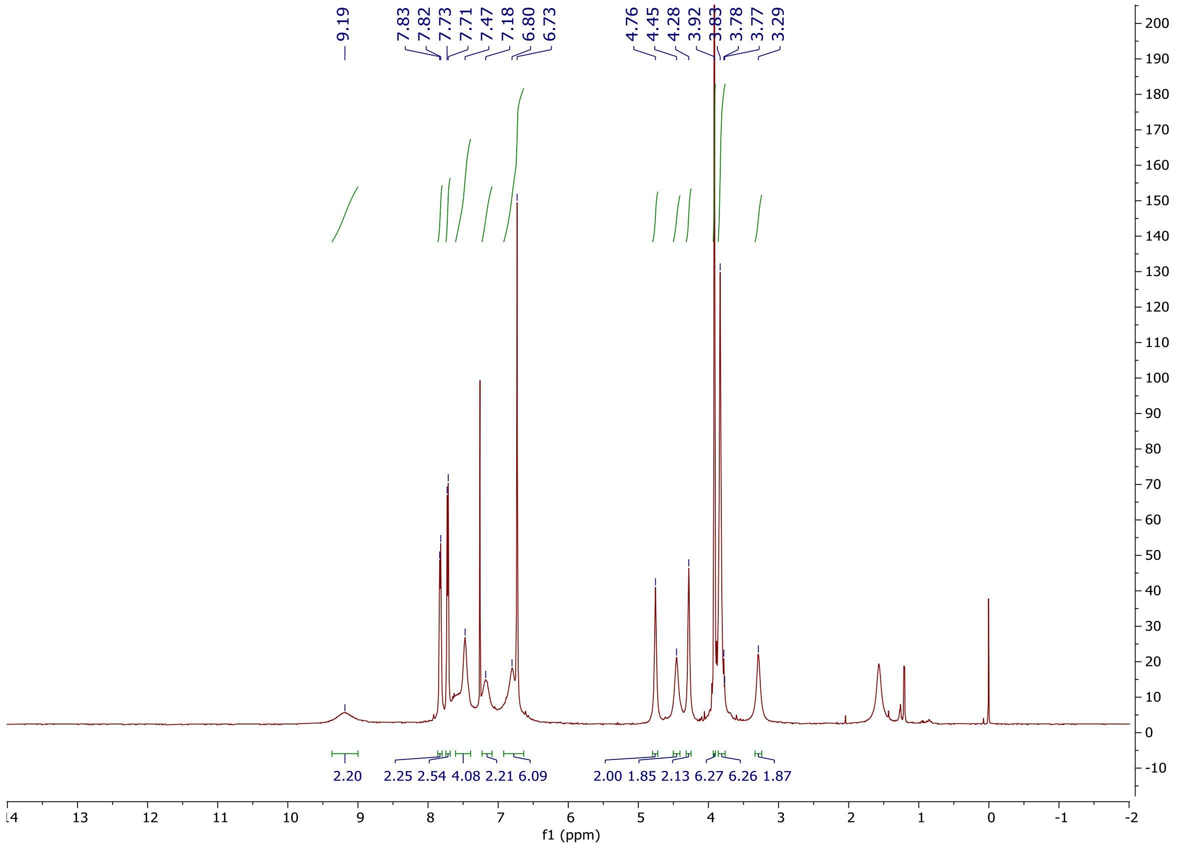

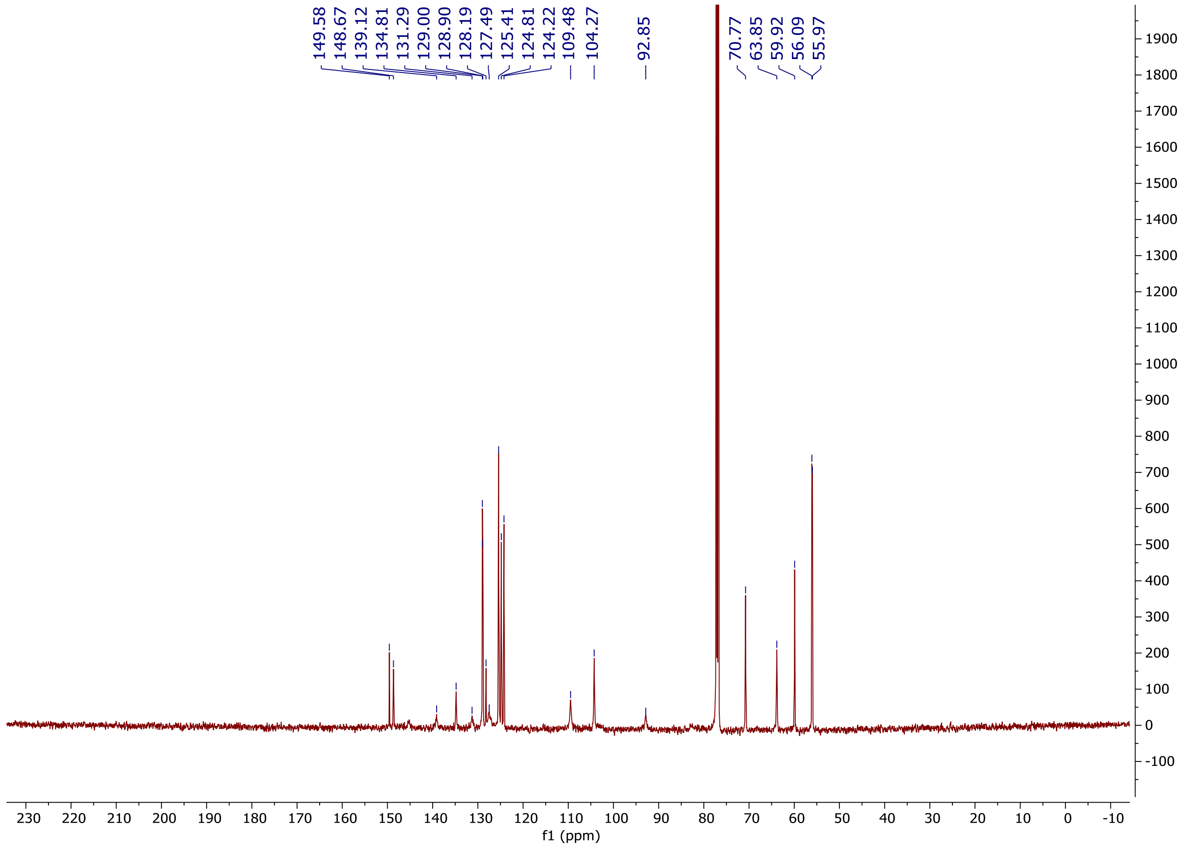

2-Me, Naphthyl Diol **13c**

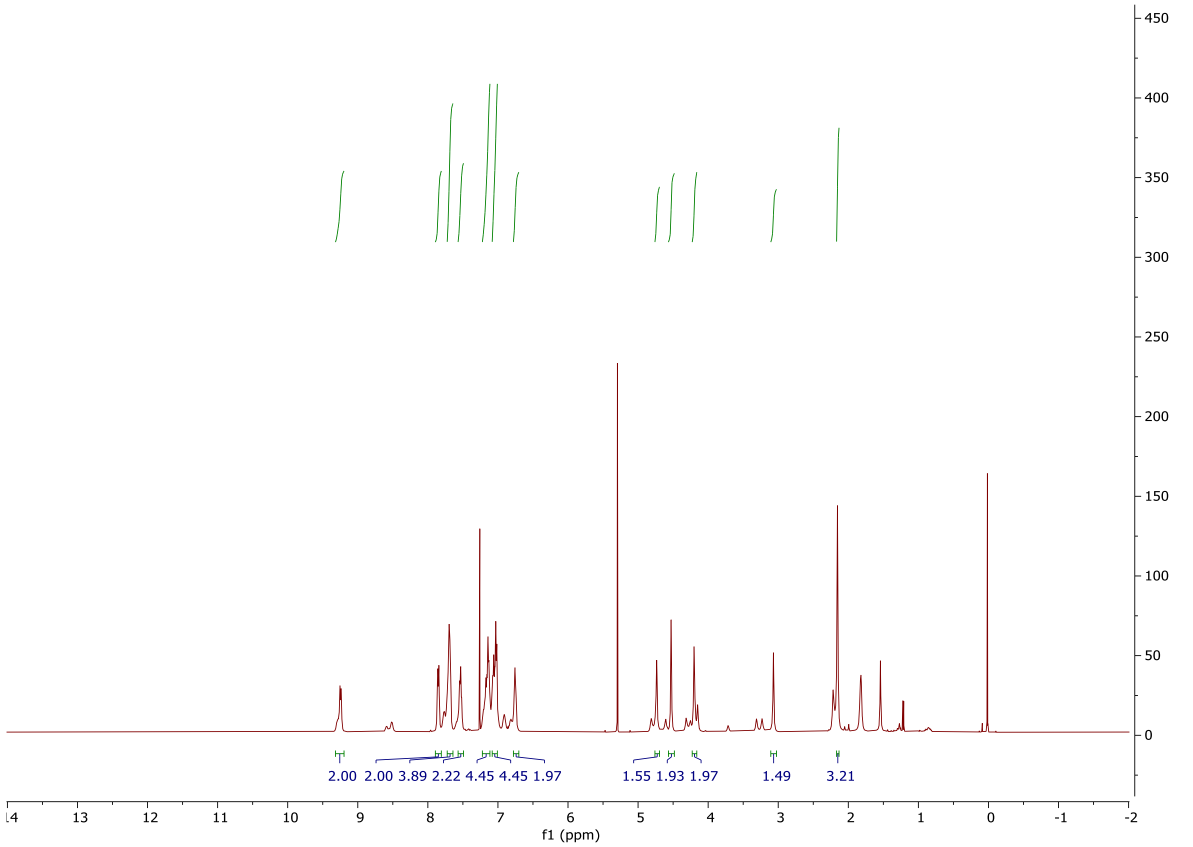

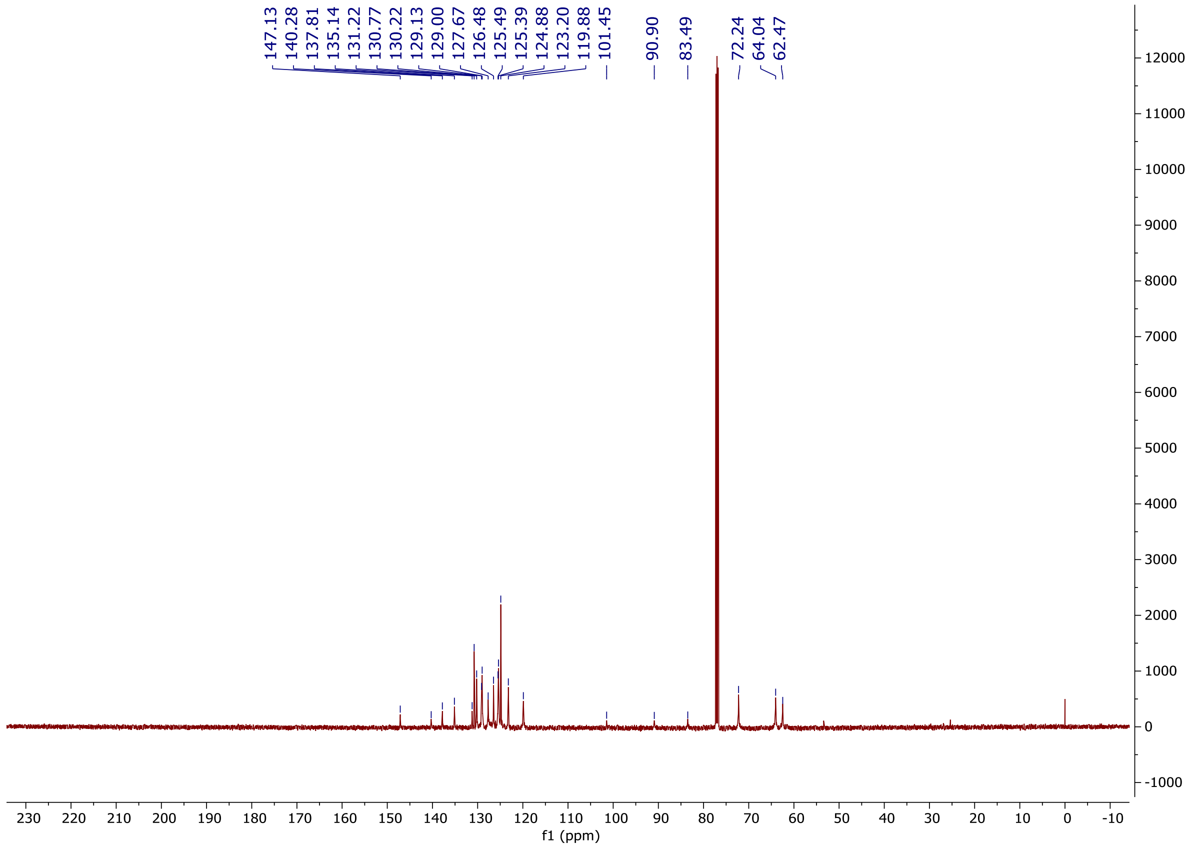

4-F, Naphthyl Diol **13e**

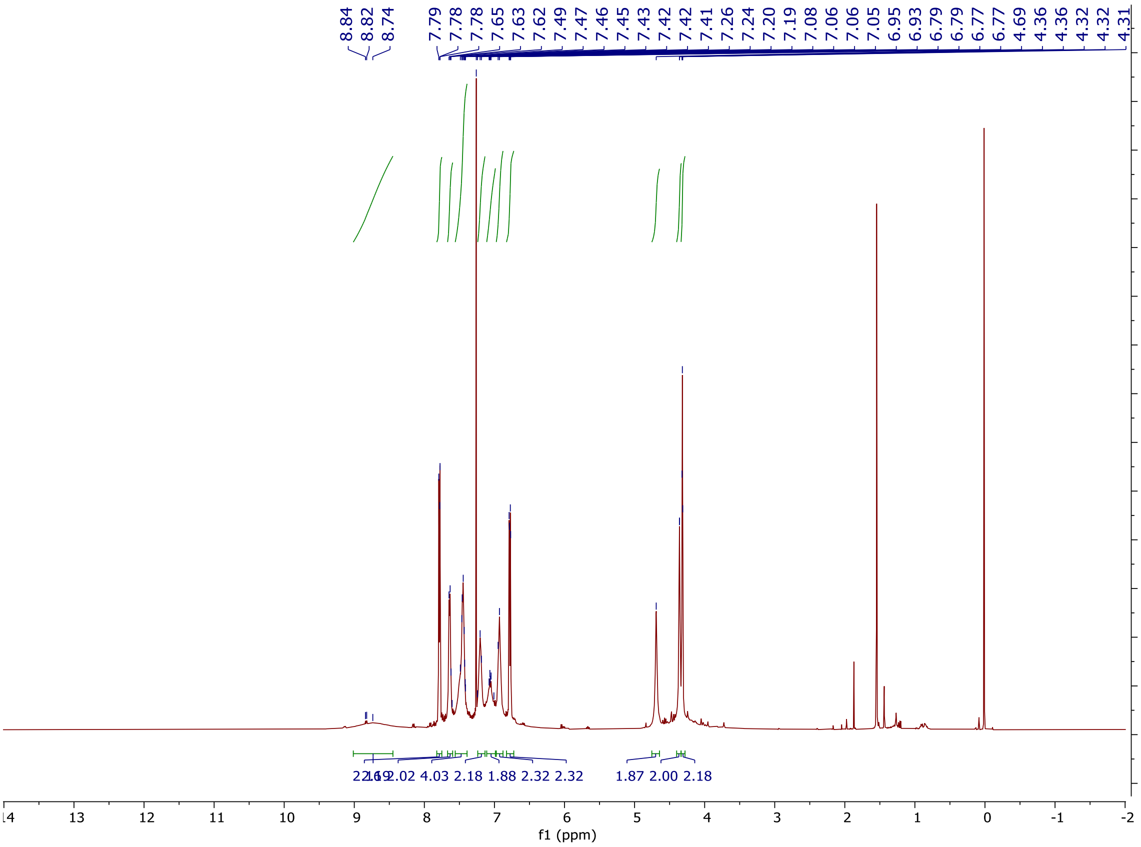

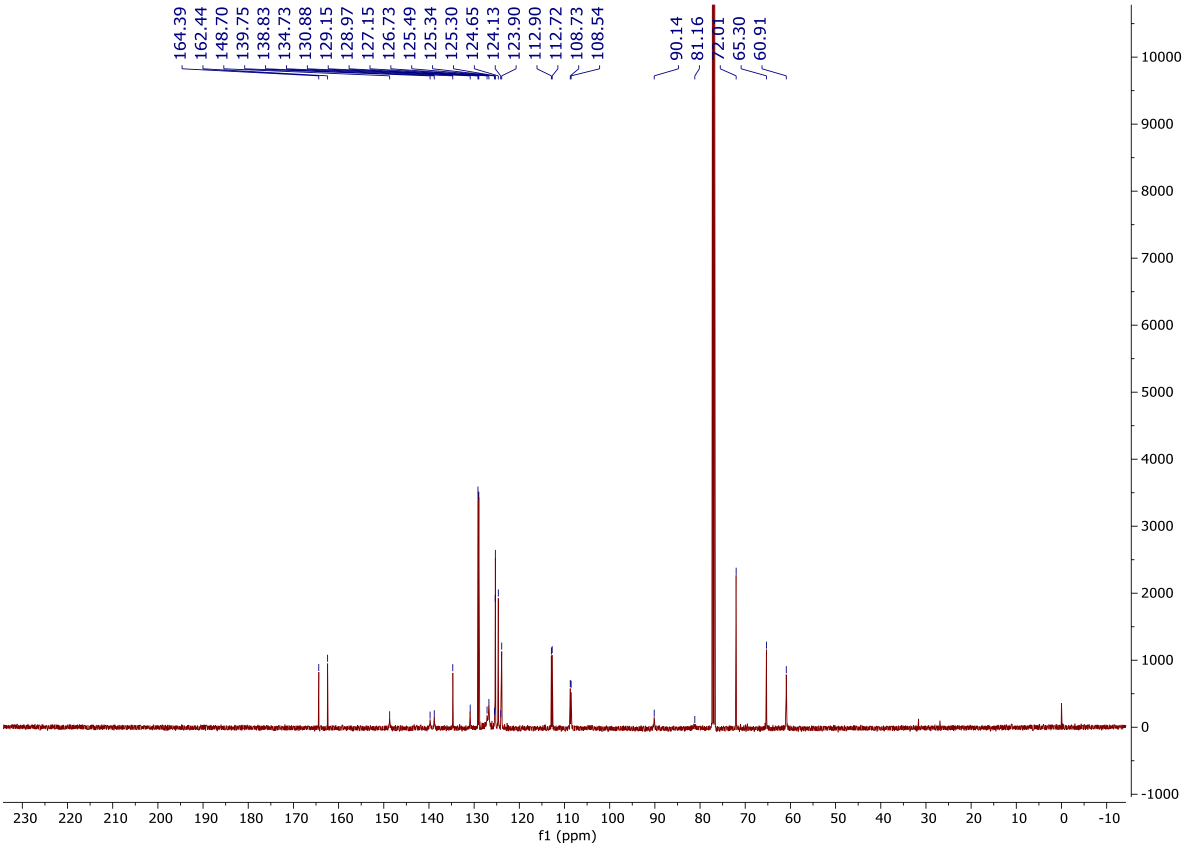

2-Cl, Naphthyl Diol **13a**

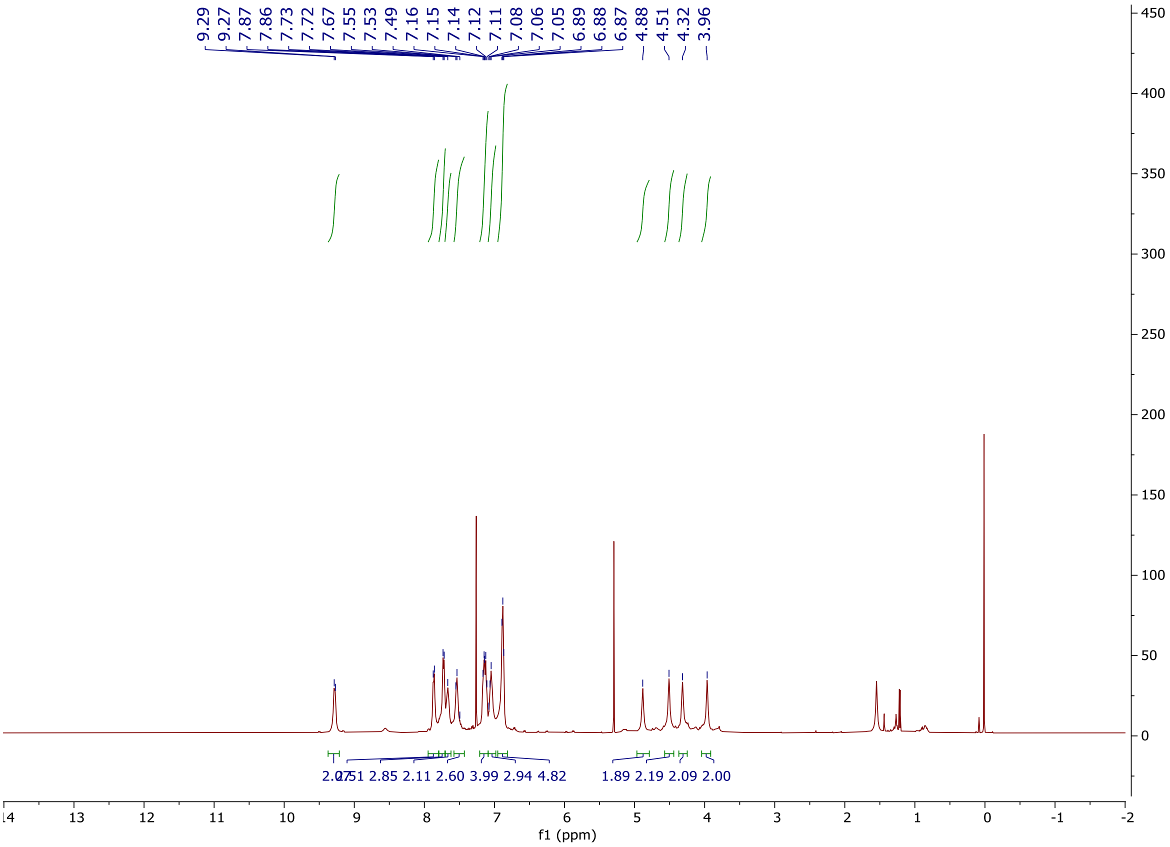

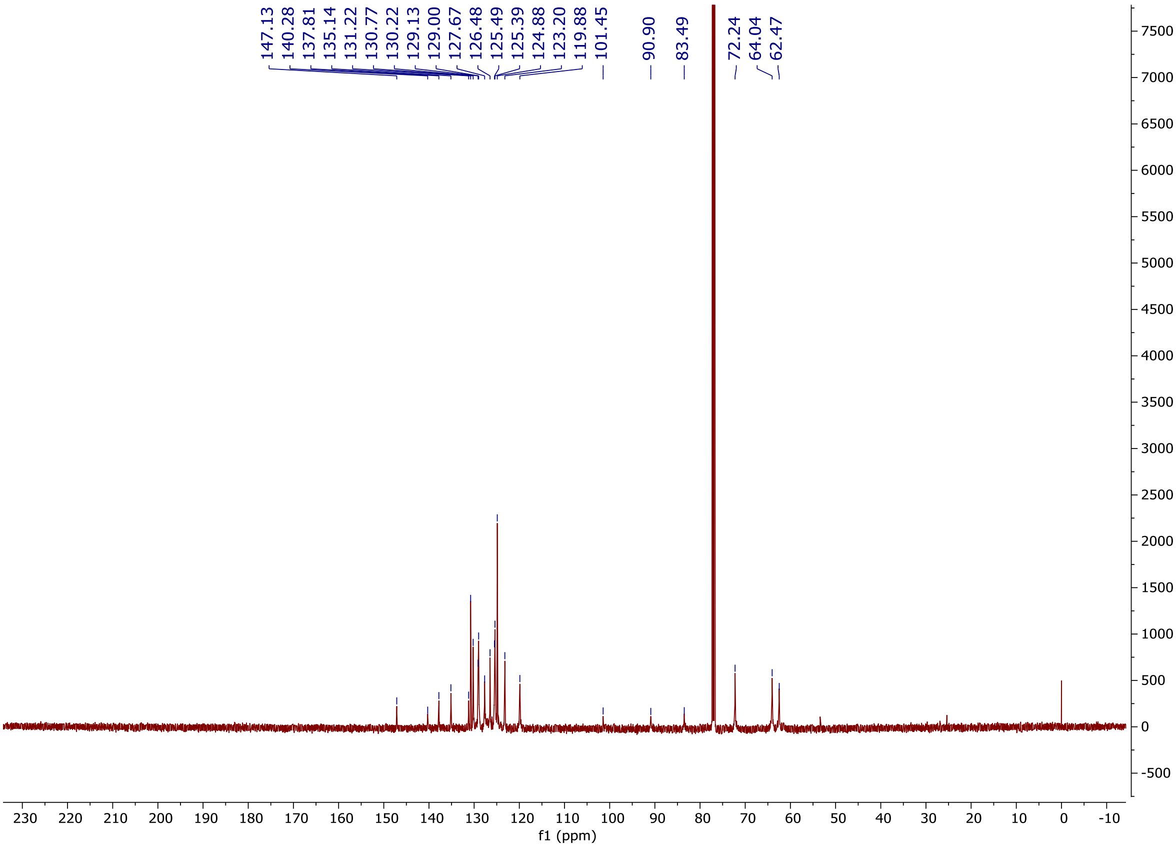

4-F Diketone **8e** racemic:

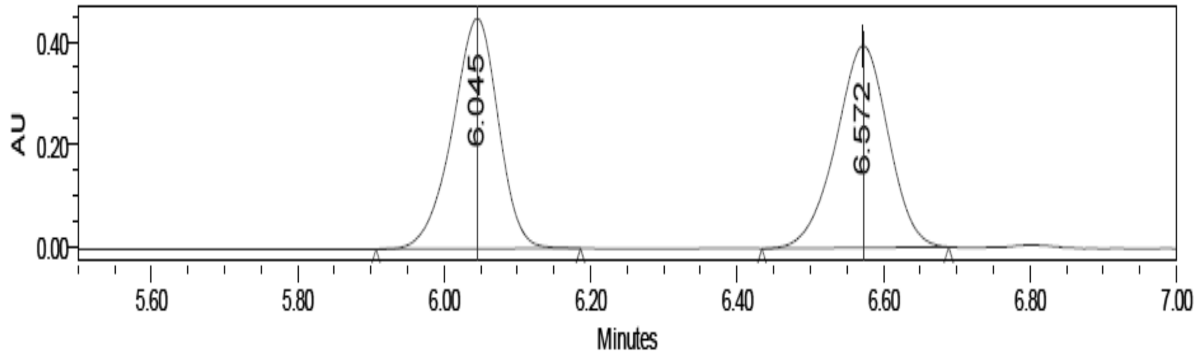

	Name	RT	Area	Height	Amount	Units	% Area
1		6.045	1958062	448624			49.97
2		6.572	1960337	392796			50.03

4-F Diketone **8e** asymmetric:

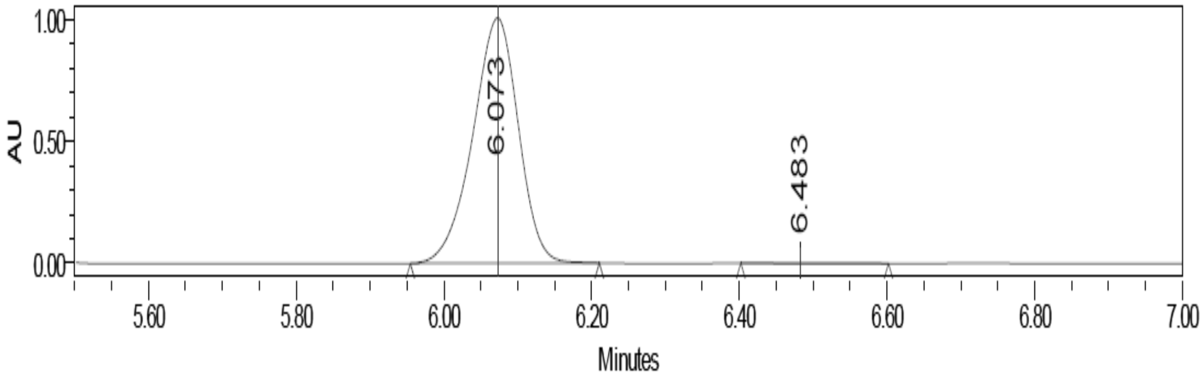

	Name	RT	Area	Height	Amount	Units	% Area
1		6.073	4320937	1007169			99.72
2		6.483	11919	-2083			0.28

2-Cl Diketone **8a** racemic:

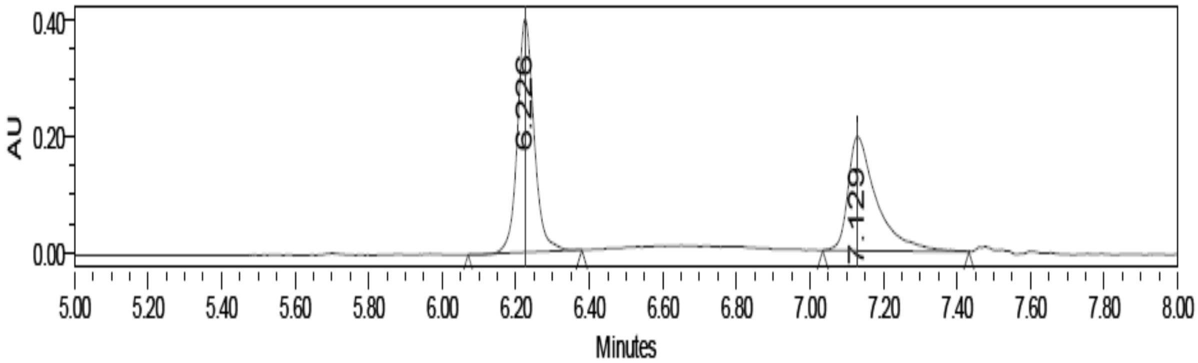

	Name	RT	Area	Height	Amount	Units	% Area
1		6.226	1263047	402399			54.18
2		7.129	1067961	196670			45.82

2-Cl Diketone **8a** asymmetric:

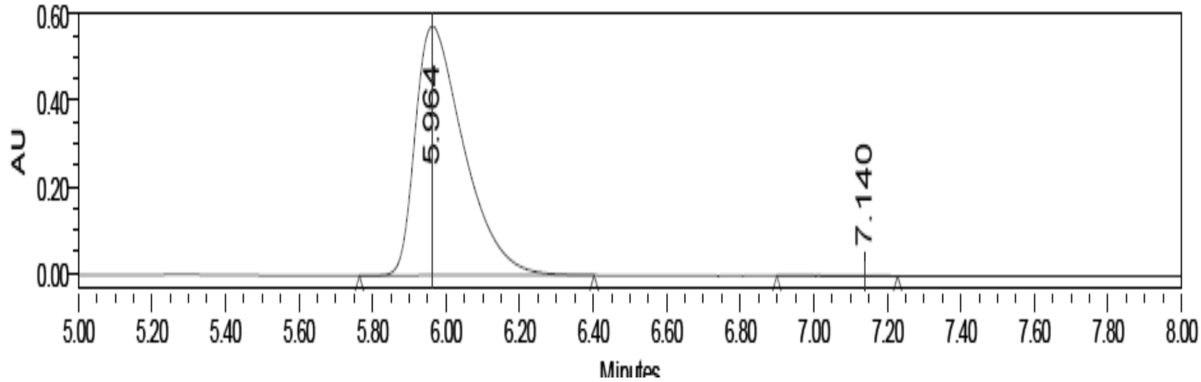

	Name	RT	Area	Height	Amount	Units	% Area
1		5.964	5293796	576970			99.94
2		7.140	2922	-347			0.06

2-Me Diketone **8c** racemic:

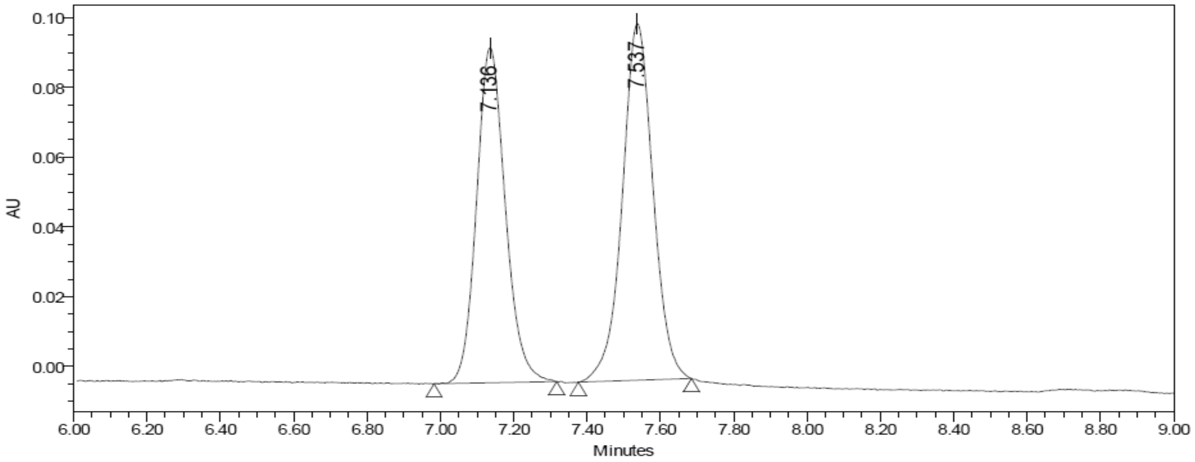

	Name	RT	Area	Height	Amount	Units	% Area
1		7.136	524750	96084			46.83
2		7.537	595714	102242			53.17

2-Me Diketone **8c** asymmetric:

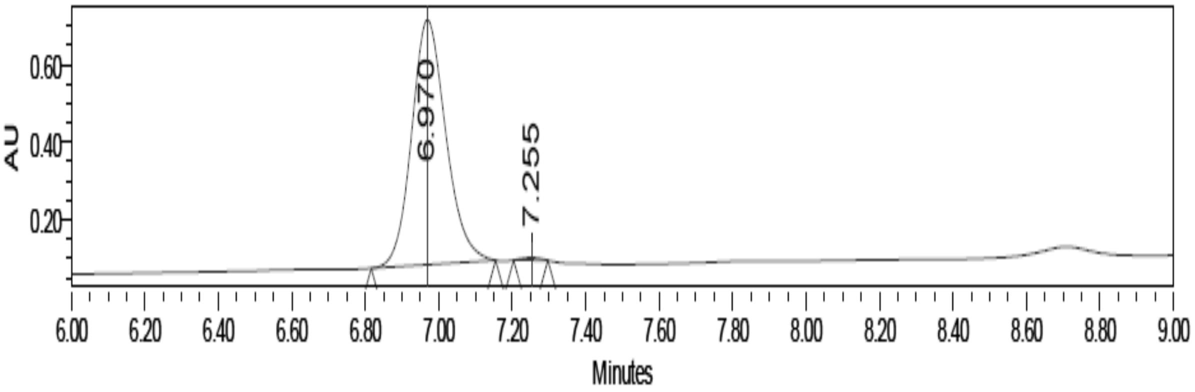

	Name	RT	Area	Height	Amount	Units	% Area
1		6.970	4104136	632339			99.41
2		7.255	24209	7130			0.59

3,4-OMe Ketone **8d** racemic:

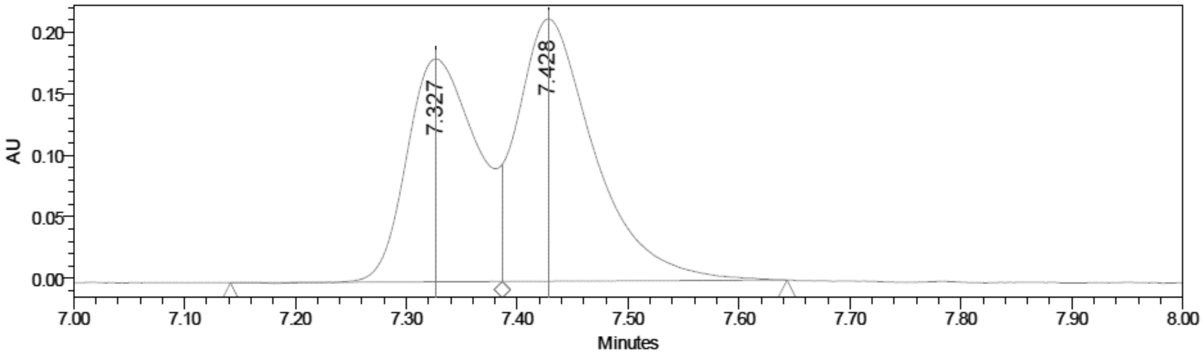

	Name	RT	Area	Height	Amount	Units	% Area
1		7.327	798831	181295			43.82
2		7.428	1023964	213250			56.18

3,4-OMe Ketone **8d** asymmetric:

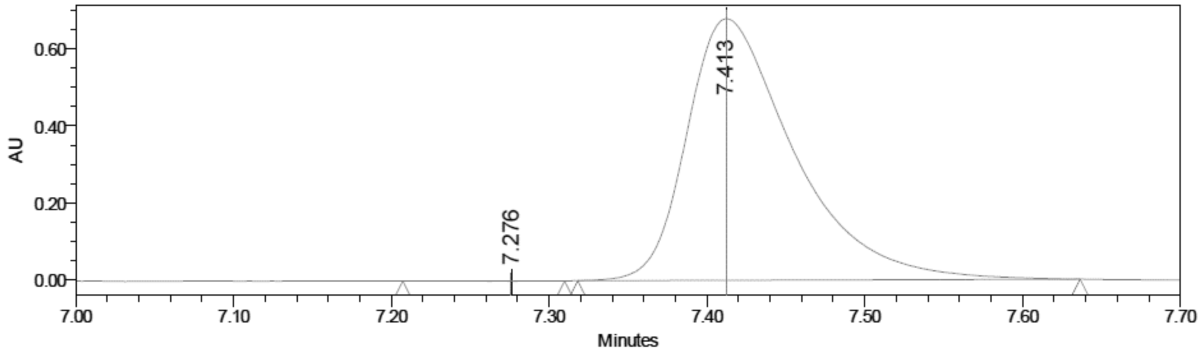

	Name	RT	Area	Height	Amount	Units	% Area
1		7.276	1826	-638			0.06
2		7.413	3212178	679353			99.94

(*S*)-2-(4-Chlorophenyl)-2,3-dihydro-4H-pyran-4-one

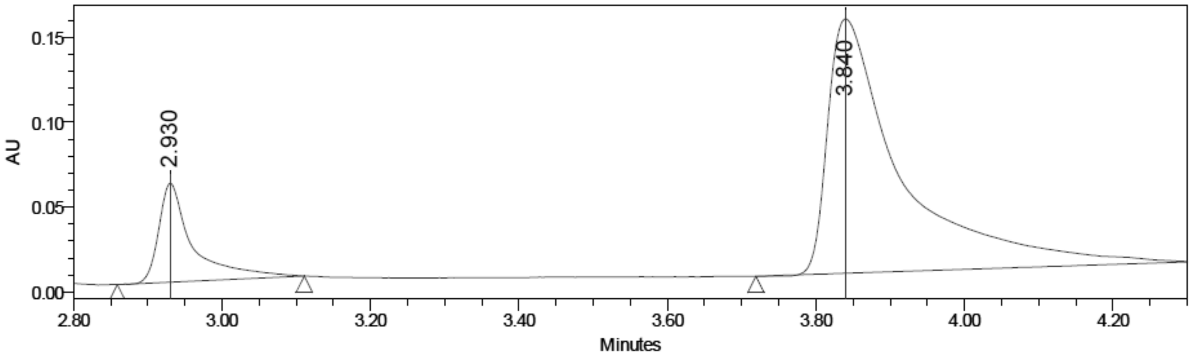

	Name	RT	Area	Height	Amount	Units	% Area
1		2.930	186190	58179			15.08
2		3.840	1048534	149681			84.92

(*R*)-2-(4-Chlorophenyl)-2,3-dihydro-4H-pyran-4-one

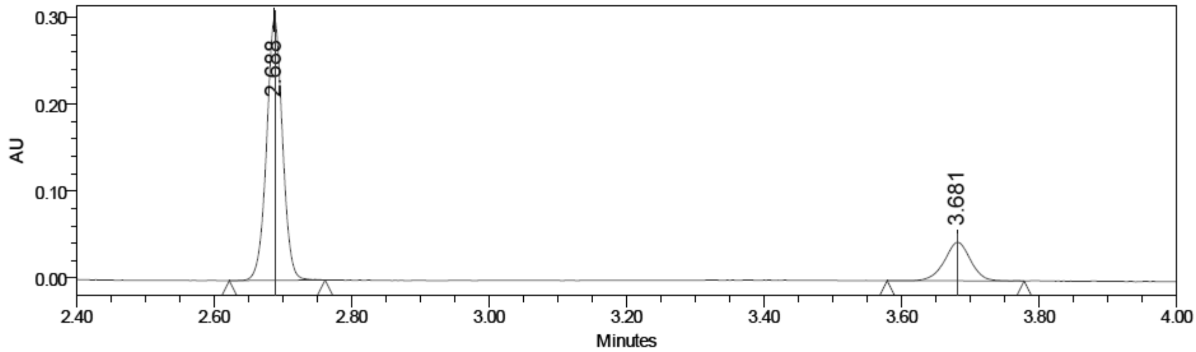

	Name	RT	Area	Height	Amount	Units	% Area
1		2.688	485731	300832			80.55
2		3.681	117317	44376			19.45

(*R*)-2-(4-Nitrophenyl)-2,3-dihydro-4H-pyran-4-one

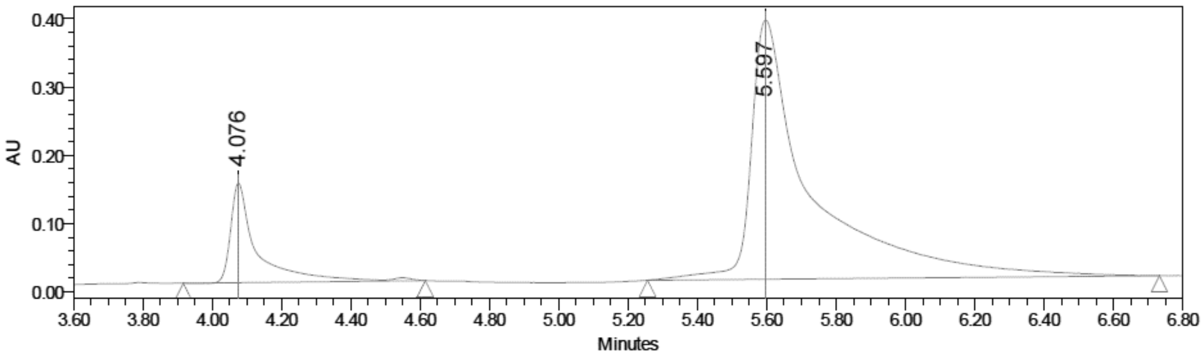

	Name	RT	Area	Height	Amount	Units	% Area
1		4.076	783623	145723			14.48
2		5.597	4628796	379426			85.52

(*S*)-2-(4-Nitrophenyl)-2,3-dihydro-4H-pyran-4-one

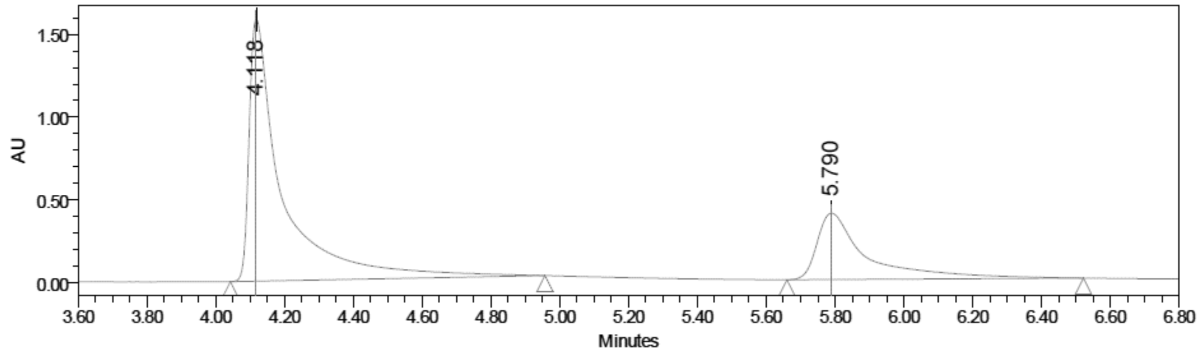

	Name	RT	Area	Height	Amount	Units	% Area
1		4.118	10742777	1581822			72.75
2		5.790	4024689	400538			27.25

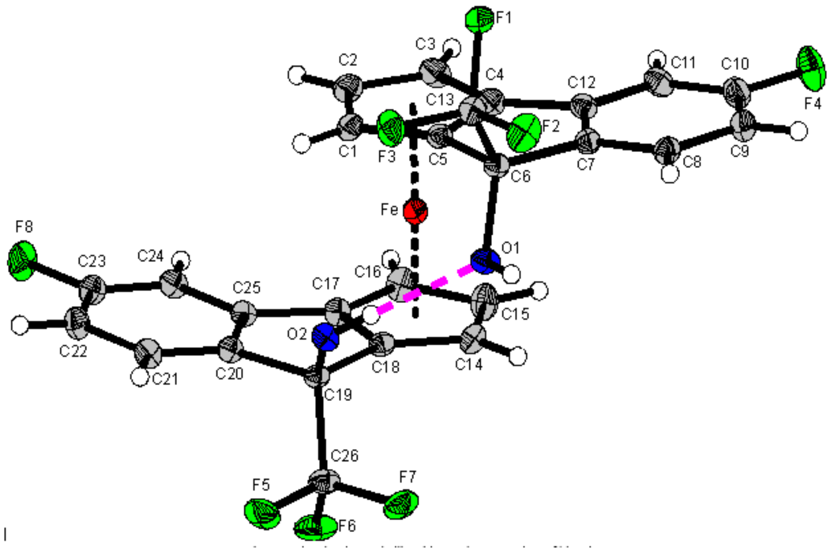

Crystal data and structure refinement for 4-F, CF_3 Diol **14e - CCDC 2209174**

Identification code	gui153	
Empirical formula	C_{26} H_{14} O_2 F_8 Fe	
Formula weight	566.22	
Temperature	100(2) K	
Wavelength	0.71073 Å	
Crystal system	Monoclinic	
Space group	I2 (#5)	
Unit cell dimensions	a = 15.8815(4) Å	a= 90°.
	b = 7.6405(2) Å	b= 105.397(2)°.
	c = 18.9856(4) Å	g = 90°.
Volume	2221.08(10) $Å^3$	
Z	4	
Density (calculated)	1.693 Mg/m^3	
Absorption coefficient	0.770 mm^{-1}	
F(000)	1136	
Crystal size	0.439 x 0.372 x 0.270 mm^3	
Theta range for data collection	2.889 to 33.045°.	
Index ranges	–24<=h<=23, –11<=k<=11, –28<=l<=28	
Reflections collected	35598	
Independent reflections	7735 [R(int) = 0.0307]	
Completeness to theta = 25.242°	99.8 %	
Absorption correction	Gaussian	
Max. and min. transmission	0.850 and 0.774	

Refinement method	Full–matrix least–squares on F^2
Data / restraints / parameters	7735 / 1 / 342
Goodness–of–fit on F^2	1.103
Final R indices [I>2sigma(I)]	R1 = 0.0306, wR2 = 0.0740
R indices (all data)	R1 = 0.0348, wR2 = 0.0771
Absolute structure parameter	–0.010(4)
Extinction coefficient	n/a
Largest diff. peak and hole	0.594 and –0.247 e.Å^{-3}

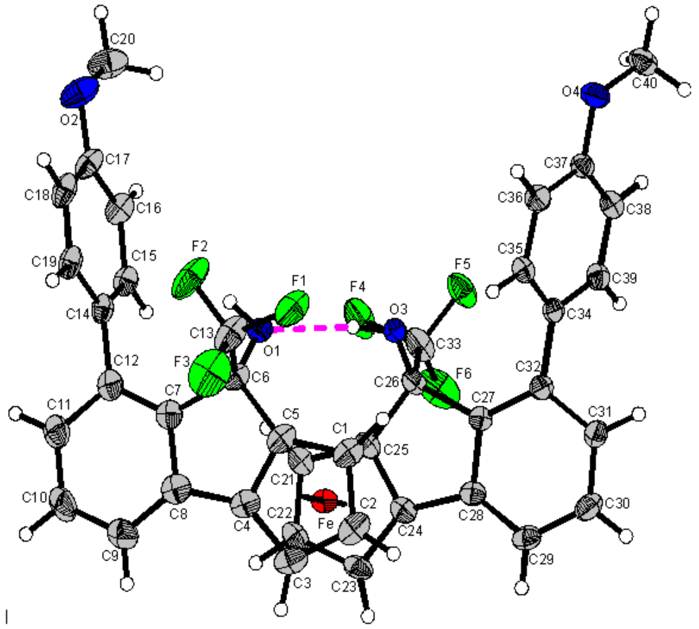

Crystal data and structure refinement for 2-*p*OMeC_6H_4, CF_3 Diol **14f - CCDC 2209179**

Identification code	gui149sqz	
Empirical formula	$C_{41} H_{32} O_5 F_6 Cl_2 Fe$	
Molecular formula	$C_{41} H_{32} O_5 F_6 Cl_2 Fe$ x $C H_2 Cl_2$ x $H_2 O$ [a)]	
Formula weight	845.41	
Temperature	100(2) K	
Wavelength	0.71073 Å	
Crystal system	Triclinic	
Space group	P1 (#1)	
Unit cell dimensions	a = 10.3824(2) Å	a= 110.493(2)°.
	b = 10.5405(2) Å	b= 93.413(2)°.
	c = 10.6436(2) Å	g = 116.688(2)°.
Volume	941.57(4) $Å^3$	
Z	1	
Density (calculated)	1.491 Mg/m^3	
Absorption coefficient	0.617 mm^{-1}	
F(000)	432	
Crystal size	0.337 x 0.302 x 0.265 mm^3	
Theta range for data collection	2.848 to 32.954°.	
Index ranges	–15<=h<=15, –15<=k<=16, –15<=l<=15	
Reflections collected	60613	
Independent reflections	12891 [R(int) = 0.0334]	
Completeness to theta =	99.8 %	

25.242°	
Absorption correction	Gaussian
Max. and min. transmission	0.919 and 0.895
Refinement method	Full–matrix least–squares on F^2
Data / restraints / parameters	12891 / 3 / 464
Goodness–of–fit on F^2	1.033
Final R indices [I>2sigma(I)]	R1 = 0.0376, wR2 = 0.0878
R indices (all data)	R1 = 0.0442, wR2 = 0.0933
Absolute structure parameter	–0.004(4)
Extinction coefficient	n/a
Largest diff. peak and hole	0.637 and –0.322 e.Å^{-3}

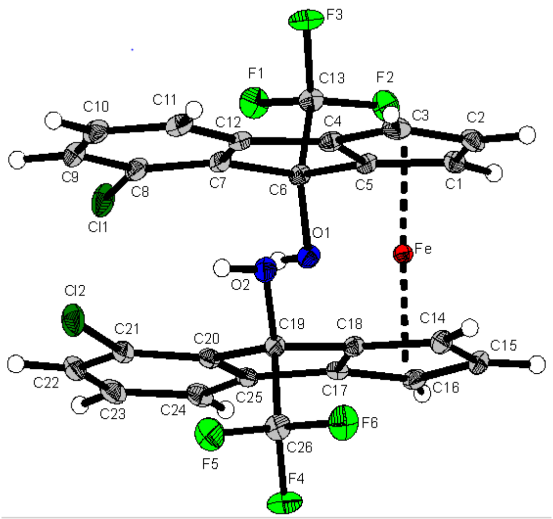

Crystal data and structure refinement for 2-Cl CF_3 Diol **14a - CCDC 2209176**

Identification code	gui156	
Empirical formula	$C_{26} H_{14} O_2 F_6 Cl_2 Fe$	
Formula weight	599.12	
Temperature	100(2) K	
Wavelength	0.71073 Å	
Crystal system	Orthorhombic	
Space group	$P2_12_12_1$ (#19)	
Unit cell dimensions	a = 8.2845(2) Å	a= 90°.
	b = 9.3188(2) Å	b= 90°.
	c = 28.6249(6) Å	g = 90°.
Volume	2209.89(9) $Å^3$	
Z	4	
Density (calculated)	1.801 Mg/m^3	
Absorption coefficient	1.001 mm^{-1}	
F(000)	1200	
Crystal size	0.348 x 0.296 x 0.239 mm^3	
Theta range for data collection	2.841 to 32.838°.	
Index ranges	–12<=h<=12, –13<=k<=14, –42<=l<=41	
Reflections collected	55727	
Independent reflections	7831 [R(int) = 0.0413]	
Completeness to theta = 25.242°	99.7 %	

Absorption correction	Gaussian
Max. and min. transmission	0.845 and 0.787
Refinement method	Full–matrix least–squares on F^2
Data / restraints / parameters	7831 / 0 / 342
Goodness–of–fit on F^2	1.079
Final R indices [I>2sigma(I)]	R1 = 0.0300, wR2 = 0.0636
R indices (all data)	R1 = 0.0364, wR2 = 0.0674
Absolute structure parameter	–0.016(4)
Extinction coefficient	n/a
Largest diff. peak and hole	0.397 and –0.308 e.$Å^{-3}$

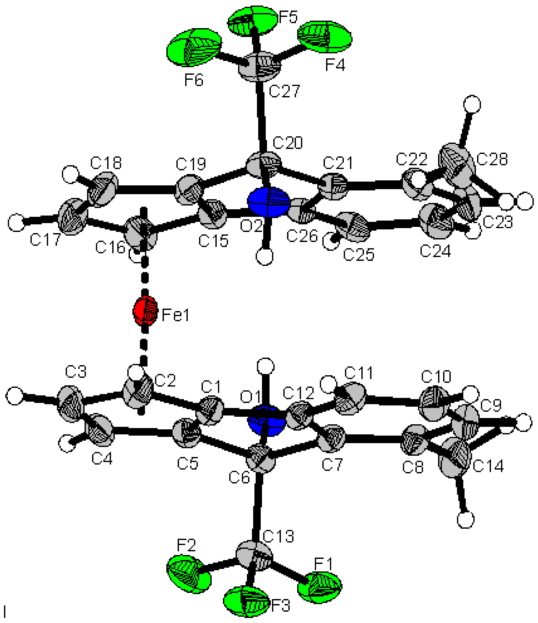

Crystal data and structure refinement for 2-Me CF_3 Diol **14c - CCDC 2209175**

Identification code	gui188	
Empirical formula	C_{28} H_{20} O_2 F_6 Fe	
Formula weight	558.29	
Temperature	170(2) K	
Wavelength	0.71073 Å	
Crystal system	Triclinic	
Space group	P1 (#1)	
Unit cell dimensions	a = 7.6853(2) Å	a= 90.741(2)°.
	b = 9.6977(2) Å	b= 98.369(2)°.
	c = 15.8961(3) Å	g = 91.050(2)°.
Volume	1171.77(5) Å3	
Z	2	
Density (calculated)	1.582 Mg/m3	
Absorption coefficient	0.717 mm–1	
F(000)	568	
Crystal size	0.250 x 0.175 x 0.118 mm3	

Theta range for data collection	2.801 to 32.937°.
Index ranges	–11<=h<=11, –14<=k<=13, –23<=l<=23
Reflections collected	45250
Independent reflections	15859 [R(int) = 0.0311]
Completeness to theta = 25.242°	99.8 %
Absorption correction	Gaussian
Max. and min. transmission	0.559 and 0.320
Refinement method	Full–matrix least–squares on F2
Data / restraints / parameters	15859 / 3 / 675
Goodness–of–fit on F2	1.044
Final R indices [I>2sigma(I)]	R1 = 0.0377, wR2 = 0.0793
R indices (all data)	R1 = 0.0462, wR2 = 0.0851
Absolute structure parameter	–0.006(4)
Extinction coefficient	n/a
Largest diff. peak and hole	0.405 and –0.361 e.Å–3

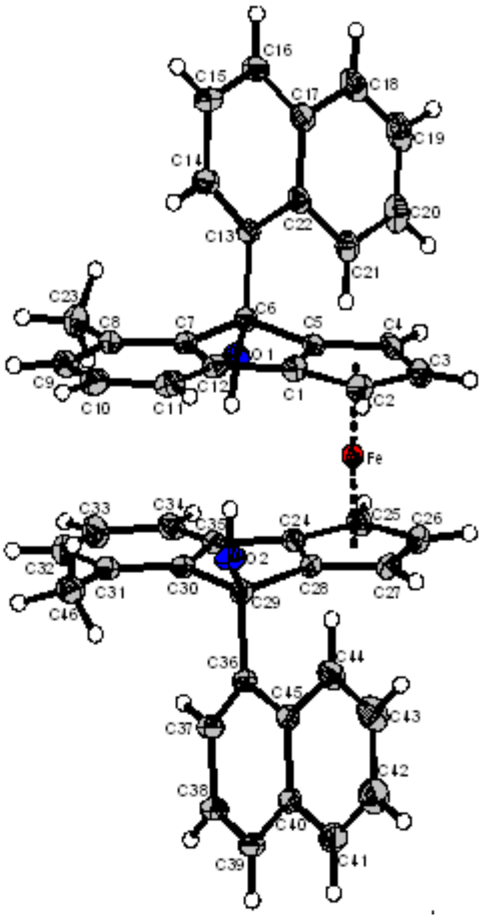

Crystal data and structure refinement for 2-Me Naphthyl Diol **13c - CCDC 2209179**

Identification code	gui189	
Empirical formula	C_{47} H_{35} O_2 Cl_3 Fe	
Formula weight	793.95	
Temperature	150(2) K	
Wavelength	0.71073 Å	
Crystal system	Monoclinic	
Space group	$P2_1$ (#4)	
Unit cell dimensions	a = 12.6854(3) Å	a= 90°.
	b = 11.9570(2) Å	b= 112.609(2)°.
	c = 13.1868(3) Å	g = 90°.
Volume	1846.45(7) Å3	
Z	2	

Density (calculated)	1.428 Mg/m3
Absorption coefficient	0.666 mm–1
F(000)	820
Crystal size	0.372 x 0.233 x 0.145 mm3
Theta range for data collection	2.840 to 32.907°.
Index ranges	–18<=h<=19, –18<=k<=17, –19<=l<=19
Reflections collected	57358
Independent reflections	12692 [R(int) = 0.0352]
Completeness to theta = 25.242°	99.7 %
Absorption correction	Gaussian
Max. and min. transmission	0.925 and 0.843
Refinement method	Full–matrix least–squares on F2
Data / restraints / parameters	12692 / 1 / 482
Goodness–of–fit on F2	1.072
Final R indices [I>2sigma(I)]	R1 = 0.0418, wR2 = 0.1001
R indices (all data)	R1 = 0.0501, wR2 = 0.1070
Absolute structure parameter	–0.015(4)
Extinction coefficient	n/a
Largest diff. peak and hole	0.888 and –0.601 e.Å–3

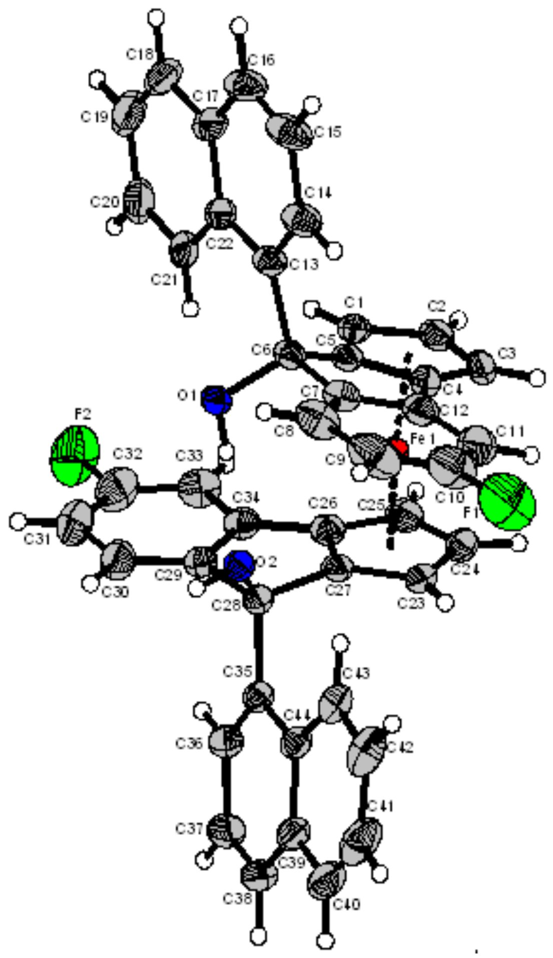

Crystal data and structure refinement for 4-F, Naphthyl Diol **13e - CCDC 2209177**

Identification code	gui173	
Empirical formula	$C_{44.41}$ $H_{28.82}$ O_2 F_2 $Cl_{0.81}$ Fe	
Molecular formula	C_{44} H_{28} O_2 F_2 Fe x 0.41 (C H_2 Cl_2)	
Formula weight	717.12	
Temperature	150(2) K	
Wavelength	1.54184 Å	
Crystal system	Hexagonal	
Space group	$P6_1$ (#169)	
Unit cell dimensions	a = 13.33743(8) Å	a= 90°.
	b = 13.33743(8) Å	b= 90°.
	c = 66.0349(4) Å	g = 120°.
Volume	10172.98(14) Å3	

Z	12
Density (calculated)	1.405 Mg/m3
Absorption coefficient	4.565 mm–1
F(000)	4429.1
Crystal size	0.256 x 0.225 x 0.159 mm3
Theta range for data collection	3.827 to 77.082°.
Index ranges	–16<=h<=16, –16<=k<=16, –83<=l<=83
Reflections collected	119088
Independent reflections	14271 [R(int) = 0.0340]
Completeness to theta = 67.684°	100.0 %
Absorption correction	Gaussian
Max. and min. transmission	0.657 and 0.549
Refinement method	Full–matrix least–squares on F2
Data / restraints / parameters	14271 / 1 / 915
Goodness–of–fit on F2	1.035
Final R indices [I>2sigma(I)]	R1 = 0.0342, wR2 = 0.0874
R indices (all data)	R1 = 0.0351, wR2 = 0.0886
Absolute structure parameter	0.002(2)
Extinction coefficient	n/a
Largest diff. peak and hole	0.760 and –0.491 e.Å–3

References

(1) Gohier, F.; Castanet, A. S.; Mortier, J. Ortholithiation of Unprotected Benzoic Acids: Application for Novel 2-Chloro-6-Substituted Benzoic Acid Syntheses. *Synth. Commun.* **2005**, *35*, 799–806.

(2) Nottingham, C.; Müller-Bunz, H.; Guiry, P. J. A Family of Chiral Ferrocenyl Diols: Modular Synthesis, Solid-State Characterization, and Application in Asymmetric Organocatalysis. *Angew. Chemie Int. Ed.* **2016**, *55*, 11115–11119.
